# In rice splice variants that restore the reading frame after frameshifting indel introduction are common, often induced by the indels and sometimes lead to organism-level rescue

**DOI:** 10.1371/journal.pgen.1010071

**Published:** 2022-02-18

**Authors:** Yanxiao Jia, Chao Qin, Milton Brian Traw, Xiaonan Chen, Ying He, Jing Kai, Sihai Yang, Long Wang, Laurence D. Hurst

**Affiliations:** 1 State Key Laboratory of Crop Genetics and Germplasm Enhancement, Nanjing Agricultural University, Nanjing, China; 2 State Key Laboratory of Pharmaceutical Biotechnology, School of Life Sciences, Nanjing University, Nanjing, China; 3 The Milner Centre for Evolution, Department of Biology and Biochemistry, University of Bath, Bath, United Kingdom; University of Minnesota, UNITED STATES

## Abstract

The introduction of frameshifting non-3n indels enables the identification of gene-trait associations. However, it has been hypothesised that recovery of the original reading frame owing to usage of non-canonical splice forms could cause rescue. To date there is very little evidence for organism-level rescue by such a mechanism and it is unknown how commonly indels induce, or are otherwise associated with, frame-restoring splice forms. We perform CRISPR/Cas9 editing of randomly selected loci in rice to investigate these issues. We find that the majority of loci have a frame-restoring isoform. Importantly, three quarters of these isoforms are not seen in the absence of the indels, consistent with indels commonly inducing novel isoforms. This is supported by analysis in the context of NMD knockdowns. We consider in detail the two top rescue candidates, in *wax deficient anther 1* (*wda1*) and *brittle culm* (*bc10*), finding that organismal-level rescue in both cases is strong but owing to different splice modification routes. More generally, however, as frame-restoring isoforms are low abundance and possibly too disruptive, such rescue we suggest to be the rare exception, not the rule. Nonetheless, assuming that indels commonly induce frame-restoring isoforms, these results emphasize the need to examine RNA level effects of non-3n indels and suggest that multiple non-3n indels in any given gene are advisable to probe a gene’s trait associations.

## Author summary

As protein coding genes are read in units of three (codons), insertions or deletions (indels) that are not a multiple of three long (non 3n) are expected to be especially harmful. Whether they are is important both for interpreting the results of non-3n indel experiments to probe a gene’s functional importance and for diagnostics. Particularly enigmatic are incidences where some non-3n changes in a gene compromise phenotypes while other seemingly comparable ones do not. One explanation for the latter is that a non-3n indel might be rescued via a frame-restoring splice form. Here we examine this hypothesis by inducing non-3n indels in many genes in rice and find that many non-3n indels are associated with a splice form that restores the reading frame. In the majority of these cases the indel appears to induce the potential rescuing splice form. We examine two top hit cases in detail and show functional rescue by splice modification. More generally, the frame-restoring forms are, however, low abundance and probably result in compromised proteins. We conclude then that splice mediated rescue is possible, but probably uncommon. Nonetheless it should not be overlooked in experimental design and interpretation.

## Introduction

The introduction of disruptive mutations, such as non-3n indels, is a common tool for defining gene-phenotype associations, be this for fundamental or applied research (e.g. identifying yield-associated traits [1]). In this context, there has been extensive discussion of the reasons that such potentially disruptive mutations vary so much in their fitness or phenotypic effects [2–5]. Discussion has, however, predominantly been focused on understanding between-gene variation [6] and relatively little consideration has been given to understanding variation in effects between non-3n indels within the same gene. For example, a one bp insertion in rice gene *OsIAA23* is predicted to experience premature termination and thus to have strong phenotypic defects, like comparable mutations in the same gene [7], but is of normal fertility [7]. Similarly, as we show here, some non-3n indels in certain rice genes are associated with normal phenotypes while seemingly comparable indels are not. High variance in indel effects suggests the possibility of false negative hits, i.e. key genes for a given phenotype are missed owing to rescue of the non-3n indel. Understanding the causes of this is also important for genetic diagnostics.

False negatives may well go overlooked as a lack of phenotype on knockdown tends to be common [2–5]. Broadly explanations for a lack of knockout fitness or phenotypic effects can be classified as focusing on the environmental conditionality of a gene’s functioning [5,8,9], intrinsic small contributions to fitness/phenotype of the gene [10–12] or compensation by other genes. The latter may be owing to paralogs [5,13–15] or sequence unrelated ones enabling parallel pathways [5,16,17]. These explanations, however, provide no suggestion as to why different non-3n indels in the same gene might have large disparity in fitness effects.

Some explanations for between-indel variation and possible false negatives, may be relatively trivial. For example, a small non-3n indel towards the 3’ end, and caught early by a newly in frame stop codon, should only cause limited disruption to the protein’s C terminus. Such an indel may well have a lesser effect than a comparable indel more 5’ as the later would be more likely to, for example, be associated with a much abbreviated protein. Indel size too might be important. A large indel may well ablate too much of a protein for the protein to ever be functional no matter what.

Are, however, non-3n indels occurring relatively early in a protein necessarily highly damaging? Non-canonical splicing leading to recovery of the reading frame, has been considered a possible explanation for rescue of some non-3n indels [18–20]. There has indeed been a series of analyses, many CRISPR based, supporting the possibility of non-canonical splicing of genes with frameshifting indels [7,18–26].

There may be many different means by which indels associated with non-canonical splicing could enable rescue. Indels may directly or indirectly modify levels of potential rescue isoforms. An indel might, for example, disrupt the splicing process by ablation of exonic splice enhancer motifs [27–31] giving high doses of potential novel rescue forms ([32]). Equally, non-3n indels may also create in frame stop codons that could induce exon skipping via nonsense associated splicing (NAS) [32,33]. Recent estimates suggests that 6–30% of nonsense mutations indeed induce splice modification[32]. By either mechanism, a non-3n indel within a 3n exon, could induce exon skipping, thought to be the most common mode of alternative splicing associated with CRISPR [23,25], producing relatively high levels of a rescue form (e.g. one missing only a small exon but otherwise retaining the reading frame). Examples of this sort of process have been described [18–20].

**Fig 1:**
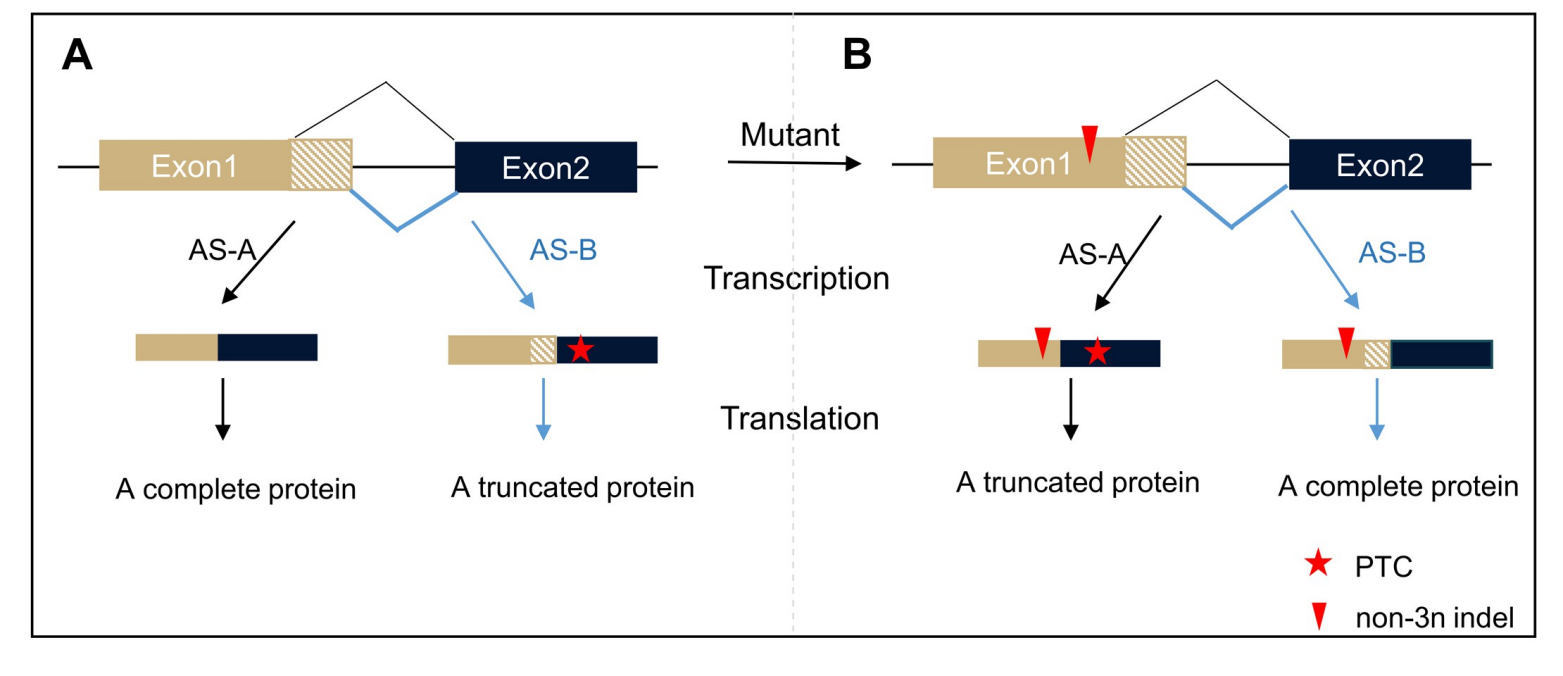
Predicted cryptic function of non-3n splice junctions. (**A**) In wild-type, the splice variant of AS-A could introduce a complete protein, while the AS-B may result in an incomplete truncated protein induced by premature termination codon (PTC).(**B**) In mutant with a non-3n indel, the splice variant of AS-A would introduce a truncated protein because of the frameshift mutation, while the AS-B may result in a complete protein because two non-3n nucleotide changes (non-3n indel and non-3n AS-B) eventually lead to a 3n change which would not induce frameshift.Black fold line indicates a splicing form AS-A, blue fold line indicates another splicing form AS-B which will introduce a non-3n nucleotide sequence comparing with AS-A.

Exon skipping need not be the exclusive mechanism. Consider two splice forms, wild type AS-A and a reading frame altering form, AS-B (Fig 1). Under normal conditions AS-A results in a complete protein, while AS-B, were it to be produced, results in both a very different peptide downstream of the frame-altering splice junction and either a newly located termination codon or no termination codon. Following a frameshift mutation that occurs in exon 1 (Fig 1B), the AS-A isoform now results in a novel potentially truncated protein, while the AS-B isoform could produce a near complete wild-type protein. We expect differential fitness effects of 3n+1 and 3n+2 indels as the alternative exon will rescue only one frame. In this case how the potential rescue form might achieve high dosage is not so clear, but there can exist downstream feedback circuits that increase transcription rates given low levels of viable protein [34].

The importance of non-canonical splicing and its effects on phenotype are relatively poorly explored (for references see above). It is unknown how commonly indels might be associated with non-canonical frame restoring isoforms. It is also unknown whether such isoforms are upregulated by or induced by the indels, what might be called indel-associated splicing (IAS). If indels do modify levels of frame-restoring splice forms then analysis of precompiled transcriptomic resources will be uninformative and bespoke transcriptomics will be needed to interrogate each enigmatic indel. Whether such frame restoring isoforms can rescue fitness and phenotype of the whole multicellular organism is also relatively unexplored as examination in mature organisms is uncommon (for exception see e.g. [18]), analysis more commonly being performed on cell lines (e.g. [19,20]).

We address these issues using rice as a model organism, not least because interpretation of CRISPR knockouts is important for determining which genes that might be relevant as regards yield-associated traits [1]. Our aim is not to determine the detailed mechanistic basis of any splice disruption. Rather we seek to provide appraisal of commonality of possible processes and the diversity of possible modes.

Using CRISPR induced indels in randomly selected loci we ask both about the commonality of frame restoring isoforms and of indels being associated with the appearance of, or apparent upregulation of, such frame restoration. We find that the majority of indels are associated with a frame restoring isoform and, perhaps surprisingly, that the majority of frame restoring isoforms are seen exclusively in the presence of the indel, suggesting that indel associated altered splicing is common. Of the others, many show evidence that a prior splice form has its relative frequency increased in the presence of the indel, such an effect being consistent with the action of NMD in the wild type or upregulation in the indel condition. Of the remainder, examination of the presence of potentially frame restoring splice forms that are natively present finds these also to be common (most genes will have such an isoform) but typically low abundance. We provide close examination of the two top hits (defined by dosage increase on indel induction of a frame restoring form) in the mature rice plant and show that non-canonical splicing can indeed enable organism-level phenotypic or fitness rescue. These work by different routes.

Finally, we extend our analysis in two exploratory manners. First, considering relatively fast evolving genes we ask whether the rescue mechanism might also affect pathways of gene body evolution. Second, we ask whether non-canonical splicing might also explain some rescue of point mutations as well as indels.

These results indicate that false negatives are a real possibility and hence that usage of multiple non-3n indels in any given gene of interest should be employed, with bespoke RNA level analysis to explore instances in which no or little phenotypic disruption is witnessed. It also implies that the connection between non-3n indels and fitness or phenotypic parameters is more complex than might at first be assumed.

## Results

### Experimentally induced indels have frame restoring isoforms in the majority of cases

To address the possibility of non-canonical splicing mediating phenotypic effects of indels, we generated CRISPR/Cas9 rice mutants with frameshift inducing mutations. We targeted 130 randomly selected gene loci and successfully disrupted 98 of them (S1 Table). Transcripts at 73 mutant loci were amplified by RT-PCR and sequenced (S2 Table). In mutants of 73 loci with a non-3n indel, we found 3,123 non-canonical junctions (S3 Table) for an average of 42.8 splice variants per gene (range 0 to 308 junctions per gene), of which 1,994 (63.8%) were non-3n.

#### The majority of loci have a frame-restoring isoform

We defined non-canonical splice forms as potentially compensatory if the splice event restores the canonical frame so enabling usage of the canonical stop codon (i.e. frame restoration). This definition likely captures the great majority of isoforms that have the potential to increase fitness above that expected were there no non-canonical isoforms. Of the 3,123 non-canonical junctions only 231 were considered to be potentially compensatory.

We define a junction that on its own will possibly break the annotated reading frame (i.e. introducing non-3n changes in the major frame) in the absence of a non-3n indel as a non-3n junction. Given this, there is a broad diversity of potentially compensatory, frame restoring, isoforms observed (S1A Fig). For 53.4% (39/73) of these genes with potential compensation for a putative loss-of-function (LOF) event through non-canonical splicing (S3 Table), 56.4% (22 genes) can both compensate through non-3n splice junctions and 3n junctions that skip the indels, 30.8% (12 genes) compensated through non-3n splice junctions only, a further 12.8% (5 genes) compensated through 3n splice junctions only. In 42% (96/231 junctions) the indel is present in the frame restoring form while in 58% (135/231 junctions) the indel is part of a skipped exon or exon part. Those indels not present in the new splice form are no closer to exon boundaries than those that remain present (S1B Fig) (Mann-Whitney U test, *P* = 0.54). Assuming the 73 loci to be broadly representative this suggests that approximately 53% of genes have a splice form with the potential to rescue non-3n indels by virtue of restoring the frame.

#### The majority of frame-restoring isoforms are not (obviously) present in the wild type

Whenever the levels of frame-restoring isoform are seen to go up compared with that isoform in the absence of the indel, there are two (not necessarily mutually exclusive) explanations: the isoform was always there but in the absence of the indel NMD removes it or, the isoform is induced by the indel. As NMD requires a cytoplasmic pioneer round of translation, RT-PCR could detect even rare transcripts prior to their destruction by NMD. Given this, absence of an RT-PCR trace of an isoform in the absence of the indel, but a clear signal in the presence of the indel, would be supportive of the second model, i.e. that indels cause novel isoforms (much as in nonsense associated alternative splicing, NAS, nonsense mutations cause new isoforms). We thus ask whether we can detect isoforms in the absence of the indels (i.e. in wild type).

For 24 of all the potentially frame-restored genes we have obtained RT-PCR-seq data for both WT and the indel mutants (S1 Dataset). In the 24 genes, 118 of 157 (75%) frame restoring (rescue) junctions are not detected in WT (read = 0) (see S1 Dataset). While RT-PCR-seq should have high sensitivity, we also employ lower sensitivity RNA-Seq data in the same tissue and similar stage as the mutants as a means to double check the result (depth may be lower but the transcriptomic resources are broader). Such data is available for Kasalath, TP309, the same cultivar as the mutants and from Nipponbare. There is 92GB of data available for the former and 308GB data for the latter. For such RNA-seq data in the same tissue, 35 genes covered by sequencing, 211 of 215 (98.1%) frame restoring junctions are not detected in WT (read = 0) (S1 Dataset). Together these data support the hypothesis that indel-associated alternative splicing (IAS) is common. Notice that we do not consider this as evidence of any sort of adaptive mechanism. It seems just as plausible that non 3n indels will also induce non-frame restoring isoforms and 3n indels are likely to also induce frame breaking splice forms (S1 Dataset). As our focus is on whether non 3n indels might be rescued by frame restoration we have not analysed these instances.

Conversely, for those cases where we can detect the frame restoring isoform in the wild type (RT-PCR-seq read >0), if the indel is preserved in the frame restoring form we can devise PCR primers to differentiate the source of the isoforms. With these we can test the hypothesis that the wild type allele can generate the non-canonical isoform both in wt homozygous and in wt/indel heterozygotes. We indeed observe that some of the non-canonical isoform is from the wt allele in the wt/indel heterozygote (we caution against over-interpretation of this data as amplification biases affect relative proportions in heterozygotes). This implies repeatability of evidence (S4 Table).

The latter is a special case of transcripts that exist prior to the introduction of the indel. In some cases these too might be at raised frequencies after indel introduction. Of 39 that have “rescue junctions” present both in WT and mutant, from RT-PCR-seq we determine *R*_mw_ = “the relative level of the rescue junction in mutant (R_m_)”/ “the relative level of the rescue junction in WT (R_w_)”. Relative level is defined as read-depth of isoform of interest / read-depth of all isoforms within the junction region (junction region depth). A *R*_mw_ value over 1 is consistent with increased relative abundance of the frame restoring isoform in the presence of the indel. The 118 junctions absent in the wild type and seen after indel introduction have *R*_w_ = 0 and so *R*_mw_ = ∞ if *R*_mw_ > 0. *R*_mw_ > 1 when *R*_w_ > 0 implies an increase in relative abundance that may be owing to an absolute increase (as with those with *R*_w_ = 0) or potentially no absolute change, just a relative change as the alternative isoforms may be removed by NMD or a combination of both.

Besides 4 junctions in wt/mutant heterozygotes and biallelic (+1 bp/-19 bp) individuals in which we could not rule out the possibility that the “rescue” form was mostly contributed by the wt-allele, we find 20 of the 153 junctions have both *R*_w_ >0 and *R*_mw_>1, i.e. seen in the wild type but at an increased relative level after indel introduction. The remaining 14 junctions have *R*_w_ > 0 and *R*_mw_ ≤ 1, i.e. seen in the wild type but at lower relative levels in the indel condition. One junction has both *R*_mw_>1 and *R*_mw_ ≤ 1 in different genotypes. For the 35 junctions seen in the wild type (*R*_w_ > 0), the fold-changes range from 0.01 to 23,769, with a median of 1.41. Comparing cases with *R*_w_ = 0 and *R*_w_ > 0, there is no significant difference in the two classes as regards the distance from the indel to the exon boundary (Mann–Whitney U test, *P* = 0.15). We also see no differences between the two most extreme classes i.e. those with *R*_w_ = 0 and those with *R*_w_ > 0, *R*_mw_ < 1 (Mann Whitney U test, *P* = 0.41). All cases in which simple exon skipping removes the indel are associated with either new skipping or greatly increased relative rates (S5 Table).

These data suggest that amongst the frame restoring isoforms the great majority (91%) show either evidence of increase in absolute abundance (*R*_w_ = 0, *R*_mw_ = ∞) or relative abundance of the frame restoring form (*R*_w_ > 0, *R*_mw_ > 1) on introduction of the indel. Five genes (Os02g0553200, Os05g0170000, Os05g0418100, Os10g0471100 and Os10g0555700) have greatly increased relative abundance in the mutant (*R*_mw_ >>1) and relatively high (junction reads/ junction region depth>10%) levels of expression of these non-canonical splice variants following the CRISPR/Cas9 edited mutation (S6 Table).

#### Evidence that NMD cannot explain all upregulation of frame restoring forms

While the above is consistent with the notion that many isoforms only become visible post indel induction, this does not fully exclude the possibility that NMD might be acting in the wild type causing apparently low levels of the potential frame restoring form in the absence of the indel. Similarly, for those with increased relative abundance, this may be a direct effect of suppression of the rescue junction in the wild type. To examine this, we need to consider isoform levels also in the absence of NMD.

We attempted to generate knockout mutants of the NMD pathway but recovered no viable plants. We did however obtain two knock-down mutants miUPF1 and miUPF3 in the background of Kasalath using artificial miRNAs (amiRNA)[35]. Total RNAs were directly extracted from the induced callus of the two NMD-KD mutants. We used RT-PCR to amplify the transcripts of *HDAC6*. The individual transcripts in novel NMD-KD mutants were cloned with TA cloning. This gene had no rescue junctions in the wild type. The rescue junction (chr06:22123711–22126744) was also not detected in both WT and NMD-KD mutant calli (Table 1). As we could TA clone this gene in all samples, this is consistent with the hypothesis that the indel induces the frame restoring isoform. If this is typical of the other cases where the rescue junction was not found in the wild type, this suggests that indeed, indel induced splicing is commonplace.

**Table 1:** The ratio of rescue junction detected in KO mutants and WT and NMD-related gene silencing tissue.

	Potential rescue junction	Ratio of rescue junction				
Gene Locus		Prior target-gene KO experiments		NMD-KD experiments		
		WT-plant	mutant	WT-callus	miUPF1- callus	miUPF3- callus
*HDAC6*	chr06:22123711–22126744	0	2.1%	0/21	0/32	0/30
*WDA1*	chr10:17453792–17453945	1.8%	37.7%-56.5%	1/29	8/32	3/31

#### Many, but not all, frame restoring forms are likely to be functional

In the CRISPR-Cas9 induced indels we find that the majority of genes have possible rescue isoforms, i.e. frame restoring forms. Frame restoration alone, however, provides a (very) liberal definition of what a compensating splice form could be, namely any form that in effect preserves the use of the canonical stop codon. There are at least two further parameters contributing to the variance in fitness/phenotype both of which are hard to ascertain on *a priori* grounds. The first is the degree to which the frame restoring form also preserves the protein’s function. The second is the expression level of the potential rescue form.

To consider protein functionality, we consider protein similarity between wild type and frame restoring forms and assay the extent to which protein domains are preserved (S1 Dataset). Of the 39 genes investigated, two genes have no reliable domain detected. In the remaining 37 genes, we calculated how many of frame restoring forms would both preserve all domains and have overall similarity > 90%. We chose 90% as in yeasts orthologs with > 80–90% identity appear enriched for proteins that have their protein interaction network conserved (see Fig 4E in [36]), although overall identity tends not to predict compensatory ability. We found 37 such possible rescue forms, belonging to 26 junctions in 14 genes. This suggests that 37.8% of the 37 genes has at least one possible rescue splice form that preserves all domains and with similarity > 90% to the wild type. This supports the notion that variation in protein competence, even after non-canonical splicing enabling some level of a transcript terminating at the canonical stop codon, is likely to explain some variation in fitness of otherwise similar indels.

#### Dosage of frame restoring forms is commonly low but variable

The above calculations suggest that most genes would have potential rescue isoforms in so much as they have frame-restoring that preserve a good deal of the functional protein. The remaining contributor to between-indel variance is the relationship between the dose of the possible rescue form and resulting fitness. Given the uncertain nature of dose response curves this is in principle hard to know on *a priori* grounds.

One approach to estimate how many might have expression levels with relatively high fitness is to consider the asymptote of the function relating RT-PCR-seq read depth to the proportion of frame restoring isoforms that have that read depth or higher. Naturally, as the minimum read depth increases so the proportion of genes with a possible rescue form decreases: in 73 mutant loci, 45% (33/73) of genes have possible rescue forms if we impose a 10 read minimum, while this is 22% at a minimum of 500 reads. The function rapidly asymptotes after a minimum of 500 to an estimate of around 16% of genes (S2A Fig).

As this estimate of 16% relies on RT-PCR-seq data, it remains possible that the frame restoring isoforms remain relatively low abundance and thus don’t contribute much to fitness/phenotype. This is hard to appraise in absolute terms but we can ask about relative enrichment. In WT, the relative level of the classical junction is generally high at 87%~99.9% (averaged 97.6%, near 100%). Relative here is again defined with respect to all the isoforms in WT, i.e. “read-depth of major classical junction in WT” / “read-depth of all isoforms within the junction region (junction region depth) in WT”. We can extend the same relative measure to the rescue junctions in the mutant i.e. “read-depth of rescue junctions in mutant” / “junction region depth in mutant”. Only two putative rescue isoforms have a relative level over 0.5 (these being *wda1* and *bc10*), while another 5 have this ratio over 0.1 but lower than 0.5. The proportion of genes with these 7 rescue junctions is 8.2% (6/73). All the 7 junctions are with a minimum read depth of 65 (these accord with the 5 genes noted above to be strongly upregulated). Variation in level of the frame restoring isoform is thus likely to contribute to variance in fitness.

#### Full rescue is probably uncommon

The above estimates provide evidence for at least three parameters that relate indel effects via non-canonical splicing to variance in fitness/phenotype between indels, these being the existence of a non-canonical potential rescue splice form, the degree of conservation of protein function and the level of the rescue form. How often, we can ask might something approximating to full rescue be seen? As many indels have no frame restoring form, cases where full rescue is possible will be examples where the highest and lowest fitness of different indels in the same gene will be seen.

Even if the majority of isoforms show upregulation after the introduction of the indel, the above calculations taken *en masse* suggest that something approximating to full rescue by non-canonical splicing is likely to be an uncommon process. For the proportion of rescue junctions at anything approaching biologically important dosage levels, we can take the 16% asymptotic figure as an upper bound. To these estimates we combine our estimates of the proportion of loci with a frame restoring isoform (53%), of which 37.8% are probably intact enough to well conserve function. Assuming that in only about 16% of incidences is the expression high enough, then we can calculate the proportion of genes that could be nearly fully rescued by non-canonical splicing as 0.16 x 0.38 x 0.53 = ~3–4%. By contrast if we assume a 4% estimate for the proportion of rescue junctions with appreciable expression levels, then under 1% is a more realistic figure. These calculations assume the features are independent. Considering the change to the protein and the expression level, 4 of 73 (5.48%) genes have at least one frame restoring splice form that preserves all domains and with similarity > 90% to the wild type and the relative level of the rescue junction to all the transcript isoforms>10%. This suggests that the former calculation may be the more relevant. Either way, we suggest therefore that non-canonical full rescue is unlikely to be a common mode of full rescue, but will commonly contribute to between-indel within gene variation.

### Little evidence that IAS is a manifestation of NAS

It is to be expected that a non-3n indel, by causing a frameshift, will commonly have the effect of creating a new 3’ stop codon. Might then indel-associated alternative splicing (IAS) be a manifestation on nonsense-associated alternative splicing (NAS)? Following early evidence for PTCs causing non-canonical splicing [37], NAS was defined as an event whereby a new in frame stop codon (what would be a premature termination codon), causes the skipping of the exon containing the PTC [33]. The new stop codon could be owing to either a nonsense mutation or a 5’ frameshift mutation (e.g. a non-3n indel). This skipping might either be because some mode of scanning relays the presence of the PTC to the splicing machinery [38–40] or because the sequence context of the PTC no longer functions as an exonic splice enhancer (or related motif [33,41,42]). In our case, the sequence context of the PTC is unaltered (assuming the indel is far enough away) and so any association between IAS and NAS would more likely be owing to the proposed scanning mechanism. Indeed, some argue that scanning of PTCs leading to skipping of 3n exons may be adaptive as it could restore the frame [33,43].

**Fig 2:**
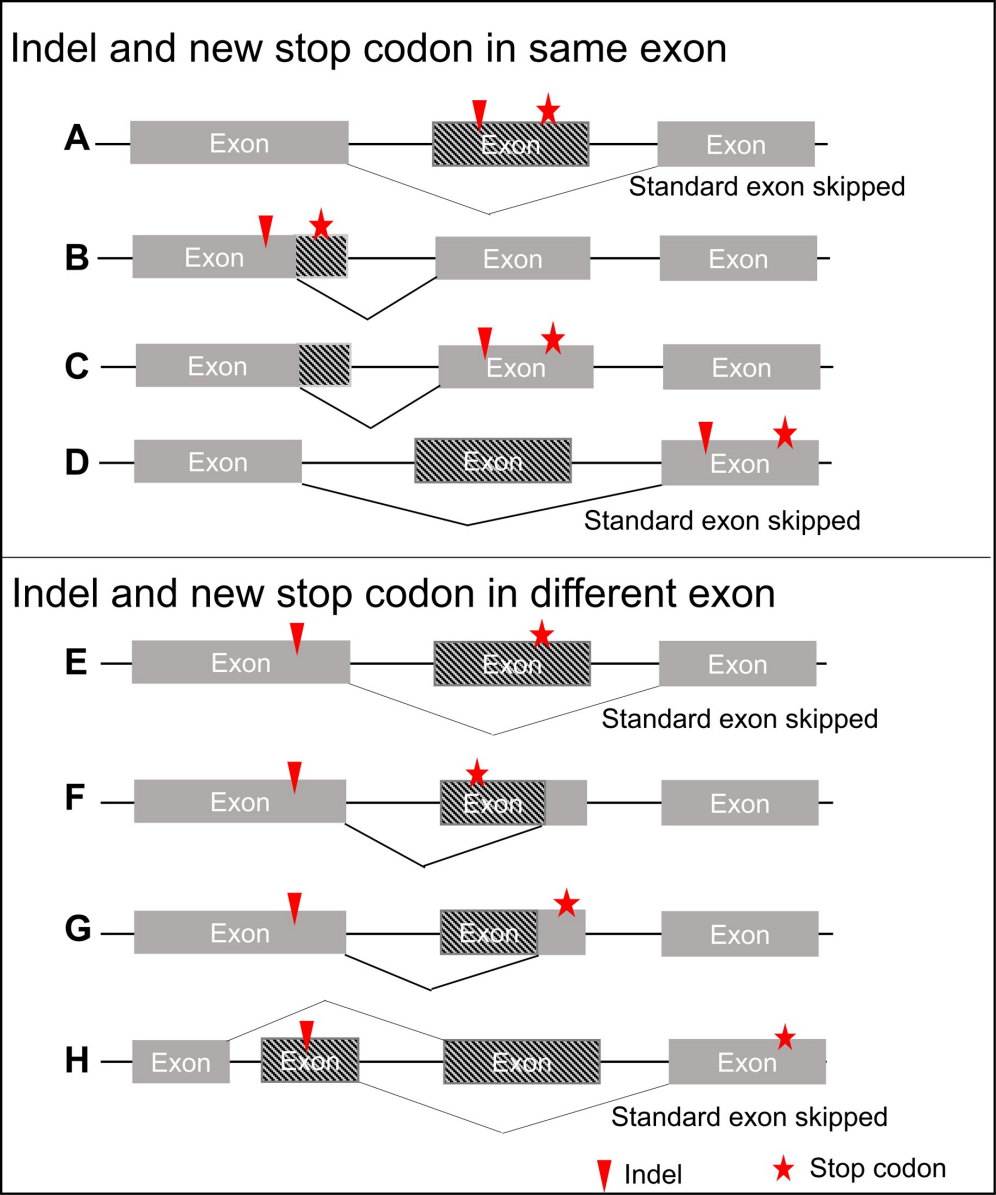
In-frame stop codons and modes of rescue. Different relationships between rescue junction and indel-introduced PTCs are shown. The red asterisks show the PTC introduced by the indels (red triangle) if not rescued. The polylines depict the rescue junctions which could either skip or compensate the PTC, resulted in preserving of original stop codons but with truncated proteins. **(A-D)** the indel and new PTC in the same exon (182 rescue events observed) **(A)** PTC skipped by standard exon skipping (5 rescue events); **(B)** PTC skipped by novel junction but not exon skipping (95 rescue events); **(C)** PTC not skipped but rescued by novel junctions (not exon skipping) (76 rescue events); **(D)** PTC not skipped but rescued by skipping of the exon before PTC (6 rescue events) **(E-H)** the indel and new PTC in different exons (84 rescue events observed) **(E)** PTC skipped by standard exon skipping (3 rescue events); **(F)** PTC skipped by novel junction but not exon skipping (40 rescue events); **(G)** PTC not skipped but rescued by novel junctions (not exon skipping) (38 rescue events); **(H)** PTC not skipped but rescued by skipping of the exon before PTC (3 rescue events). In (A), (B), (E), (F) the PTCs are excluded (n = 5+95+3+40 = 143 events), while (A) and (E) are through exon-skipping (n = 8 rescue events). In (C), (D), (G) and (H) the non-3n indels are compensated so the supposed PTCs will not actually happen.

Were IAS a manifestation of NAS we would expect to see many examples of the recue events being an exon with a PTC being skipped. We have thus scrutinized the mode of splice disruption and the location of new PTCs formed owing to the non-3n indel. There are 364 putative rescue events associated with non-3n indels which could restore the original stop codon and avoid new in-frame stop codons. Among them, 266 events were via rescue of the indel-introduced PTCs but only 8 of these were via exon-skipping of the PTC (see Fig 2). Of these 8, 5 showed skipping of the 3n exon only in the presence of the indel and PTC. We conclude that NAS induced exon skipping cannot be a common mode of rescue of indels.

One might argue that defining NAS in terms of exon skipping might be too restrictive. Indeed, in 135 instances we see part of the exon with the PTC, but not the whole exon, is not present in the putative rescue form. Whether this is more than expected by chance is hard to say as, by definition, we focus on cases where there is frame restoration, and hence avoidance of the PTC must be seen by definition. The remaining 123 act by compensating the indels by an equal but opposite frameshift manifested by splicing so the supposed PTCs will not happen. The remaining 98 were via rescue of the indel-introduced stop codons which would appear behind the original stop codon (so not premature). We conclude that NAS, if conceived as a mechanism associated with exon skipping [33], does not explain many incidences of IAS.

### Multiple splice routes to rescue

The above suggested that indels commonly induce possible rescue forms and in some cases these may be able to contribute significantly to rescue. In addition, we don’t expect all indels in the same gene to have equal effects on fitness. We now consider in greater detail the top candidates from rescue/variance effects, the cases of *wda1* and *bc10*, as these two show appreciable levels of the frame restoring isoforms. These we find operate by different splicing modes and can provide effective rescue.

#### Rescue of 3n+1 indels, but not 3n+2 indels, in *wda1*

**Fig 3:**
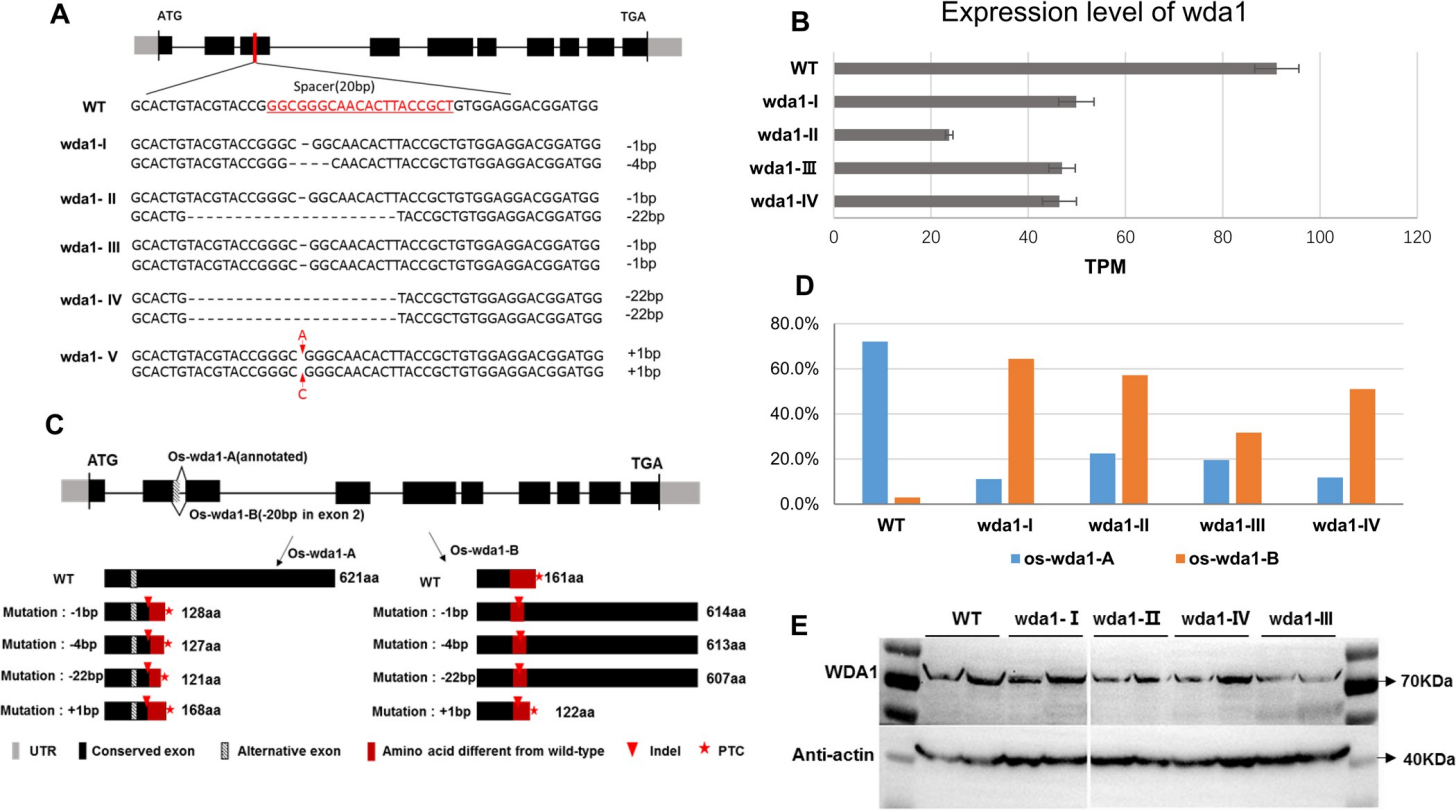
Genotype and alternative splicing isoforms in WT and mutants of *wda1*. (**A**) Gene structure of *wda1* and five different mutant genotypes. Mutants of *wda1-I*, *II*, *III*, *IV* cause a 3n+1-base deletion (n = 0 or a positive integer), *wda1-V* causes a one-base insertion (equivalent to a 3n+2 deletion). The grey box represents UTR, the black box represents CDS exon, the black line represents intron. (**B**) Expression of *wda1* in WT and mutants. The X axis represents the sum TPM value of gene expression of all splice forms. Error bar stands for SEM.(**C**) Two main splice forms of *wda1* gene and the predicted effect of splice events on protein sequences. Os-*wda1*-A means a transcript of the form specified by the canonical annotation, Os-*wda1*-B is the new transcript that we detected with 20 bp deletion in exon2 compared with Os-*wda1*-A. Mutants with 3n+1-base deletion (-1 bp, -4 bp, -22 bp) are expected to cause both premature termination and incorrect framing post the indel if splicing is in the transcript Os-*wda1*-A mode, while a nearly full “wild-type” transcript will be expressed under the transcript Os-*wda1*-B splicing mode. Mutant with 3n+2-base deletion (+1 bp) is expected to cause premature termination under the transcript Os-*wda1*-A splicing mode and also under Os-*wda1*-B mode. The grey box represents UTR, the black box represents conserved exon, the striped box represents an alternative exon region, and the red box represents amino acid sequence different from wild-type. Triangle represent**s** indel, asterisk indicates premature termination codon (PTC). (**D**) Proportion of the two alternative splicing isoforms (Os-*wda1*-A, Os-*wda1*-B) detected by TA cloning. (**E**) Western blots of protein WDA1 in wild-type and mutant homozygotes. The predicted weight of full-length WDA1 protein is 71.2 kDa, anti-actin is 42 kDa. The results showed that through the splicing isoforms of Os-*wda1*-B, nearly full-length protein of WDA1 was produced in the CRISPR knockout lines of *wda1*-*I*, *II*, *III* and *IV*.

Os10g0471100 (*WDA1*, wax deficient anther 1) has LOF homozygous mutants with an easy to screen male sterile phenotype [44]. Using CRISPR/Cas9, we obtained five independent *wda1* indel mutations (Fig 3A) of which four (*wda1- I*, *II*, *III* and *IV*) possessed 3n+1 frameshifts and one (*wda1-V*) had a 3n+2 frameshift. In all five cases, the frameshifts are predicted from the canonical sequence to have catastrophic LOF truncated proteins and the male sterility phenotype [44]. While the wild-type protein length is 621AA (amino acids), the CRISPR frameshifted lines should have experienced PTCs in exon 2 and truncated proteins of length 121 to 128AA. They should also be different in translation post any frameshift and lack the critical WAX2 C-terminal domain (S3 Fig).

**Fig 4:**
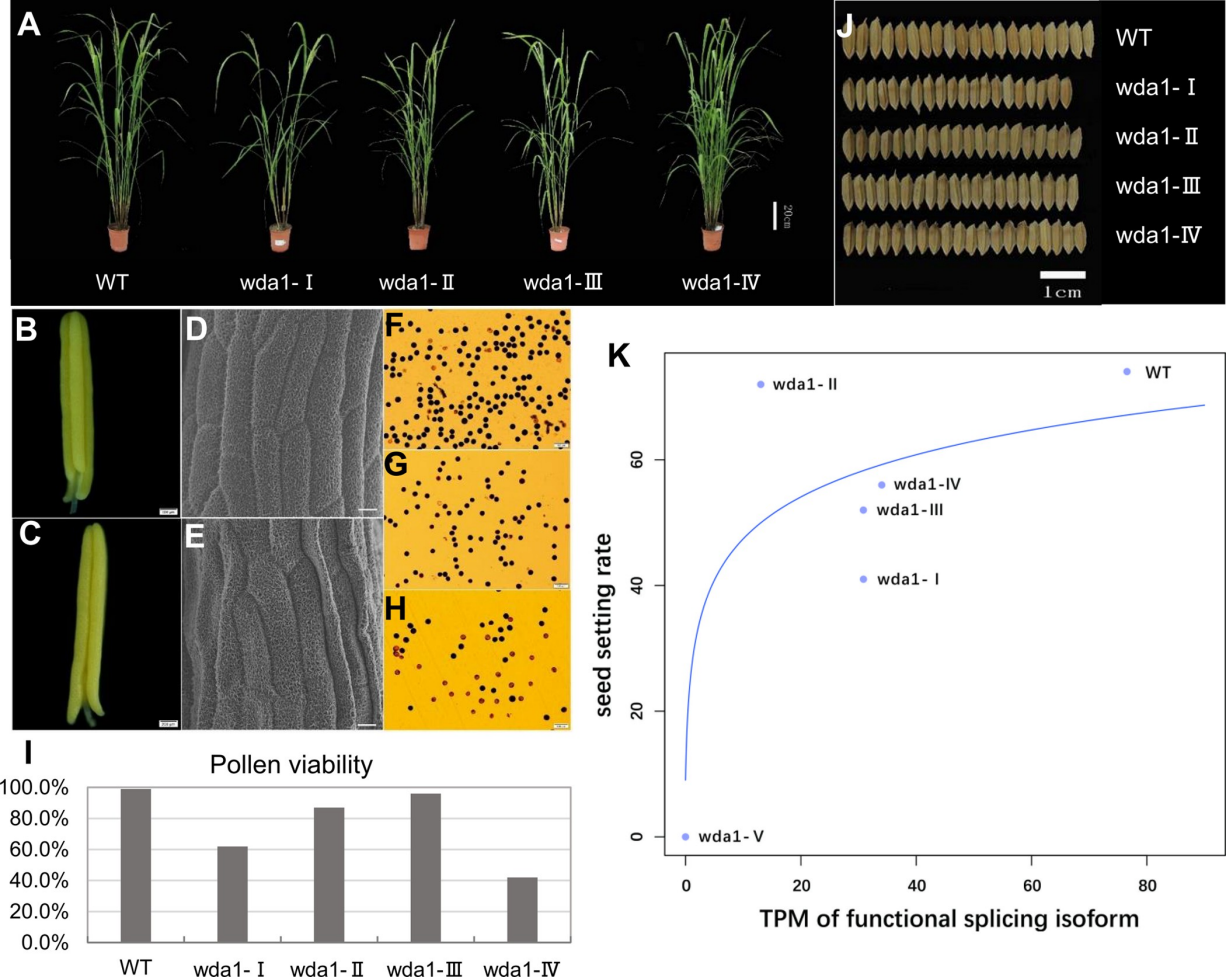
Phenotypic comparison between wild-type and *wda1* mutant plants. **(A)** Phenotypic comparison of wild-type and mutant plants at heading stage. Scale bars, 20cm.**(B)** Morphology of anthers observed by optical microscope in WT. Scale bars, 200μm.**(C)** Morphology of anthers observed by optical microscope in mutant of *wda1-I*. Mutants of *wda1- II*, *III* and *IV* have similar anthers to *wda1-I*. Scale bars, 200μm. **(D)** SEM analysis of the exine characteristics of mature anthers of WT. Scale bars, 10μm. **(E)** SEM analysis of the exine characteristics of mature anthers of mutant of *wda1-I*. Mutants of *wda1- II*, *III* and *IV* have similar exine characteristics to *wda1-I*. Scale bars, 10μm. **(F)** I_2_-KI staining of pollen grains for WT. **(G-H)** I_2_-KI staining of pollen grains for mutant of *wda1-I*. Mutants of *wda1- II*, *III* and *IV* have similar staining pattern to *wda1-I*. Scale bars, 100μm. **(I)** Pollen viability of mutants of *wda1-I*, *II*, *III* and *IV* observed by I_2_-KI staining. Pollen grains that were round and stained black by I_2_-KI were considered viable, and those stained yellow or light red were judged as sterile. The percentage of pollen viability was calculated as viable pollen relative to total pollen counted **(J)** Seed morphology of wild-type and mutants. Scale bars, 1cm. **(K)** The relation between the expression level of the splicing isoform (in TPM) that produces functional protein and fertility. The TPM of Os-*wda1*-A in WT and the TPM of Os-*wda1*-B in *wda1-I*, *II*, *III* and *IV* were used. For *wda1*-*V* we assumed it could be set at the origin. The blue line is the fit of y = f(log(x+0.1)), r^2^ = 76%, *P* = 0.02393. Each genotype has three replicates.

The one 3n+2 frameshift (*wda1-V* line) was indeed sterile and could not be studied further (it was not possible to measure expression or protein size). By contrast, despite the absence of a wild-type allele, all four 3n+1 frameshift mutants (*wda1- I*, *II*, *III* and *IV*) were able to express the gene (Fig 3B), produce near wild-type length transcripts of between 607 and 614AA (Fig 3C), and express protein containing the critical WAX2 C-terminal domain (Fig 3E). These exhibit a seed setting rate of more than 40%, which compares to 70% in the wild-type (Fig 4K). One mutant line, *wda1-II*, has near wild-type fitness levels (Fig 4).

To determine the nature of the alternative splice junctions in this gene, we used TA cloning, and confirmed by RNA-seq and RT-PCR-seq. TA cloning revealed that there are two main AS isoforms for the *wda1* gene. One is consistent with the annotated form, denoted here as Os-*wda1*-A, while the other transcript, Os-*wda1*-B, has a non-canonical 5’ splice site in the canonical second intron (Fig 3C). As this removes 20 bp of sequence its usage also forces a frameshift on all sequence 3’ of its inclusion. In the wild-type rice, the Os-*wda1*-A exists as a major isoform with a proportion as high as 72.1%, while Os-*wda1*-B only accounts for 2.9%. By contrast, in the four 3n+1 frameshift CRISPR mutants, the relative proportions of the two splice form modes altered dramatically (Fig 3D), observed both by RNA-seq and with RT-PCR-seq (S4 Fig). At the extreme there is about 10 times as much of the Os-*wda1*-B form as the Os-*wda1*-A form in these mutants.

This effect on relative proportions of the Os-*wda1*-A and Os-*wda1*-B splice forms may be accounted for by nonsense mediated decay (acting on the A form with the indel or the B form in the absence of the indel) or by the indels inducing more of the B form, or a combination of both. We tested for an effect of NMD by considering what happens when the NMD pathway is knocked down (Table 1). The relative ratio of rescue junction of *WDA1* (chr10:17453792–17453945) is 8/32 = 25% in miUPF1 mutant and 3/31 = 9.7% in miUPF3 mutant compared to 1/29 = 3.4% in WT callus (Table 1). Therefore, the relative ratio of rescue junction of *WDA1* increased by 2.8~7.4-fold in NMD-KD forms. This indicates that some of the increased relative level of the rescue isoform may be attributed to NMD’s activity in the wild type condition removing the B form. The fold-change associated with the indel’s presence is much higher (37.7/1.8~56.6/1.8 = 20.9~31.4-fold) potentially consistent with the indel inducing more of the rescue isoform. However, as the wild type isoform is potentially associated with NMD when bearing the indel we cannot be sure of this.

No matter what the mechanism, as we expect the relationship between dose and phenotype to follow the law of diminishing returns [45] we consider seed set predicted by log of TPM, assuming *wda1-V* to have zero on both measures. The fit of seed set as a function of log (TPM+0.1) is significant (*P* = 0.02393: Fig 4K)).

#### Functional compensation by exon skipping in *bc10*

**Fig 5:**
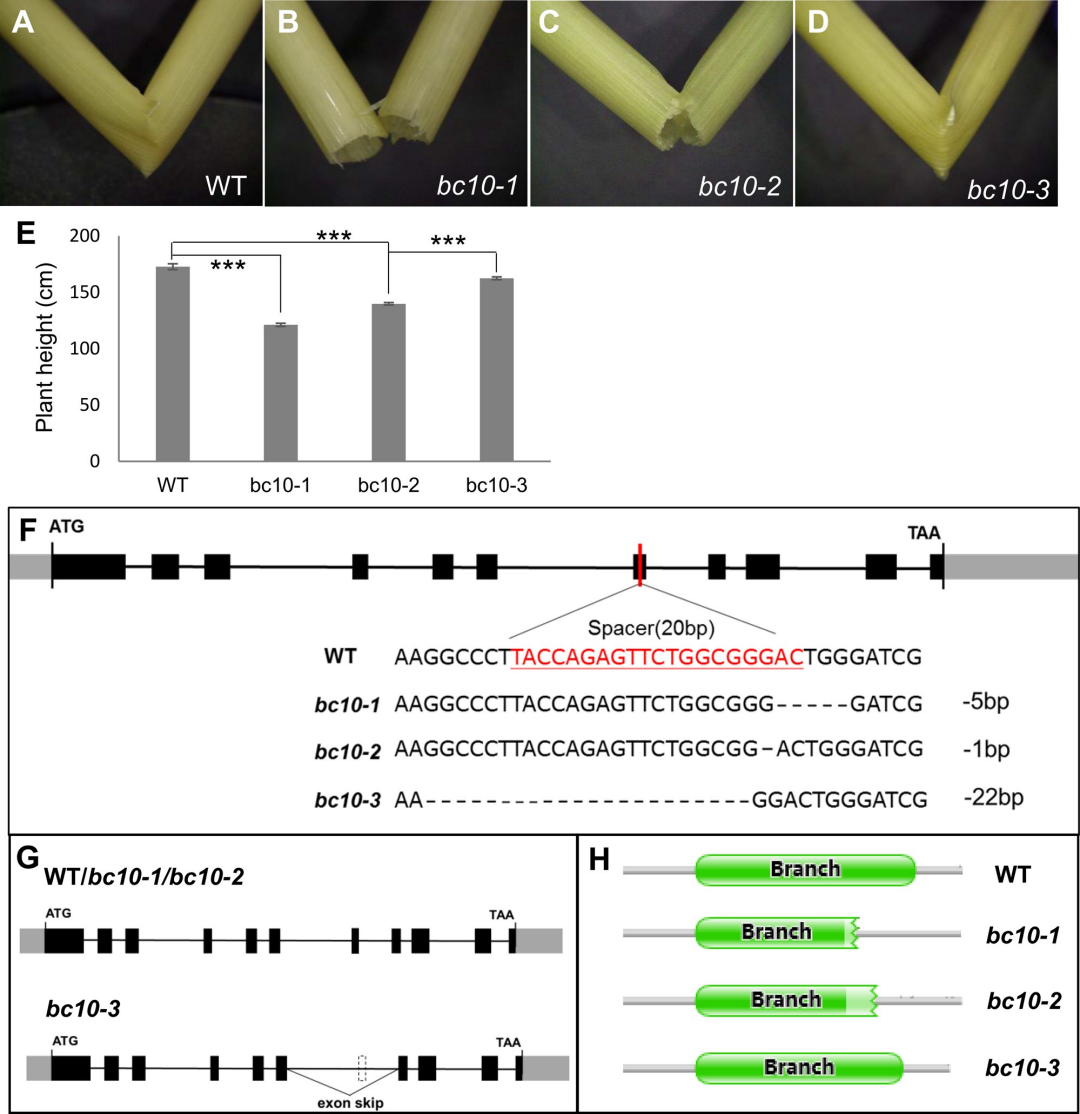
Phenotype, genotype and transcript analysis in wild type and *bc10* mutants. **(A-D) Compared with the wild type, the stem of *bc10-1* and *bc10-2* were** easily broken, while *bc10-3* was not. (E) Plant height of wild type and mutants. Sample size was N = 6, 24, 22, 29 or WT, *bc10-1*, *bc10-2*, *bc10-3* respectively (F) Genotypes of wild type and mutants. (G) The major transcripts found in WT/*bc10-1*/*bc10-2* and *bc10-3*. Number of positive monoclones sequenced through TA cloning: WT (23), *bc10-1*(22), *bc10-2*(44) and *bc10-3*(50). Note that the skipped exon in bc10-3 is a multiple of three long hence frame preserving on skipping. (H) Prediction of BC10 protein domain by the Pfam. Error bar: mean +/- sem. Significance tests: two tailed t-test, *P ≤ 0.05, **P ≤ 0.005, ***P ≤0.001.

The second exemplar considers the rescue of mutants of *bc10*. The brittle culm gene *BC10* (Os05g0170000) is involved in the production of cell wall polymers in rice [46,47]. Reported mutants exhibit retarded growth and brittle culm and leaves after booting stage [47]. To examine this instance, we generated indel mutants. Of the three independent non-3n indel *bc10* mutants with homozygous deletions observed in current study, *bc10-1* has a five-base deletion (-5 bp), *bc10-2* has a one-base deletion (-1 bp) and *bc10-3* has a 22-base deletion (-22 bp) (Fig 5F).

Consistent with the prior reports [47], the stems of *bc10-1* and *bc10-2* are significantly more brittle than those of the wild type. However, the *bc10-3* showed a phenotype similar to the wild-type being not easily broken (Fig 5A–5D). In addition, the plant height of *bc10-1* and *bc10-2* was significantly lower than that of the wild-type, while the *bc10-3* was just 10cm shorter than the wild-type and significantly taller than *bc10-1* and *bc10-2* (taller by 22.1~41.3cm, *P* < 3.51 x 10^-18^) (Fig 5E). That the growth of *bc10-3* is less affected by the mutation than *bc10-1* and *bc10-2* is consistent with *bc10-3* having at least some partial degree of rescue.

The phenotypic difference in *bc10-3* we suspected to be owing to its rescue junction chr05:4196904–4198079, which was detected at high-frequency in *bc10-3* but rarely in wild type (junction reads/junction region depth: 0.002%) by RT-PCR-seq. To further confirm the role of alternative splicing, we collected the cDNA from three mutants as well as wild-type, using TA cloning to capture their transcript isoforms. This revealed that the major transcripts of *bc10-1* and *bc10-2* were consistent with the annotated form of the wild-type and hence disrupted by the non-3n indel mutations. By contrast, in *bc10-3* two types of splicing isoforms were found. The major one is highly abundant (ratio of frame restoring isoform to total sequenced by TA cloning: 49/50). This skips the disruption (Fig 5G) while the other is consistent with the splice form that dominates in *bc10-1* and *bc10-2*(ratio: 1/50).

Prediction of the protein domain of wild type and *bc10* mutants by Pfam, shows a complete core-2/I-Branching enzyme domain in wild-type, a destroyed and incomplete domain in *bc10-1* and *bc10-2*, and a slightly shortened but still intact domain in *bc10-3* (Fig 5H). The exon skipping in *bc10-3* enabling it to have an intact domain is likely to explain why the *bc10-3* is able to maintain stem flexibility, while *bc10-1* and *bc10-2* were brittle. Given that the frame restroing isoform was very rare (barely detectable) in the absence of the indel, this is a strong candidate for indel induced rescue.

The exact mechanism by which the indel in *bc10-3* causes such a large change in the rate of exon skipping is unresolved but may be related to ablation of near end of exon splicing motifs. All of the indels are located in exon 7 (of all 11 exons) that is only 45 bp long (the gene has an average exon length of 151 bp). The non-disruptive mutations are both small and quite central to the exon, where splicing information tends not to reside. *bc10-1* has a five-base deletion, which is 14 bp and 26 bp from 5’ and 3’end of exon 7. *bc10-2* has a one-base deletion, which is 17 bp and 27 bp from 5’ and 3’end of exon 7. *bc10-3* by contrast has a 22-base deletion, which is 17 bp and just 6 bp from 5’ and 3’end of exon 7. We speculate that the indel disrupted splice motifs at the 3’end of exon 7 resulting in exon skipping.

### Most genes have frame restoring isoforms but few are high abudance

Above we identified that many putative rescue forms have the dosage of the rescue form increased in the presence of the indel. Our top candidates for rescue seem to have raised dosage that is important for achieving phenotypic or fitness rescue. This may be owing to indel induction of the novel splice form or release from NMD suppression. Conversely, we might suspect that instances where isoform abundance is not affected by the indel (by whatever mechanism) are unlikely to provide much rescue and hence little variation in fitness. For this special class we need not, however, restrict our analysis to the cases with CRISPR-induced indels as the indels do not affect expression levels. Rather we can consider the issue pan-genomically by analysis of extant transcriptomic data. Here then we further consider such instances using wild type transcriptomics asking what proportion of genes might have a possible rescue isoform (should a non-3n indel hit) and how often the frame-restoring forms might be at dosage potentially compatible with some degree of phenotypic rescue (assuming its dosage is not affected by the indel’s presence). Notice that while in the real data sets many transcripts are likely to have reduced dosage owing to NMD, we are playing the game of asking what if some are not so affected and so would not have dosage modified when restoring the frame of a non 3n indel.

#### The majority of genes have frame shifting alternative splice forms

Based on our definition of splice junctions (S5 Fig), the Nipponbare reference genome [48] (IRGSP version 1.0) contains 125,178 splice junctions (Table 2), which is equivalent to an average of 3.31 junctions per annotated gene (range of 0 to 65). Our genome wide inventory of 1.2 Tb of RNA-seq data from 16 rice lines (S7 Table) reveals 31,363 genes (82.8%) with multiple exons, including 29,728 genes (78.5% of all genes, 94.8% of genes with multiple exons) with multiple splicing isoforms. These new data indicate 518,074 splice junctions in annotated genes, which is 4.1-fold higher than the reference annotated data (Table 2) and indicates 14.0 junctions per gene (range of 0 to 906), of which 402,230 were previously unannotated. For a breakdown of the various modes of non-canonical splicing see S8 Table. Suggestive that many may be splicing noise, of the non-canonical (i.e. previously unannotated) splicing junctions only 209,407 (52.1%) are found in two or more of the sampled genomes (S9 Table).

**Table 2:** Genome-wide view of splice junctions in rice in Genome annotation versus RNA-seq Dataset.

		Genes with Junctions		Genes without	Alt. Spliced	Total Gene
		Junction No.	Genes No.	Junction	Gene No.	No.
IRGSP Genome Annotation^a^		125,178	26,558	11,300	4,014	37,858
RNA-seq Dataset	Overlap with annotation	115,844	24,535	5,600	4,007	30,135
	Non-canonical junctions^b^	402,230	29,699	-	29,699	29,699
	Total^c^	518,074	31,363	5,600	29,728	36,963^d^
	non-3n junctions	255,971	27,458	-	-	-

^a^ IRGSP Nipponbare sequence Version 1.0.

^b^ See S5 Fig.

^c^ 176,567 splice junctions were detected outside all known annotated genes and are not included in the totals.

^d^ No supporting RNA-seq reads detected for the remaining 895 genes.

Predicting the possible impact of the non-canonical junctions on annotated genes, we find that 255,971 (63.6%) would introduce non-3n events (Table 2) and therefore cause transcriptional frameshifts (which could potentially rescue some non-3n indels). For the 209,407 seen in 2 or more genomes the comparable figure is 65.4%. Both are very close to null expectation from random noisy splicing of 2/3 that would change the frame. Owing to the large samples these numbers are nonetheless very weakly but highly significantly lower than expected (Chi-squared = 1,660, *df* = 1, *P* < 2.2 x 10^−16^ for all; Chi-squared = 151, *df* = 1, *P* < 2.2 x 10^−16^ for the repeatable ones). This proximity to null expectations is perhaps surprising as we should expect a great excess of 3n junctions if NMD is removing from view some isoforms that change the frame. We conclude that most genes have frame rescuing isoforms at some expression level. We find no evidence for a large degree of tissue specificity of non-canonical forms (S1 Text).

#### Many frame shifting isoforms are rare transcripts

The above result demonstrates that we cannot exclude native alternative isoforms as a means of rescue solely on the basis of the absence of frame restoration isoforms. With increased sequencing depth we may, however, just be picking up rare isoforms with no prospect of full rescue and that contribute minimally to fitness variation between indels.

To determine the relationship between sequencing depth and junction detection power [49], we first compared junctions detected in the RNA-seq dataset to their genome coverage and found when the sequencing data is greater than 10GB (i.e. the transcriptome coverage is greater than 170.4x), the number of junctions detected tends to stabilize (S6 Fig). This means that the large database that we employed would provide such robust detection power that it is prone to finding rare noisy splice forms. To cross-check the validity of these novel splice junctions [50], we used RT-PCR-seq to amplify transcript variants in 20 arbitrarily selected gene loci in Nipponbare (S10 Table). Our RT-PCR-seq amplicons detected 286 junctions in these 20 genes, indicating an average of 14.3 junctions per gene (S11 Table). From RT-PCR-seq we matched 94 of the 96 annotated splice junctions in the Nipponbare reference genome (Table 3) which compares with 93 of 96 being matched by RNA-seq. The two unmatched by RT-PCR-seq were two of the three not identified by RNA-seq. Assuming these two are mis-annotations, then RNA-seq has a 93/94 = 99% accuracy. This supports the notion that splice junctions identified via RT-PCR-seq are a fair reflection of *bona fide* common splice junctions.

**Table 3:** Cross-validation of spliced junctions using RT-PCR-seq amplification.

	Annotation^a^	RNA-seq^b^	RT-PCR-seq^c^	Shared between RNA-seq and RT-PCR-seq
Annotated junctions	96	93	94	93
Non-canonical junctions	-	225	192	77
Total	96	318	286	170
non-3n junctions (% in non-canonical junctions)	-	139(61.8%)	116 (60.4%)	49(63.6%)

Data for twenty genes randomly selected from the rice genome.

a, annotated IRGSP version 1.0 data.

b, downloaded RNA-seq data for Nipponbare.

c, data of RT-PCR-seq amplification with subsequent Illumina sequencing from Nipponbare.

The above describes the ability to identify canonical junctions. More generally, we identified by RT-PCR-seq only 170 (53.5%) of the net 318 splice variants seen in the RNA-seq data (Table 3). In principle RT-PCR-seq, being targeted, should be the more sensitive technique. Why then does it detect such a low proportion? With the above 99% accuracy for annotated splice junctions, this is almost entirely owing to a relatively low capture of RNA-seq derived non-canonical splice variants by RT-PCR-seq analysis (RT-PCR-seq confirms only 77 of 225 RNA-seq novel junctions = 34%). One reason for this is sampling artefact: more samples are used in RNA-seq (multiple tissues from multiple different stages) than in RT-PCR-seq (1 tissue of 1 stage, shoot of ~14 days). For a fairer comparison, if we only use RNA-seq for shoot samples (three biological replicates of a shoot sample were used here), we identified 37 novel junctions and 93 annotated junctions in the 20 selected genes. Of the 130 (37 + 93) splice variants seen in the RNA-seq data, 117 (90%) were identified by RT-PCR-seq. RT-PCR-seq confirms 24 of the 37 non canonical junctions = 64.9%, double the proportion previously observed. The unreplicated junctions remain a mystery but could be due to different stages/samples used or some isoforms failing to be amplified in RT-PCR-seq.

The above suggest a richness of noisy rare splice forms that vary sample to sample. Consistent with such a possibility, the frame of novel splice boundaries is not affected by method: among the 192 novel junctions suggested from RT-PCR-seq, 116 (60.4%) would introduce non-3n events, which is similar to the RNA-seq data value of 139 of 225 (61.8%) (Table 3) (Chi-squared = 0.03, *df* = 1, *P* = 0.85). Neither of these are different from a null of 2/3:1/3 expected were these junctions just random noisy events (RT-PCR-seq, Chi-squared = 3.38, df = 1, *P* = 0.066; RNA-seq: Chi-squared = 2.42, df = 1, *P* = 0.12). The near concordance with 2/3:1/3 ratio is similarly supportive, as is the fact that (gold standard) RT-PCR-seq identifies 192 novel junctions of which only 77 were also seen by RNA-seq. Thus the most parsimonious model is that the deeper we sequence the more we find novel splice junctions, most of which are likely real (not analysis artefacts) but spurious splice forms [51,52] that are as a consequence seen rarely and not in an obviously deterministic manner.

Such a model predicts that replicability should increase if minimum threshold for reads to support a splice form also increases. In agreement, the low replicate proportion is mainly due to low concordance in junctions with few supporting reads (putative low frequency junctions). If we require at least 10 reads to detect a reliable junction in both datasets, we find 241 and 151 junctions in RT-PCR-seq and downloaded RNA-seq, respectively, with 127 shared (84% replicated in downloaded). More generally, as the minimum read threshold increases so the degree of agreement increases in an asymptotic manner (S7 Fig). Hence the discordancy is largely owing to rarely employed junctions detectable with ever deeper sequencing.

Assuming the plethora of possible rescue splice forms reflects a richness of rare isoforms, we can also ask, as we did for the indel-associated isoforms (S2A Fig), what proportion of genes have a non-canonical isoform if we consider the asymptote of the function relating minimum read depth to proportion of genes with a non-canonical isoform in the wild type. For RNA-Seq data this asymptote is under 10% (S2B Fig). However, even if the putative rescue form has read-depth ≥ 500, it may simply be because the sequencing depth is ultra-high (it can be ≥ 10,000), so the absolute expression level of these forms could still be very low. However, we can assume that any splice form that is a high (>10%) frequency compared to the canonical form would be able to provide some degree of fitness increment (all other things being equal). To address the problem of the relative level we estimated the non-canonical/canonical ratios by considering “non-canonical read-depth” / “canonical read-depth”. A value of 1 implies the non-canonical and canonical junctions are used equally. From RNA-Seq data of 37,858 genes, 28,341 genes have non-canonical junctions, 15,388 genes with the “non-canonical read-depth” / “canonical read-depth” >0.1, 9,238 genes with the “non-canonical read-depth” / “canonical read-depth” >0.5.

By no means will all these non-canonical junctions enable rescue of any kind and, of those that could enable frame restoration, for any given indel, only some of these will potentially provide rescue, either because the frame or the protein function is not restored. However, we notice that the above RNA-seq based calculations of the proportion of non-canonical junctions at appreciable levels (>0.5 relative proportion) is much higher (9238/37858 = 24%) than we estimated by RT-PCR-seq for the rescue junctions seen after CRISPR indel induction (this goes to 33% if we restrict analysis to genes with non-canonical splice forms). Why might this be? An issue here is that the “non-canonical junctions” are not simply equivalent to the “rescue junctions”. For RNA-seq, the “non-canonical read-depth” is taken as the junction (after excluding those standard annotated junctions) with maximum read-depth. However, the “rescue junctions” in CRISPR is not always the “non-canonical junction” with the highest read-depth. When we use the same “non-canonical depth/canonical depth” for CRISPR mutants we indeed see that these numbers are comparable to the RNA-seq data (S12 Table). For “rescue junction depth / canonical depth”, the proportions are lower–i.e., the rescue junction are not always the most abundant non-canonical junction as they are defined in terms of a junction that could rescue frame given a certain indel. Thus the RNA-seq data does not suggest that rescue junctions may be common much of the time. We conclude that use of non-canonical splice junctions in which the indel does not affect the isoform levels, are unlikely to contribute much to between-indel variation as for the most part frame restoring isoforms are rare transcripts.

### Evidence that indel associated non-canonical splicing might lead to between strain diffrences

Above we have considered the immediate impact of indels and whether they might have their fitness effects modulated by non-canonical splicing. We find that induction by the indels of frame restoring forms is probably common. Might this in turn possibly mediate routes to novel modes of gene body evolution? We thus ask whether such events can lead to between-strain differences. To search for evidence relevant to this possibility we examined NBS-LRR genes which are notable for their high rates of polymorphism both in rice and in *Arabidopsis*, including a high frequency of LOF events that occur even between closely related varieties [53,54].

With such a large gene family the knockout status of NBS-LRR genes is hard to experimentally define. Nonetheless, being parasite-resistance genes we expect them to be conditional essentials at most, conditional on the infecting pathogen strain. Thus we do not consider this an exemplar of phenotypic rescue so much a novel mode of evolution, associated with non-canonical isoform usage.

**Fig 6:**
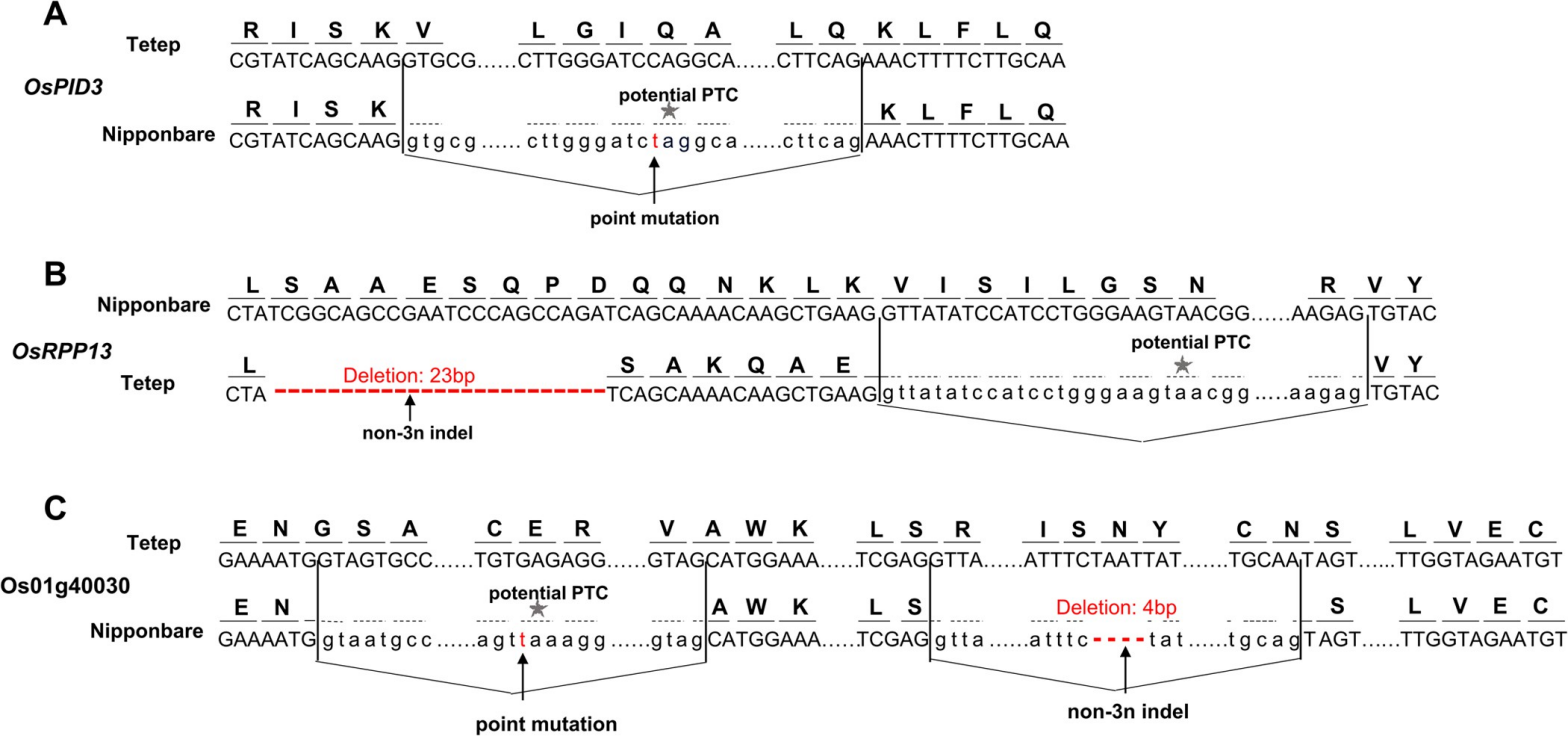
Natural alternative splicing responses to mutation between NBS-LRR homologous pairs in Nipponbare and Tetep genomes of rice. **(A)** OsPID3 showing canonical form (chr06.fgenesh1561, Tetep) where Nipponbare (LOC_Os06g22460) has a point mutation which would cause a premature termination codon (PTC) but is rescued by an alternative splicing junction. In tetep, no splicing happened around the location of the PTC. **(B)** OsRPP13 showing canonical form (LOC_Os08g14830, Nipponbare) where Tetep (tig00011639.fgenesh73) has non-3n deletion of 23 bp which would cause a PTC but is rescued by an alternative splicing junction. **(C)** Os01g40030 showing canonical form (chr01.fgenesh1832, Tetep) where Nipponbare (LOC_Os01g40030) has point mutation and non-3n indel which would cause PTC but is rescued by two alternative splicing junctions. Solid line and dashed line above codon indicate the actual amino acid sequence and the amino acid sequence under the hypothetical splicing pattern found in the homolog, respectively. Asterisks indicate hypothetical premature termination codons. Flexed line indicates splice junction. Red letters indicate differences between homologs.

Given the close resemblance between NAS and the various indel associated modes that we consider here, we searched for both putative homozygous frameshift-inducing nucleotide polymorphisms and nonsense mutations generating PTCs, between Tetep, a highly resistant variety, and the model line, Nipponbare (Fig 6). A putative frameshift mutation was called if the A-nip gene is spliced according to the pattern of the A-tetep gene. N.B. we see only one splicing isoform in the Nipponbare, possibly owing to the low coverage, while in the Tetep two isoforms are seen in 7:109 ratios (splicing as Fig 6B-tetep VS as Fig 6B-Nip). Cause and effect here are not transparent as the mutation could occur but subsequently be rescued by a splicing change which then becomes fixed within the relevant line or, alternatively, a fixed splicing change could enable accumulation of mutations that would otherwise have been damaging.

Of 35 orthologous pairs of NBS-LRR genes examined (S13 Table), 17 exhibit some form of alternative splicing that avoids a PTC. Of these 17 genes, four skip the exon with the hypothetical PTC induced by point mutations, as in the *OsPID3* gene [55] (Fig 6A). This is potentially consistent with nonsense-associated alternative splicing [43], a process in which nonsense mutations are associated with, or directly cause, a change in the splicing pattern of the exons containing them, rather than being rescued by frameshift splicing events (which couldn’t rescue point mutations generating PTCs). Comparably, ten skip a hypothetical PTC caused by a 5’ non-3n indel. In this case the PTC is again within the exonic sequence that is skipped, but the exon skipping is also needed to restore the correct reading frame, as in the *OsRPP13* gene (Fig 6B). Three contain both point and frameshift mutations as in *Os01g40030* (Fig 6C). Here one alternative splicing event removes the exon containing the PTC and another event removes from CDS the indel induced frameshift. The splicing provides consistency of protein sequences between homologous genes and avoids premature termination.

### Functional compensation of point mutations in rice populations

Is use of non-canonical isoforms a means to explain variation is fitness of mutations other than non-3n indels? We sought to test whether a novel junction could rescue other types of mutations, notably point mutations. We indeed found such cases in genes likely to participate in domestication or sub-speciation between *indica* and *japonica* rice.

**Fig 7:**
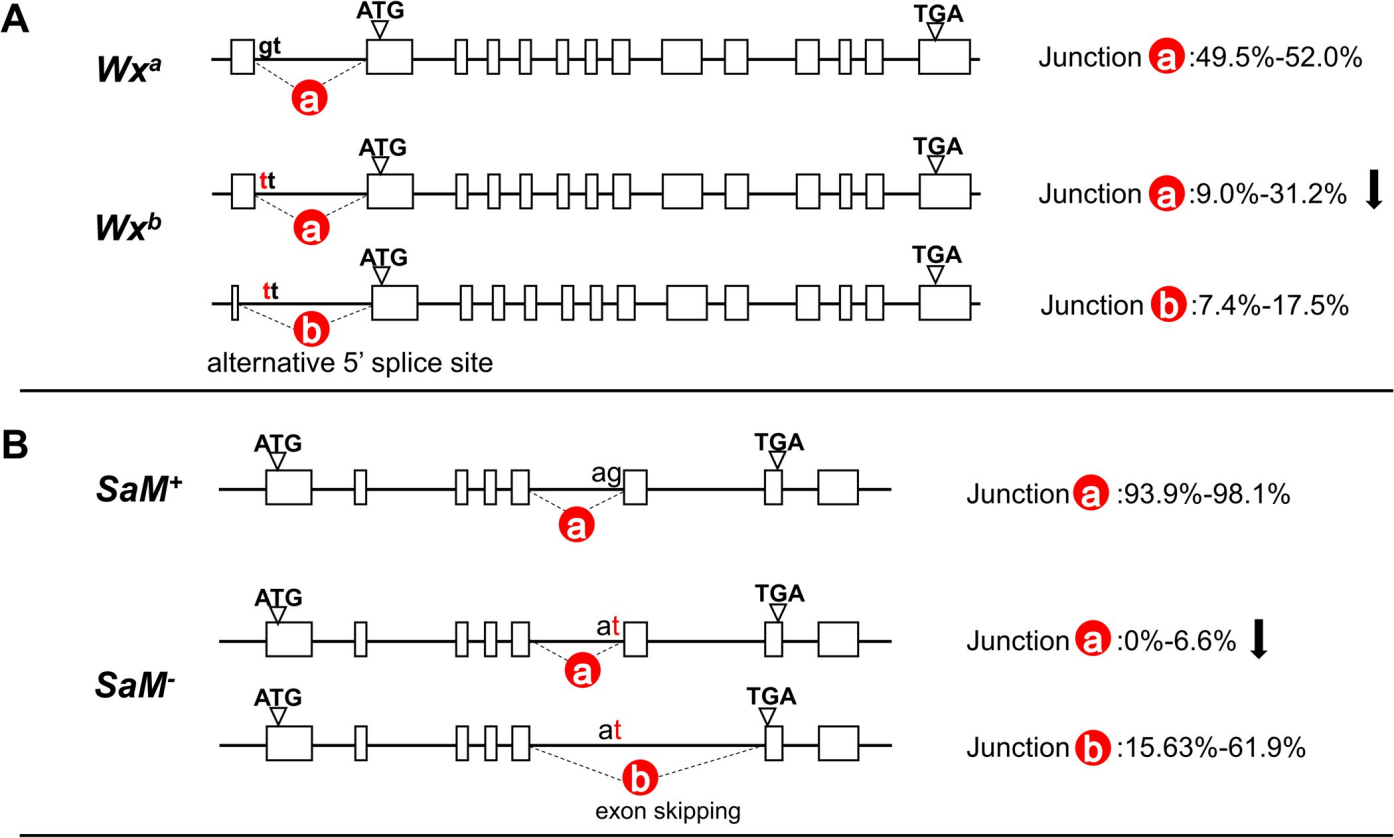
Splicing- mediated rescue in alleles with point mutations in rice. RNA-seq data were collected from 55 downloaded datasets and 2 local sequenced O. *rufipogon* samples (SRR14163371, SRR14163372). The RNA-seq data were processed with the same pipeline described in the paper to obtain the splicing junctions of different (A) *Waxy* and (B) *SaM* alleles. The proportions of different splicing junctions were calculated as (junction reads/junction region depth). Dashed lines represent differentially employed junctions. The major splice form in *Wx*^*a*^ involves Junction-a with Junction-b not being detected. *SaM* Junction-b is also not detected in *SaM* ^+^. In addition to the two splice forms with junction-a and junction-b (the sum of them is not 100%), another major form of splicing is intron 5 retention. The position of each junction: *Waxy-* Junction-a: chr06:1765760–1766889; *Waxy-* Junction-b: chr06:1765667–1766889; *SaM*–Junction-a: chr01:22380909–22381236; *SaM*–Junction-b: chr01:22380909–22382464.

For example, the *waxy* gene is responsible for amylose content, and has possibly been selected during domestication of *O*. *sativa japonica* rice [56,57]. Two alleles have been reported for this gene: *Wx*^a^ found in *O*. *rufipogon* and *O*. *sativa indica* has GT at the 5’ splice site of the first intron in the 5’ UTR, while *Wx*^b^ found in *O*. *sativa japonica* has TT at this 5’ splice site, rendering splicing less efficient. Commensurate with efficient splicing enabling higher levels of transcripts [58,59], in *rufipogon* and *indica* the *Wx*^a^ gene exhibits a high level of expression, in contrast to the expression level of *Wx*^b^ in *japonica* which is lower [57]. This splice site, junction-a (chr06:1765760–1766889), is considered to be the ancestral form. Compared with *Wx*^*a*^, the mutation G→T in *Wx*^*b*^ at this splice site, both reduces usage of junction-a and leads to an alternative 5’ splice site in the 5’ UTR intron (Fig 7A). The relative proportion of transcripts employing junction-a is substantially decreased from 49.5% - 52.0% in *Wx*^*a*^ to 9.0%-31.2% in *Wx*^*b*^ (Fig 7A). The expression of the other splice form with alternative 5’ splice site (Junction-b: chr06:1765667–1766889) that detected in *Wx*^*b*^ not *Wx*^*a*^, partly rescues the decreased expression level of the ancestral form (Fig 7A). The outcome is a high amylose content in *rufipogon* and *indica* with a functional splicing isoform (with junction-a), but an intermediate amylose content in *japonica* with inefficient splicing from junction-a and a rescuing isoform employing junction-b (Fig 7A).

A second case is from the gene *SaM*, which is associated with *indica-japonica* hybrid male sterility [60]. The *SaM*^*+*^ allele in *indica* has AG at the 3’ splice site of the fifth intron while the *SaM*^*-*^ allele in *japonica* has AT. The splicing isoform of *SaM*^*+*^ with junction-a is supposed to be the ancestral form. The mutation G→T (from *SaM*^*+*^ to *SaM*^*-*^) causes a severe reduction of the relative proportion of the ancestral isoform, from 98.1% to 6.6% in *SaM*^-^(Fig 7B). The splicing isoform with junction-b (sixth exon being skipped) in *SaM*^-^ generates shortened but functional proteins. The shortened protein from *SaM*^-^ is required for selective interaction between *SaM* and *SaF*, leading to allele-specific gamete elimination.

These examples show that novel junctions could compensate for mutations that impair gene function or transcription and that the effect is not limited to non-3n frameshifts. This further supports the claim that non-canonical splicing can be an important mode of mutation buffering but we cannot be sure that the effect is as immediate as seen with our induced indels and may instead reflect multistage evolution.

## Discussion

The finding that usage of non-canonical splice forms can sometimes enable some degree of rescue of what should have been disabling mutations provides a solution to the non-essentiality paradox: why seemingly disruptive mutations aren’t as disruptive as they might have been predicted to be [18–20]. In both of our detailed examples we also witnessed that not all non-3n indels are equivalent, even if in the same exon. In *wda1*, the mechanism permits rescue of 3n+1 indels alone, while in *bc10*, only a large indel was associated with rescue. This in turn suggests that a simple map from genotype to phenotype to fitness is unlikely to capture all the complexity of mutational impact [20]. As previously noted [18–20], such complexity also complicates interpretation of CRISPR editing, especially if this can even induce exon skipping [7,19–23,26,61,62]. For implementation of non-3n indel screens, investigation of more than one indel in the same gene, preferably both 3n+1 and 3n+2, thus seems advisable. Indeed, had we only the evidence from the 3n +1 indels we would have concluded that *WDA1* was not an essential gene. That we also find evidence that non-indel mutations can be compensated (e.g. restored amylose levels owing to recovered splice forms) is consistent with a broader relevance for non-canonical splicing.

For all the above cases, RNA level analysis to explore instances in which little phenotypic disruption is witnessed, especially, when this is unexpected, seems also a sensible recommendation. However, our results strongly underpin the need for this analysis to be done in the organism with the indel, rather than by importation of precompiled transcriptomic resources under the assumption that the indel is passive to the splicing process. Indeed, perhaps our most striking discovery is that the majority of frame restroing isoforms are seen only in the presence of the indel, even when using high resolution RT-PCR. More generally, we expect that full rescue will be rare if the indel does not modify the abundance of the frame restoring form: while potential rescue forms are common, in the sense that most genes have one, few such alternative transcripts are natively at any appreciable titre. This may well be because most passively produced frame shifting isoforms are a consequence of functionless noisy splicing. That most alternative splicing is noisy in this species is evidenced by the rates of usage of the frameshifting junctions which are close to neutral null. The molecular evolutionary properties of exon extensions (being similar in rates to introns), supports the same (see S2 Text).

Here we were not focused on mechanism by which indels modify splice patterns and have little to contribute on this front. However, it appears that indel-associated alternative splicing (IAS) is mostly not the same as (enigmatic) nonsense associated alternative splicing (NAS) as it was originally conceived, i.e. as a mechanism of exon skipping. Beyond this we see no obvious relationship between induced skipping and proximity to exon junctions. We suggest that instances in which indels induce new splice forms could make for instructive resource for the analysis of mechanisms of splicing.

We also observed, however, that indels are associated with modified levels of pre-existing splice forms, *Os-wda1*-B form being a case in point, despite the indel not being in the exon concerned. There are at least two extreme models to account for this: 1) constant proportionality of splice forms but altered transcriptional levels or 2) altered proportion of splice forms and constant level of transcription, both coupled with NMD reducing the indel- Os-*wda1*-A form (one might also conjecture differences in RNA stability rather than transcription rate). As regards the first possibility, one can imagine a feedback circuit in which a low dose of active WDA1 protein causes increased transcription levels of *WDA1* (possibly via negative feedback associated with high dose) so boosting absolute levels of transcription and with it the absolute levels of the normally rare (2.9%, ~2 TPM) splice form. This is not without precedent as for 5% of yeast heterozygous knockouts the protein level is approximately wild-type level suggesting some sort of compensatory negative feedback [34]. An alternative is that the indels themselves (and hence CRISPR indirectly) modulates the relative splicing rates of Os-*wda1*-A and Os-*wda1*-B forms while not affecting the absolute transcriptional rates. Mutations modulating splicing are well described [63], but in this instance the indels would need to modulate the splicing rates of exons 5’ from the exon containing the mutations, not the splicing of the mutation-bearing exon directly.

We do not expect that non-canonical splice-form frame restoration should be phylogenetically or intra-genomically universal. Variation in intron density is likely to be an important variable. High intron density (introns per bp of CDS) predicts both a multitude of alternative splice forms, hence more opportunities for a frame rescuing isoform, and smaller exons, hence less disruption from exon skipping. In addition, with small exons a higher proportion of sequence will be in proximity to exon ends where the majority of splice modifying mutations occur [64]. In principle then we broadly predict that non-canonical splicing modulating fitness should be more accessible to species with small effective population sizes (*N*_e_). Small effective population size is associated with weakened purifying selection [65] which is turn is raised to explain the high intron density associated with low *N*_e_ [66,67] and the high diversity of alternative splice forms [68–70]. Furthermore, within genomes the likelihood of an intron having alternative junctions increases with the size of the intron [71]. As both mean intron size and density increase as effective population size decreases [67,72], we expect that the diversity of alternative splice forms should be negatively correlated with effective population size (mean exon size should positively correlate with *N*_e_), although phenotypic complexity (in the form of the diversity of cell types) is likely to covary [68]. Very many of these splice forms are however, likely to be simply the result of noisy error-prone splicing, associated with low *N*_e_, and not of any direct utility [71,73]. Nonetheless, we expect non-canonical isoform rescue to be more common in multicellular organisms, especially macroscopic ones. That we see it in rice is then to be expected as it is quite intron rich (approximately 5/9 of CDS gene sequence is intron). We similarly would expect it in humans (18% of a gene is coding exon) but probably not in *Saccharomyces* where 95% of genes are intronless.

## Materials and methods

### Genome-wide assessment of alternative splicing

We collected 99 published rice RNA-seq datafiles from NCBI SRA (https://trace.ncbi.nlm.nih.gov/Traces/sra/, retrieved with), a total of 1.2 Tb from 16 rice lines, 10 tissues, and 11 stages. In total, the full dataset contained nine cultivated rice lines, including six *indica*, two *japonica* and one *Oryza glaberrima*, five wild lines (three *Oryza rufipogon*, one *Oryza barthii* and one *Oryza meridionalis*), and two hybrid lines (one *japonica* × *indica*, one *indica* ×*japonica*). To integrate datafiles, we first merged sequences from the same samples with technical duplicates to obtain a total of 55 independent sequence samples (the detail of datasets shown in S7 Table). To assemble our transcript inventory, we first mapped the acquired RNA-seq data to the Nipponbare reference genome (Os-Nipponbare-Reference-IRGSP-1.0, http://rapdb.dna.affrc.go.jp/) using Tophat2 [74] (version 2.1.0) with minimum and maximum size of introns set to 67 and 3608 bp [75,76], to detect splicing junctions which span exon-exon. We also tested StringTie [77] in identifying splice junctions, and found consistent results with Tophat2 (S3 Text and S14 Table). We then used standard annotation (RAP-DB-IRGSP-1.0_representative_2016-03-09.tar.gz, https://rapdb.dna.affrc.go.jp/) for Nipponbare to identify non-canonical splicing junctions (S5 Fig) using custom python scripts (S2 Dataset). A full data processing flow was shown in S8 Fig.

To enable highly quality of annotation we consider exon expansion/contraction, exon skipping and mutually exclusive exons events rather than use of alternative intron retention. We did not analyse intron retention as these cannot be unambiguously resolved (e.g. unannotated exons may erroneously inflate measures).

### PSI of the junctions detected in the downloaded RNA-seq datasets

PSI (Percent-spliced-in) values for all collected datasets were calculated using the Whippet workflow by supplement the annotated as well as novel junctions analyzed in this study (S2 Dataset). Since original PSI was defined for exons, a PSI for a junction here was defined as the proportional abundance of the splicing path containing the differential exon node which induced by the junction [78].

### Cross-validation of spliced junctions using RT-PCR-seq amplification

Shoot samples of Nipponbare (14 days old, 3 independent replications) were collected for total RNA extraction using the MiniBEST Plant RNA Extraction Kit (TaKaRa). RNA samples were reverse transcribed into cDNA libraries using PrimeScript II 1st Strand cDNA Synthesis Kit (TaKaRa), which served as templates for amplification of transcripts of the 20 targeted genes by 32-cycle PCR (primers given in S10 Table). Final PCR amplification products were equivalent mix and sequenced as 150 bp paired-end reads using Illumina HiSeq4000 at BGI, Shenzhen, China. RT-PCR-seq products were processed as described for detecting splicing junctions (S8 Fig).

### CRISPR / Cas9 induced frameshift mutations

We selected 130 genetic loci and manually edited them by CRISPR/Cas9-mediated gene editing system for gene knockout. We assembled Cas9/sgRNA constructs with 20-nt target sequences following a standard approach [79] to produce guide sequences of these 130 focal genes (S1 Table). We then transformed the Cas9/ sgRNA vector (pRGEB31, http://www.addgene.org/51295/) into three rice varieties (Kasalath, TP309 and Wuyunjing24) individually via *Agrobacterium-*mediated transformation of callus [80]. Independent transgenic events were isolated in the presence of hygromycin B.

To assess transformation success, we extracted genomic DNA from leaves using the standard cetyltrimethyl-ammonium bromide (CTAB) method [81]. Fragments containing sgRNA target regions were amplified by PCR and PCR products sequenced by Sanger method. Only successfully edited plants (S1 Table) were used in subsequent experiments. Since rice is diploid with two chromatids, the results of gene editing may appear in three different types: first, heterozygous, if only one of the two sister chromatids was mutagenized at the locus, second, homozygous, if both alleles were mutagenized and breaks were repaired with the same mutations, or third, biallelic, if both alleles were mutagenized but breaks were repaired with different mutations. In some mutants, the mutation occurred in some tissues but not others, resulting in chimeric plants.

### Detecting junctions in mutant rice by RT-PCR-seq amplification

To obtain transcription data of mutants, we extracted total-RNA and reverse transcribed it into cDNA libraries. To avoid failure of amplification due to low expression, each gene was assessed in Genevestigator (https://genevestigator.com/gv/), and tissues with highest expression were selected for RNA extraction. Primers were designed with reference to gene annotation, and transcript sequences of each mutant gene were obtained by RT-PCR-seq amplification (S2 Table). These PCR products were processed with the same procedures to detect splicing junctions as described above. In order to quantify the proportion of junctions in gene expression, we used junction reads/ junction region depth.

### Genotypic and phenotypic assessment of *wda1* lines

Seven transgenic T0 *wda1* mutants with three independent genotypes (*wda1-I*, *-II*, *-V* in Fig 3A) were obtained by CRISPR/Cas9-based editing which targeted the third exon before the two important domains, conveying hydroxylase activity and WAX2 C-terminal activity, respectively (Fig 3A, sgRNA sequence: AGCGGTAAGTGTTGCCCGCC). Mutants of genotype I have biallelic mutation that one DNA double-strand has a one-base deletion (-1 bp) and the other has a four-base deletion (-4 bp) in the third exon of *wda1*, named *wda1-I*. Mutants of genotype II also have biallelic mutation with one-base deletion (-1 bp) in one strand and 22-base deletion (-22 bp) in the other, named *wda1-II*. The mutant with genotype V had a one-base insertion (+1 bp) in each strand, but one had insertion of A and the other had C, so, it was also a biallelic mutant, named *wda1-V*. (Fig 3A). The nucleotide sequences of *WDA1* gene in wild-type Kasalath and *wda1* mutants in this study are available in S3 Dataset.

Phenotypically, only the *wda1-V* mutant had the male-sterile phenotype, whereas mutants *wda1-I* and *wda1-II* were fertile (Fig 3J–3K). Subsequently, from progenies of these self-crossed T_0_s, two additional mutants were detected, including genotype III with homozygous deletions of one-base (-1 bp) and genotype IV with homozygous deletions of 22-base (-22 bp) (Fig 3A). These two mutants named *wda1-III* and *wda1-IV* also had the partially male fertile phenotype (Fig 3J–3K). Moreover, there was no difference during vegetative growth between the mutants and wild-type rice, including plant height, tiller number and flowering time, which indicated that mutant plants can undergo normal vegetative growth.

To examine pollen viability, mature anthers before flowering were placed on glass slides. Anthers were cut up and squashed with coverslip, then stained with 1% w/v iodine/potassium iodide solution (I_2_-KI) for 5 min at room temperature to observe viable and sterile pollen by light microscope. Pollen grains that were round and stained black by I_2_-KI were considered viable, and those stained yellow or light red were judged as sterile. We repeated measurements on three samples, each sample selecting three different fields of view. The percentage of viable pollen was calculated relative to total pollen counted.

To observe anther morphology, we collected fresh spikelets before flowering from wild-type and mutant plant on ice for immediate microscopic observation. Anthers were observed under optical microscope to determine morphology and under a scanning electron microscope (SEM) to determine structure of anther walls.

### Expression analysis of *WDA1* gene

To examine gene expression of *WDA1*, we used Genevestigator (https://genevestigator.com/gv/start/start.jsp) to detect expression in different organs and how it changed at different growth stages across a large number of wild-type rice RNA-seq samples. Expression analysis from Genevestigator showed that *WDA1* is expressed mainly in the floret, and expression increases from booting to flowering stages (S9 Fig).

To test expression as well as transcript isoforms of *WDA1* in wild-type and mutant rice, we extracted total-RNA from spikelet at flowering and sequenced as 150 bp paired-end reads using Illumina HiSeq4000 platform at BGI, Shenzhen, China.

To detect, we used TA cloning to detect separate transcript isoforms of *WDA1*. We obtained the cDNA library of wild-type and mutant rice (*wda1-I*, *wda1-II*, *wda1- III* and *wda1-IV*) acted as template described above, the Taq-amplified PCR products of *wda1* gene (Primer-U: ATGGCCACAAACCCAGGAC, Primer-L: ACCTTGAGCAACTGGGCAG) were integrated into the T-vector (pMD19, Takara) by direct ligation reaction. The plasmids were transformed into competent cells of *E*. *coli* and monoclonal colonies were sequenced by Sanger reaction. Finally, we obtained transcript isoforms of *wda1* from wild-type and mutant plants and compared differences in transcripts between them, of which the first transcript type was consistent with the annotated version, denoted here as Os-*wda1*-A, and the other transcript had an alternative 5’ splice site of the second intron, with 20 nucleotides forward moved, and was designated as Os-*wda1*-B.

### Western blotting

To assess whether mutants of Os-*wda1*-B exhibit differential expression of the WDA1 protein, we performed western blot experiments using three polypeptides (blb1-CKKNIKVTMTNKQDYHLLKPE, blb2-PYSQFPPKMVRKDSCSYSTT, and blb3-CGDKVLDMDKVWSAAI) at the C-terminal (wax domain) of WDA1 protein as antigens for antibody preparation in rabbit (RayBiotech; Guangzhou, China). Rabbit serum (antibody titer>8000) was extracted at 8–10 weeks post immunization to detect antibody titer, tested for antibody recognition by ELISA. Volumes for the antibodies (blb1’, blb2’, blb3’) were 100ul each.

Rice protein was extracted from wild-type and mutant spikelets using Plant Total Protein Lysis Buffer (#C500011, Sangon Biotech). Concentration of protein in the supernatant was quantified using Non-Interference Protein Assay (#C503071, Sangon Biotech) and diluted to uniform concentration (3 mg/ml) in lysis buffer. Proteins were separated by 10% sodium dodecyl sulphate–polyacrylamide gel electrophoresis (SDS-PAGE) and electro-transferred to PVDF membranes. Membranes were blocked in dilute non-fat dry milk for 1.5h and probed with primary antibody blb1’, blb2’, blb3’ or anti-plant actin mouse monoclonal antibody, followed by incubation with Secondary Antibodies—Goat Anti-Rabbit IgG-HRP and Goat Anti-Mouse IgG HRP respectively. Immunoreactivity was visualized by chemiluminescent substrate detection with ECL detection system (Tanon 4600). Antibody blb2’ performed the best of the three primary antibodies in preliminary tests and was used for data collection with the target protein WDA1.

### amiRNA vector construction

To explore the role of NMD in the balance of the relative level of PTC/non-PTC alleles, artificial miRNAs (amiRNAs) were designed to knock down the expression of NMD pathway homologs in Rice[35]. The amiRNA vectors were composed of an endogenous rice miRNA precursor osa-MIR528 with the miRNA and miRNA* replaced by the specific 21 bp to the NMD-related genes, including UPF1 (LOC_Os07g31340), UPF2 (LOC_Os02g42040), UPF3 (LOC_Os04g35920), SMG7 (LOC_Os08G21350) and UPF1-like (LOC_Os03g38990). We used the WMD3 (http://wmd3.weigelworld.org/cgi-bin/webapp.cgi) to design the amiRNAs (21 bp) that specific to the target genes based on the annotation of Oryza sativa cDNA v6.1(MSU). Two amiRNAs target different sits were selected for each gene. We used the plasmid PNW55 as the template to construct the amiRNAs precursor with the miRNA and miRNA* of osa-MIR528 replaced amiRNA and amiRNA*. For each amiRNA construct, three fragments amplified by primers PNW55-U+II, I+IV and III+ PNW55-L were linking by recombinase (ClonExpress MultiS One Step Cloning Kit, Vazyme). The primers were shown in S15 Table. The expression of modified precursor was driven by maize ubiquitin promoter that amplified from plasmid pH-A3A-PBE (from Addgene). They were cloned into the backbone pCAMBIA1300 and transformed into Agrobacterium tumefaciens strains EHA105 for rice transformation, Two rice varieties, Kasalath and TP309 as receptor.

### Comparison of natural frameshift mutations in NBS-LRR genes

To search for natural frameshift mutations within homologous genes, we mapped Illumina reads of Tetep (NCBI SRA Run ID: SRR8241154, SRR8241153) to the Nipponbare reference genome using BWA aligner (BWA-MEM 0.7.10-r789) [82] or vice versa (map Nipponbare reads to Tetep genome, https://figshare.com/articles/Datasets_for_Tetep_genome_analysis/7775810/1). Variants between the Tetep and Nipponbare genomes were identified using GATK HaplotypeCaller [83], and putative effects of these variants within NBS-LRR genes were predicted using SnpEffect [84] based on MSU release v.7 [48]. Predicted effects represented variant transcripts of Tetep with a Nipponbare framework, and those with a frameshift mutation were retained for further analysis. Real transcripts of NBS-LRR genes from Tetep were obtained by targeted PCR amplification (S16 Table) in Tetep plants (14 days old) and high-throughput sequenced as described above.

### Estimation of nucleotide diversity between and within rice groups

Based on the genome-wide alternative splicing data detected in current study, for each gene we obtained the regions where alternative splicing occurs and the regions where exonic sequence is not affected by alternative splicing. This we did by comparing the canonical junction and the non-canonical junctions. According to the classification of alternative splicing, these regions were further categorized into: canonical exon (CE), unaffected exonic parts (UE), skipped exon(SE), alternative 5’-exon extension(A5E), Alternative 5’-exon shortening(A5S), Alternative 3’-exon extension(A3E) and Alternative 3’-exon shortening(A3S), and unaffected intronic parts adjacent to non-canonical exon regions (UI). A schematic representation of each type is given in S10 Fig. Unaffected exon parts (UE) refers to the segments of an exon that might have extensions or contractions but is unaffected by these.

To estimate the nucleotide diversities for each region, the ancestral states were inferred by PAML [85] among 20 *indica* varieties and 20 *japonica* varieties (S17 Table) and 20 African rice *O*. *glaberrima* (NCBI SRA Run ID: SRR1206500-SRR1206519). Only regions properly covered by all 60 varieties were included for variants calling and downstream calculation.

For analysis of nucleotide diversity (*π*) between rice groups of *indica* and *japonica*, we adopted the formula introduced in Nei and Li (Nei & Li, 1979). The basic formula was written as:

$$\pi =\sum_{ij}x_{i}x_{j}\pi_{ij}$$

in which *x_i_* and *x_j_* are the respective frequencies of the *i*-th and *j-*th sequences in the groups of *indica* and *japonica*. *π_ij_* is the number of nucleotide differences per nucleotide site between the *i*-th and *j*-th sequences.

Based on this formula, we performed the calculation separately on six types of nucleotide substitutions, i.e., C:G→A:T (means C→A or G→T changes), C:G→T:A, C:G→G:C, A:T → C:G, A:T → G:C, and A:T →T:A, and corrected their nucleotide diversities either by regional GC content (for changes from C:G to others) or by AT content (for changes from A:T to others). For instance, for calculation the nucleotide diversity (*π*′) of C:G→A:T changes, the adjusted formula was

$$\pi \prime =\frac{\pi_{C:G\to A:T}}{GC\_content}$$

in which *π*_C:G→A:T_ was obtained from formula [1] but only counting changes from C:G→A:T, and *GC_content* was obtained after dividing the numbers of the C or G sites by the total sites within the calculated regions.

The percentage of the lengths of each region to the lengths of genome as well as the genes they located are shown in S11 Fig.

### DNA methylation level analysis in different exonic/intronic regions

DNA methylation levels in different regions were calculated using the methylation data of four wild-type Nipponbare samples (NCBI SRA Run ID: SRR1448321, SRR5574081, SRR5574082, SRR8060860). The methylation data was first aligned independently to Nipponbare reference genome using Bismark (v0.22.1) [86] with default parameters. The obtained alignments were processed with “deduplicate_bismark” to remove non-biological PCR duplicates. Subsequently “bismark methylation extractor” was used to extract the methylation call for every single C within the defined splicing regions. Nucleotide positions with insufficient coverage (depth<4) and regions with read coverage of cytosines less than 70% were discarded from further calculation. The final methylation level for each region was calculated as the average methylation level across all four collected samples (S2 Dataset).

## Supporting information

S1 Fig Different forms of rescue junctions related to the indel and distance from boundary of indels to the nearest annotated splice site in the rescued and not rescued mutants. The triangle represents indel. Mosaic parts represent affected annotated exonic parts. (A1) 3n-exon skip: 3n rescue junction that skip the annotated exon which contains the indel (the indel is contained in the skipped exon). (A2-A5) 3n-others: 3n rescue junction only skipped the indel but the annotated exon with the indel was not fully skipped. A nearby annotated exon could be either skipped (A2) or not (A3, A4 and A5). (A6) non-3n-exon skip: non-3n rescue junction compensates the indel through skipping a nearby annotated exon. (A7-A10) non-3n-others: the non-3n rescue junction compensates the indel with shorten exons, which either accompany skipping a nearby annotated exon (A7) or not. (B) Distances from boundary of indels to the nearest annotated splice site in the rescued and not rescued mutants. Light blue box: form-A1, n = 4 (4 genes in 73 are this form); orange box: forms A2-A5, n = 26 (these four forms are not explicitly distinguished currently); grey box: form-A6, n = 4; yellow box: forms A7-A10, n = 33; dark blue box: the mutants without rescue junction, n = 27. As shown in this figure, only with form-A1 (n = 4) the indels are contained in the skipped exons. The detailed distances and relative levels of these 4 genes are shown in S6 Table. There is no heterogeneity between types (ANOVA *P* = 0.28).
(PDF)

S2 Fig The relationship between the proportion of genes with a rescue isoform (or non-canonical junction) and minimum tolerated read depth detected. (A) In 73 mutant loci, the relationship between the proportion of genes with a rescue isoform and minimum read depth detected by RT-PCR-seq. Proportions for each threshold: 21.9% (1000), 17.8%(2000), 16.4%(3000,4000,5000,6000). (B) In wild type of Nipponbare, the relationship between the proportion of genes with non-canonical junction (potential rescue forms) and minimum read depth was detected by the downloaded RNA-seq data of Nipponbare for all genes.
(PDF)

S3 Fig Amino acid sequence comparison of the WDA1 protein in Kasalath relative to the CRISPR mutated lines. “Os-*wda1*-A” represents annotated splicing form, while “Os-*wda1*-B” represents the rescue form. Green highlight indicates the domain of Fatty acid hydroxylase superfamily and the yellow highlight indicates WAX2 C-terminal domain. Mutant of wda1- I, II, III and IV could produce a functional protein with the two domains like WT through the transcript of Os-*wda1*-B.
(PDF)

S4 Fig The expression of two alternative splicing forms (Os-*wda1*-A, Os-*wda1*-B) detected by different methods. (A) Transcripts of wda1 detected by TA cloning. Two main transcripts, Os-wda1-A and Os-wda1-B were shown. (B) Relative expression level of Os-wda1-A and Os-wda1-B detected by RNA-seq. In WT, the relative expression level of Os-wda1-A to the expression of wda1 was as high as 84.0%, and that of Os-wda1-B was only 2.1%. However, in the mutants, the relative expression level changed dramatically, Os-wda1-A was as low as 0.1%, and Os-wda1-B increased to 73.3%. Sample size was 3 for each transcript, respectively. C. Relative expression level of Os-wda1-A and Os-wda1-B detected by RT-PCR-seq.
(PDF)

S5 Fig Alternative splicing produced by different splice junctions. A splicing event is shown by a fold line (indicates a junction) with 5’ and 3’ splice sites. A canonical (or annotated) junction is the splice junction presents in the reference annotation set. A non-canonical junction is defined as the junction that are not present (annotated) in the standard annotation set.
(PDF)

S6 Fig Relationship between the data size of RNA-seq that mapped to the genome and numbers of junctions detected. 1GB data size corresponds to a coverage depth of ~17.0 times of the rice reference transcriptome.
(PDF)

S7 Fig Relationship between the proportion of junctions replicated in RNA-seq and the reads detected both in RT-PCR-seq and RNA-seq. (PDF)

S8 Fig Flowchart of data processing for RNA-seq and RT-PCR-seq. (PDF)

S9 Fig Expression levels of *WDA1* in wild-type rice. (A) Expression levels of *WDA1* at nine different stages of growth. (B) Expression levels of *WDA1* in five different tissues. Bottom number represents sample size for each category.
(PDF)

S10 Fig Definition of each region within a gene based on reference genome annotation and assembled RNA-seq data. Canonical exon (CE): annotated exon regions that do not harbor non-canonical splicing site at both ends; UE: unaffected exonic part adjacent to non-canonical splicing site, SE: skipped exon, A5E: alternative 5’ splice site -exon extension, A5S: alternative 5’ splice site -exon shortening, A3E: alternative 3’ splice site -exon extension, A3S: alternative 3’ splice site -exon shortening. UI: unaffected intronic part to non-canonical exon regions. The red line represents non-canonical splicing site and junction different from annotation, the green line represents the same splicing site and junction as annotation.
(PDF)

S11 Fig Percentages of the lengths of each type of regions to the lengths of genome and genes they located. (A) Percentages of the lengths of each type of regions to the lengths of genome. (B) Percentages of the lengths of each type of regions to the lengths of corresponded genes.
(PDF)

S12 Fig The percentage difference in replicates between the same and different tissues with the read depth. (PDF)

S13 Fig Nucleotide diversity of six types of SNPs in different regions between groups of indica and japonica corrected by local GC content. (A) Nucleotide diversity of regions from single exon skipping events. (B) Nucleotide diversity of regions from multi-exon skipping events. (C) Nucleotide diversity of regions from A5’- exon shortening events. (D) Nucleotide diversity of regions from A3’- exon shortening events. (E) Nucleotide diversity of regions from A5’- exon extension events. (F) Nucleotide diversity of regions from A3’- exon extension events. Error bar: mean +/- sem. Significance tests: two tailed t-test, *P ≤ 0.000521, **P ≤ 0.000104, ***P ≤ 1.04 × 10^−5^ after correction for multiple comparisons (Bonferroni correction).
(PDF)

S14 Fig The average methylation level of CpG, CHG and CHH sites across each region. Regions with read depth < 4 or coverage < 70% were discarded from calculation. Significance tests: two tailed t-test, *P ≤ 0.001042, **P ≤0.000208, ***P ≤ 0.0000208 (Bonferroni corrected P-values).
(PDF)

S1 Table CRISPR/Cas9 spacers, receptor varieties, and introduced mutations. (PDF)

S2 Table Primers for cDNA amplification in mutants. (PDF)

S3 Table Statistics of successfully amplified CRISPR/Cas9 mutant gene loci and corresponding junctions. (PDF)

S4 Table The relative level of rescue form of the two alleles (WT vs mutant) in the wt/indel heterozygotes. (PDF)

S5 Table The genes potentially rescued by exon skipping. (PDF)

S6 Table Mutants with high (junction reads/ junction region depth>10%) relative levels of expression of rescue junctions. (PDF)

S7 Table Downloaded RNA-seq data used in this analysis. (PDF)

S8 Table The numbers of different types of AS events detected by downloaded RNA-seq data. (PDF)

S9 Table Junctions detected in 55 downloaded RNA-seq datasets. (PDF)

S10 Table Primers for cDNA amplification in Nipponbare reference genome. (PDF)

S11 Table Comparison of junctions detected from downloaded RNA-seq datasets and local RT-PCR-seq data. (PDF)

S12 Table Comparison of “non-canonical depth/ canonical depth” between WT and CRISPR experiments and “rescue junction depth/ canonical depth” of rescued genes. (PDF)

S13 Table Predicted effects of AS on Nipponbare and Tetep homologous genes. (PDF)

S14 Table Comparisons between Tophat and StringTie in identifying splice junctions. (PDF)

S15 Table Primers used in the construction of amiRNA vectors. (PDF)

S16 Table Primers for cDNA amplification of NBS genes in Nipponbare and Tetep. (PDF)

S17 Table Information of the 40 rice cultivars downloaded from The 3000 Rice Genomes Project. (PDF)

S1 Text No robust evidence for tissue specific splice junctions. (DOCX)

S2 Text Molecular evolutionary analysis supports the hypothesis that most exon extensions are noise. (DOCX)

S3 Text Comparison between two software, Tophat and StringTie, in identifying splice junctions. (DOCX)

S1 Dataset The influence of rescue junction on protein domain and protein similarity of mutants. (XLSX)

S2 Dataset Computer code for used in this study. (ZIP)

S3 Dataset Nucleotide sequences of *WDA1* gene in wild-type Kasalath and *wda1* mutants in this study. (TXT)
