# Supplementary figures and images for: In rice splice variants that restore the reading frame after frameshifting indel introduction are common, often induced by the indels and sometimes lead to organism-level rescue
Source: PLoS Genet. 2022 Feb 18;18(2):e1010071. doi: 10.1371/journal.pgen.1010071 (PMC8893660; doi:10.1371/journal.pgen.1010071)

S1 Fig

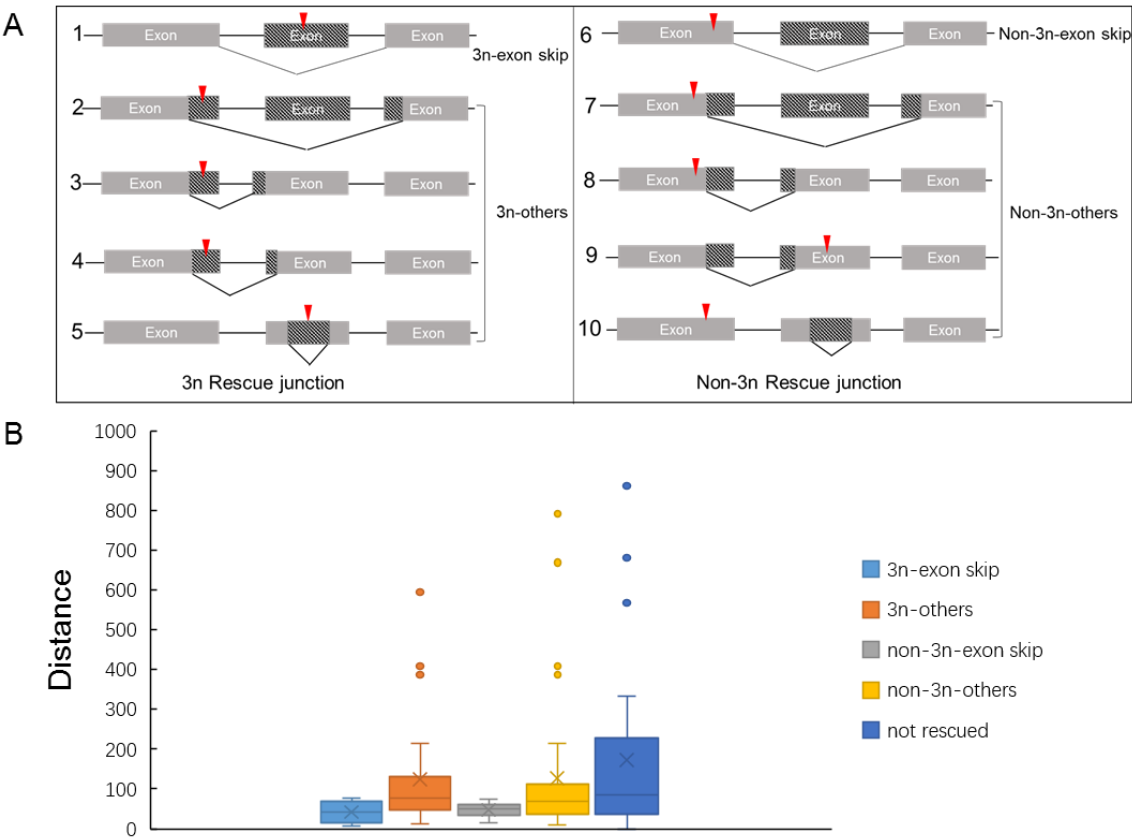

Supplement: S1 Fig — The triangle represents indel. Mosaic parts represent affected annotated exonic parts. (A1) 3n-exon skip: 3n rescue junction that skip the annotated exon which contains the indel (the indel is contained in the skipped exon). (A2-A5) 3n-others: 3n rescue junction only skipped the indel but the annotated exon with the indel was not fully skipped. A nearby annotated exon could be either skipped (A2) or not (A3, A4 and A5). (A6) non-3n-exon skip: non-3n rescue junction compensates the indel through skipping a nearby annotated exon. (A7-A10) non-3n-others: the non-3n rescue junction compensates the indel with shorten exons, which either accompany skipping a nearby annotated exon (A7) or not. (B) Distances from boundary of indels to the nearest annotated splice site in the rescued and not rescued mutants. Light blue box: form-A1, n = 4 (4 genes in 73 are this form); orange box: forms A2-A5, n = 26 (these four forms are not explicitly distinguished currently); grey box: form-A6, n = 4; yellow box: forms A7-A10, n = 33; dark blue box: the mutants without rescue junction, n = 27. As shown in this figure, only with form-A1 (n = 4) the indels are contained in the skipped exons. The detailed distances and relative levels of these 4 genes are shown in S6 Table. There is no heterogeneity between types (ANOVA P = 0.28). (PDF) [file pgen.1010071.s001.pdf]

S2 Fig

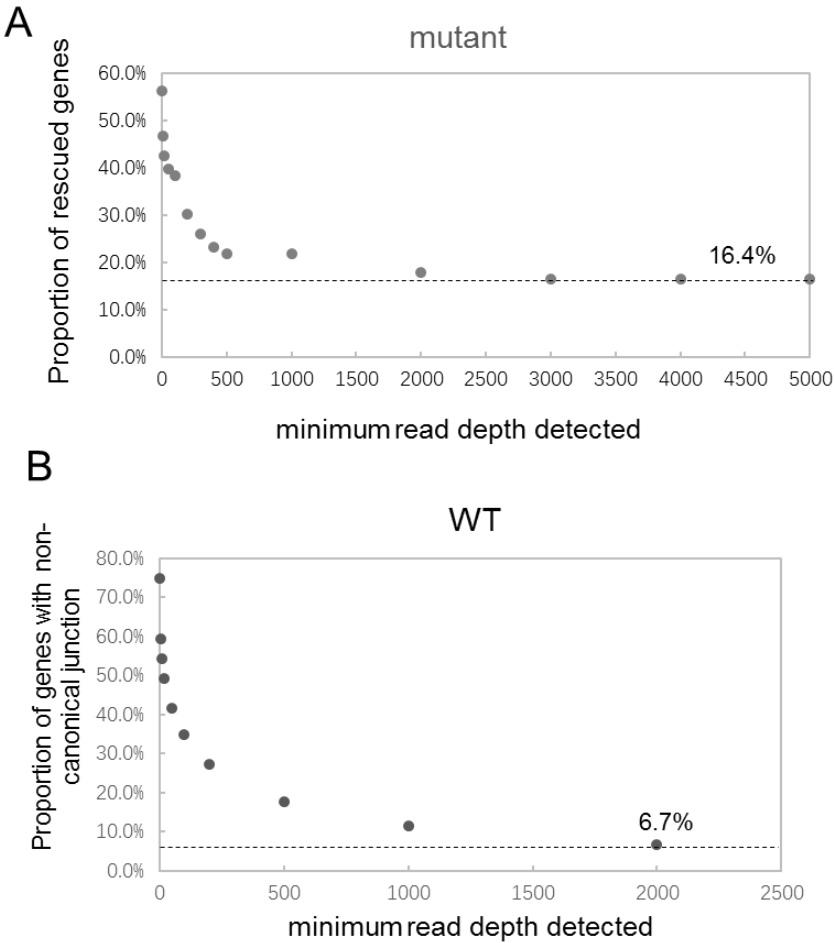

Supplement: S2 Fig — (A) In 73 mutant loci, the relationship between the proportion of genes with a rescue isoform and minimum read depth detected by RT-PCR-seq. Proportions for each threshold: 21.9% (1000), 17.8%(2000), 16.4%(3000,4000,5000,6000). (B) In wild type of Nipponbare, the relationship between the proportion of genes with non-canonical junction (potential rescue forms) and minimum read depth was detected by the downloaded RNA-seq data of Nipponbare for all genes. (PDF) [file pgen.1010071.s002.pdf]

**S4 Fig**

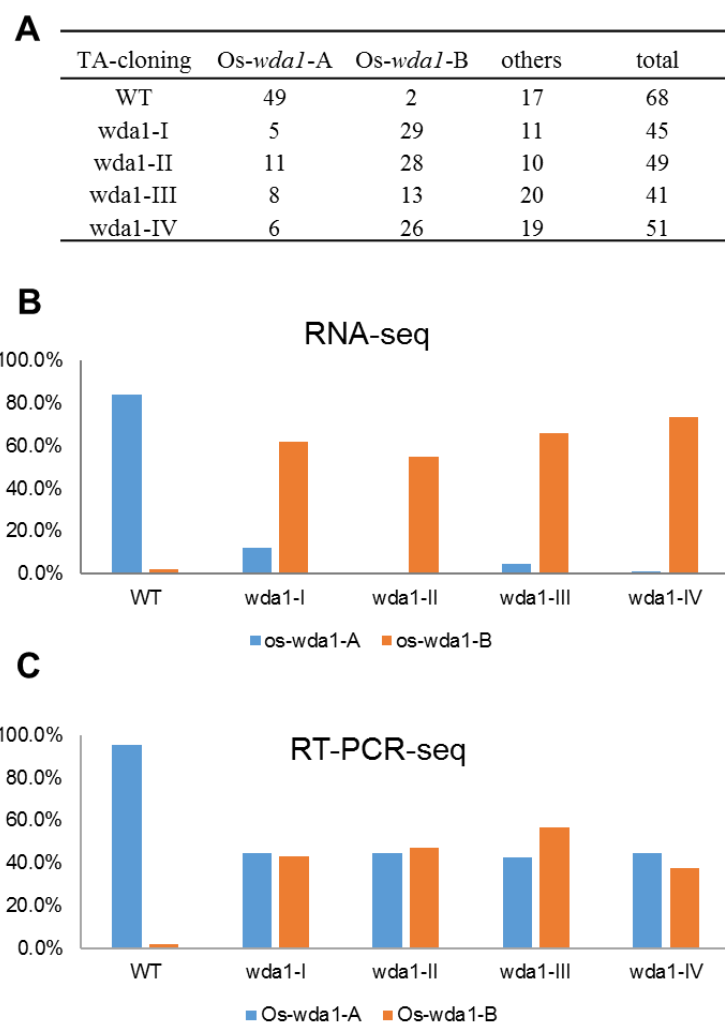

Supplement: S4 Fig — (A) Transcripts of wda1 detected by TA cloning. Two main transcripts, Os-wda1-A and Os-wda1-B were shown. (B) Relative expression level of Os-wda1-A and Os-wda1-B detected by RNA-seq. In WT, the relative expression level of Os-wda1-A to the expression of wda1 was as high as 84.0%, and that of Os-wda1-B was only 2.1%. However, in the mutants, the relative expression level changed dramatically, Os-wda1-A was as low as 0.1%, and Os-wda1-B increased to 73.3%. Sample size was 3 for each transcript, respectively. C. Relative expression level of Os-wda1-A and Os-wda1-B detected by RT-PCR-seq. (PDF) [file pgen.1010071.s004.pdf]

S5 Fig

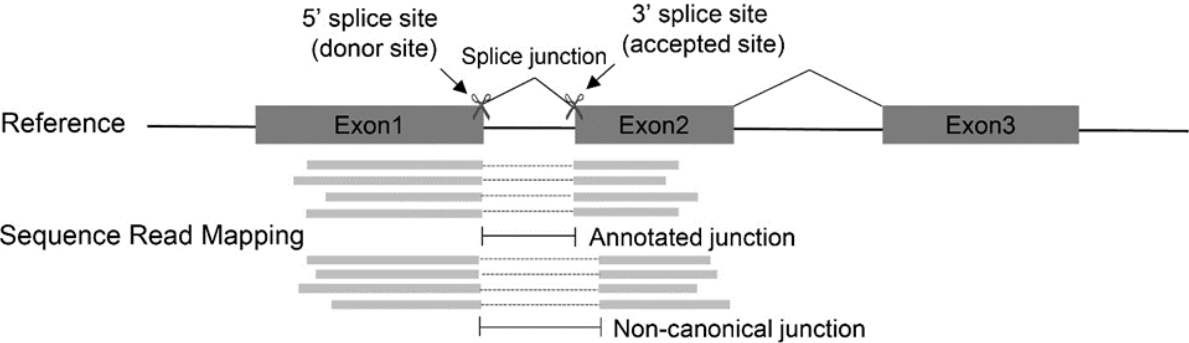

Supplement: S5 Fig — A splicing event is shown by a fold line (indicates a junction) with 5’ and 3’ splice sites. A canonical (or annotated) junction is the splice junction presents in the reference annotation set. A non-canonical junction is defined as the junction that are not present (annotated) in the standard annotation set. (PDF) [file pgen.1010071.s005.pdf]

S6 Fig

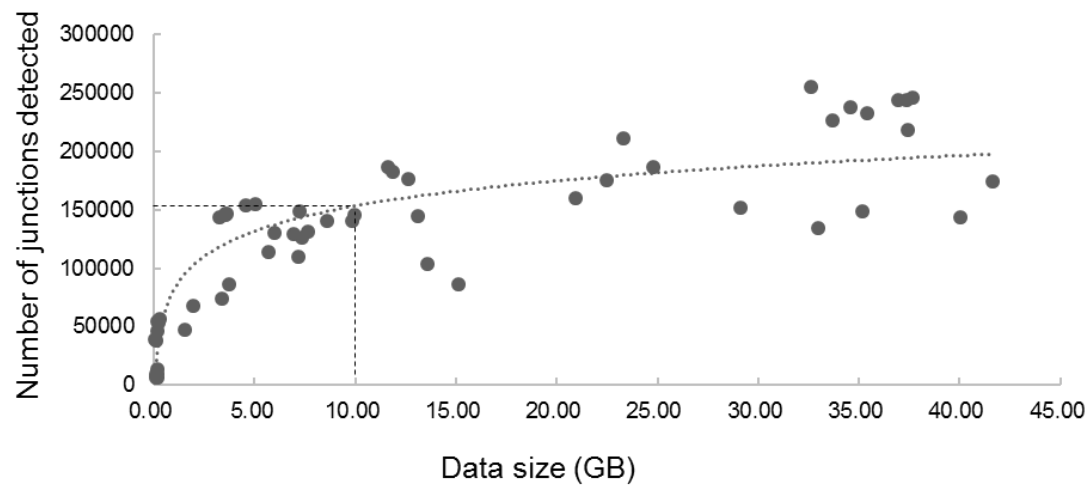

Supplement: S6 Fig — 1GB data size corresponds to a coverage depth of ~17.0 times of the rice reference transcriptome. (PDF) [file pgen.1010071.s006.pdf]

**S7 Fig**

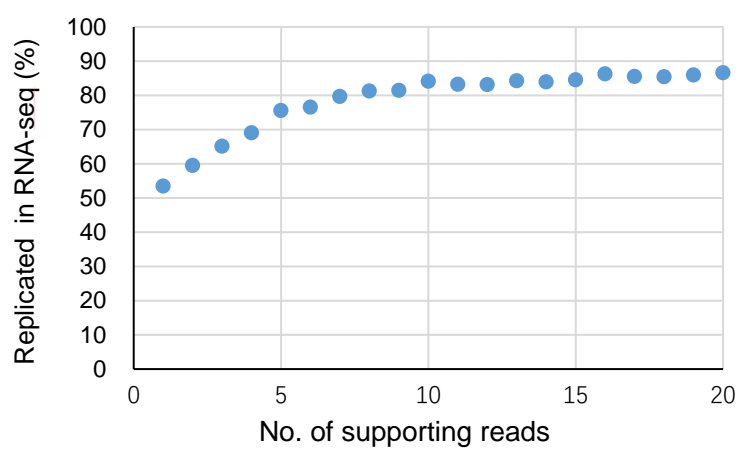

Supplement: S7 Fig — (PDF) [file pgen.1010071.s007.pdf]

**S8 Fig**

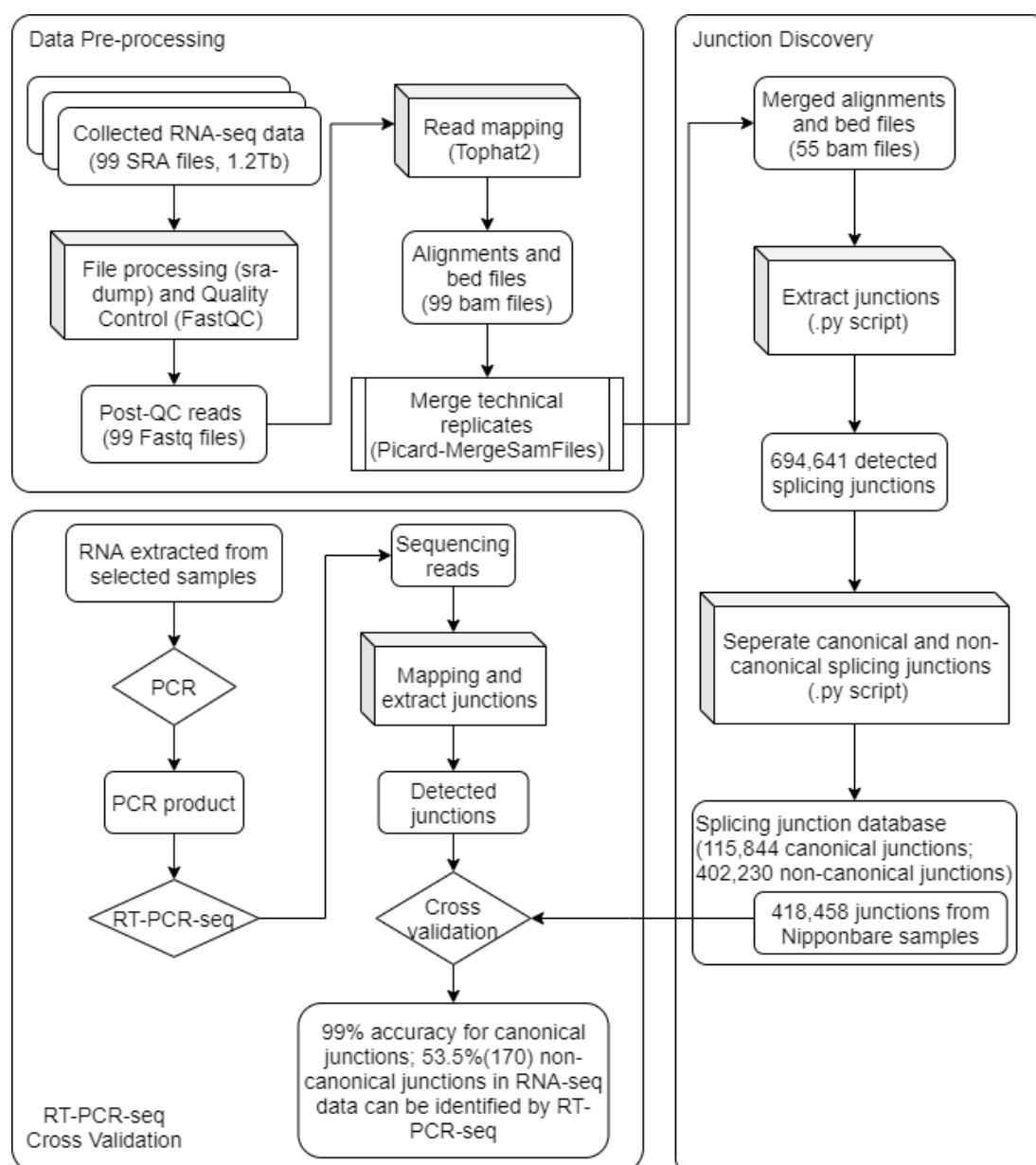

Supplement: S8 Fig — (PDF) [file pgen.1010071.s008.pdf]

S9 Fig

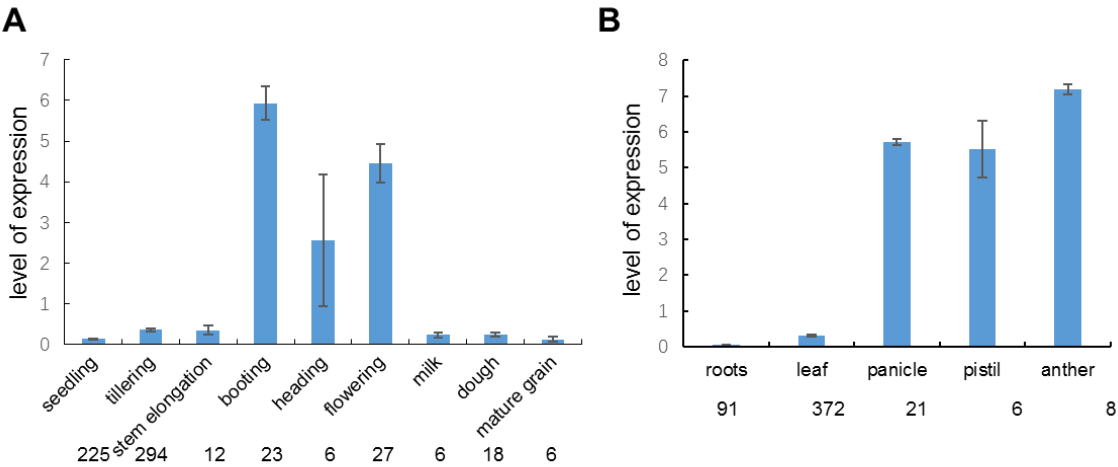

Supplement: S9 Fig — (A) Expression levels of WDA1 at nine different stages of growth. (B) Expression levels of WDA1 in five different tissues. Bottom number represents sample size for each category. (PDF) [file pgen.1010071.s009.pdf]

S10 Fig

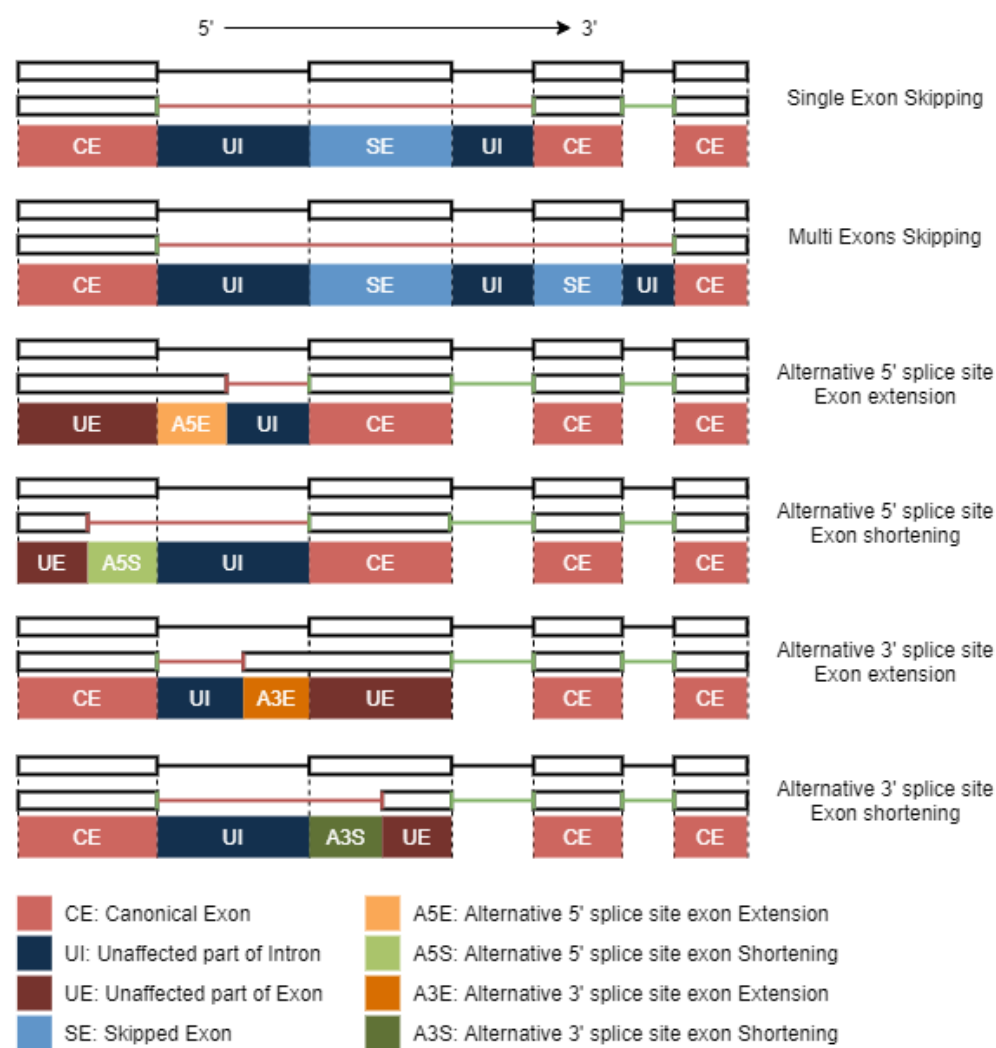

Supplement: S10 Fig — Canonical exon (CE): annotated exon regions that do not harbor non-canonical splicing site at both ends; UE: unaffected exonic part adjacent to non-canonical splicing site, SE: skipped exon, A5E: alternative 5’ splice site -exon extension, A5S: alternative 5’ splice site -exon shortening, A3E: alternative 3’ splice site -exon extension, A3S: alternative 3’ splice site -exon shortening. UI: unaffected intronic part to non-canonical exon regions. The red line represents non-canonical splicing site and junction different from annotation, the green line represents the same splicing site and junction as annotation. (PDF) [file pgen.1010071.s010.pdf]

S11 Fig

**A**

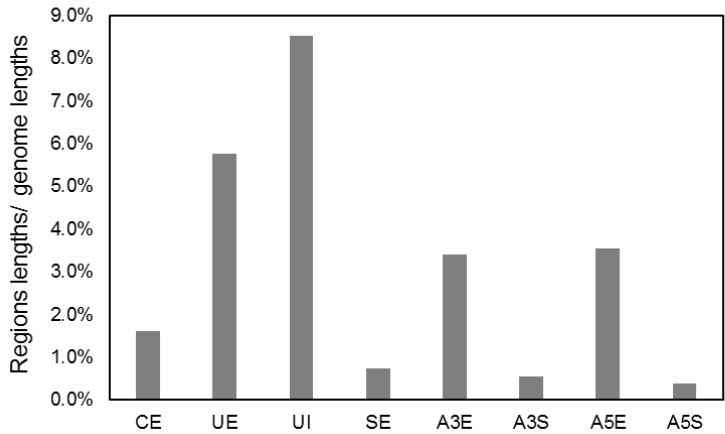

**B**

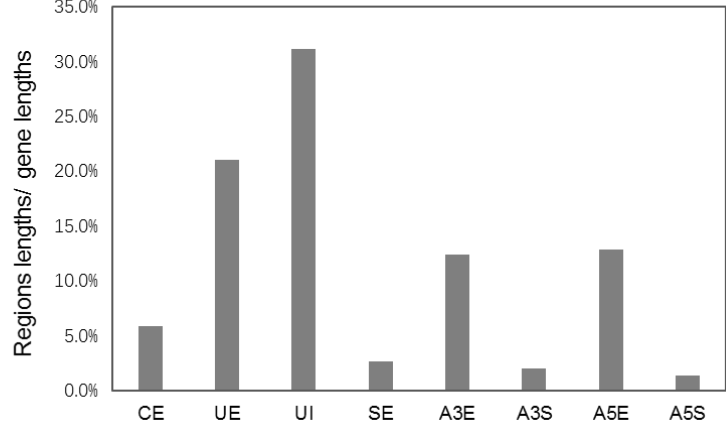

Supplement: S11 Fig — (A) Percentages of the lengths of each type of regions to the lengths of genome. (B) Percentages of the lengths of each type of regions to the lengths of corresponded genes. (PDF) [file pgen.1010071.s011.pdf]

S12 Fig

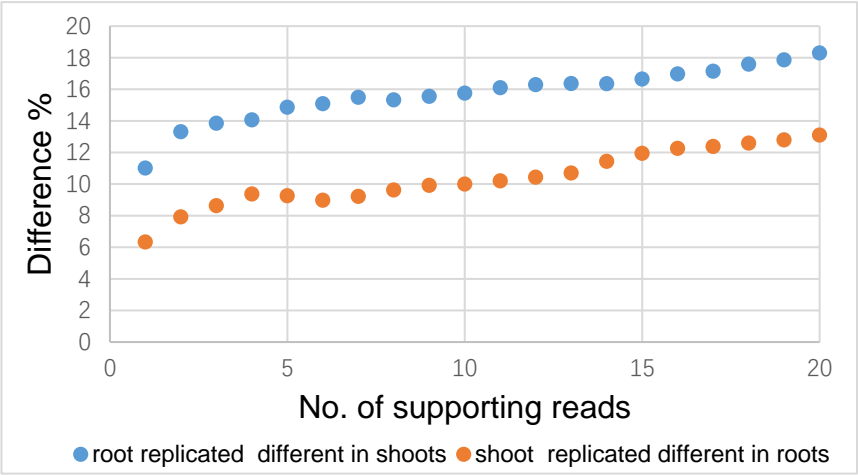

Supplement: S12 Fig — (PDF) [file pgen.1010071.s012.pdf]

S13 Fig

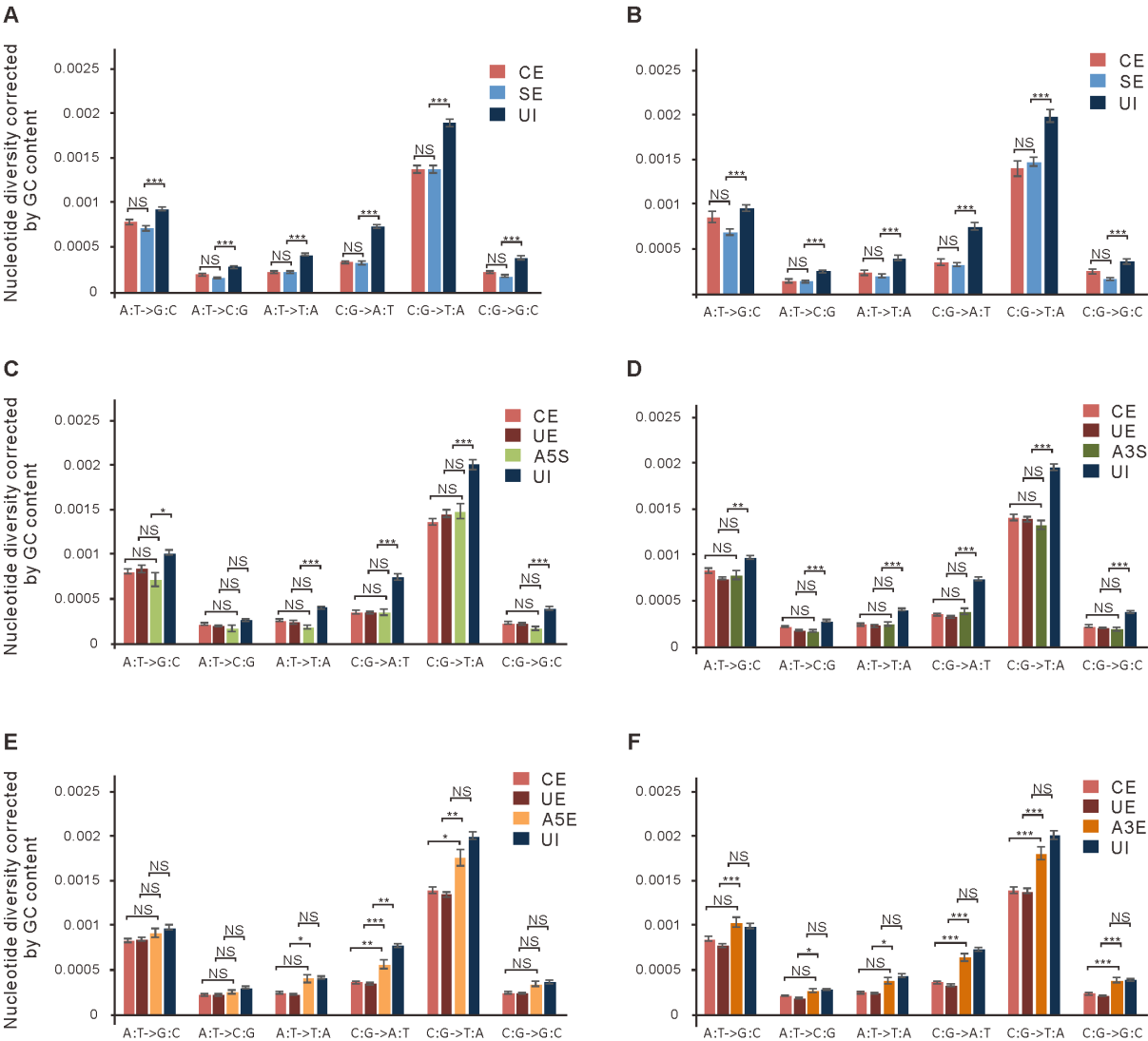

Supplement: S13 Fig — (A) Nucleotide diversity of regions from single exon skipping events. (B) Nucleotide diversity of regions from multi-exon skipping events. (C) Nucleotide diversity of regions from A5’- exon shortening events. (D) Nucleotide diversity of regions from A3’- exon shortening events. (E) Nucleotide diversity of regions from A5’- exon extension events. (F) Nucleotide diversity of regions from A3’- exon extension events. Error bar: mean +/- sem. Significance tests: two tailed t-test, *P ≤ 0.000521, **P ≤ 0.000104, ***P ≤ 1.04 × 10^−5^ after correction for multiple comparisons (Bonferroni correction). (PDF) [file pgen.1010071.s013.pdf]

S14 Fig

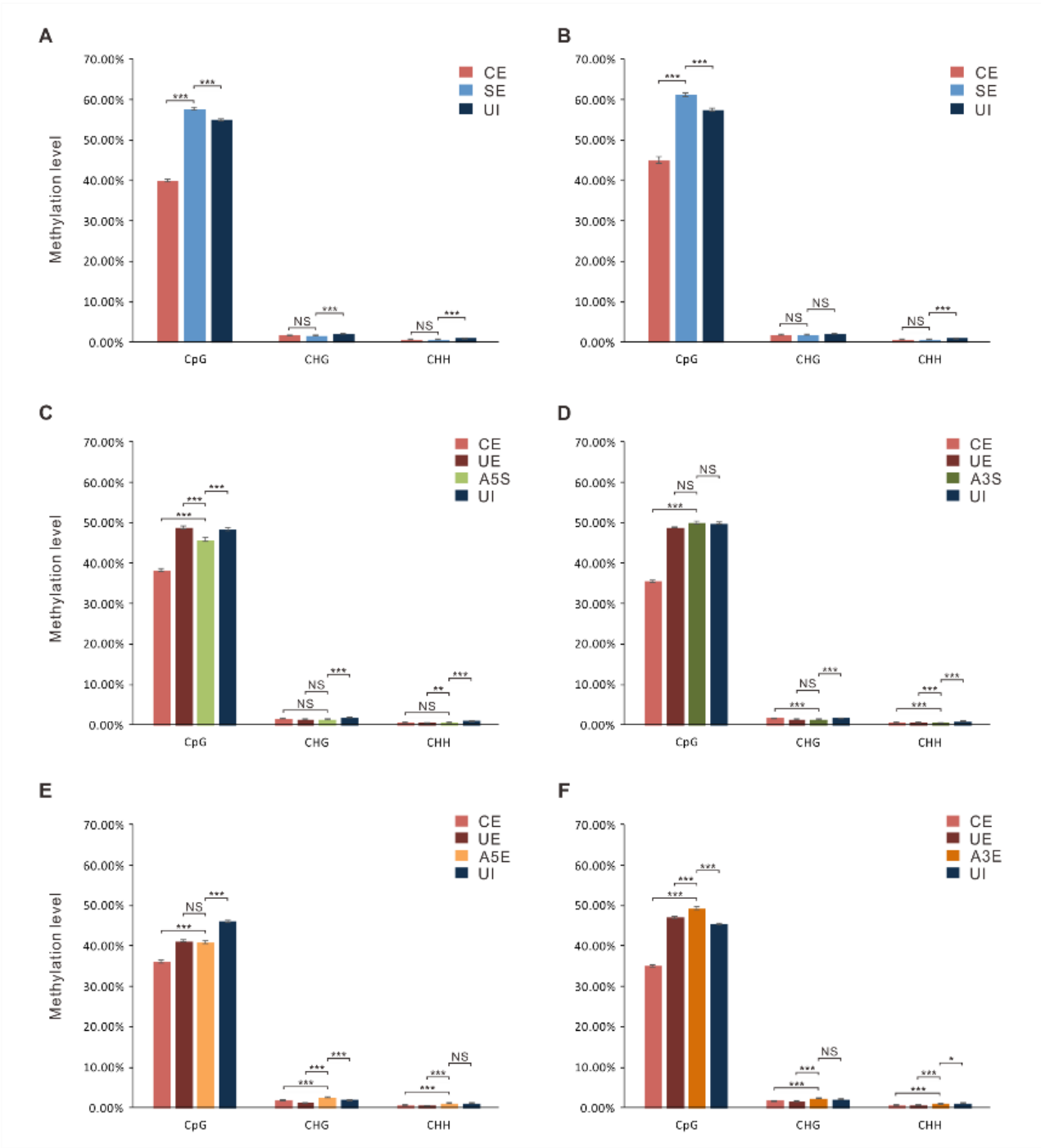

Supplement: S14 Fig — Regions with read depth < 4 or coverage < 70% were discarded from calculation. Significance tests: two tailed t-test, *P ≤ 0.001042, **P ≤0.000208, ***P ≤ 0.0000208 (Bonferroni corrected P-values). (PDF) [file pgen.1010071.s014.pdf]
