# Supplementary material for: In rice splice variants that restore the reading frame after frameshifting indel introduction are common, often induced by the indels and sometimes lead to organism-level rescue
Source: PLoS Genet. 2022 Feb 18;18(2):e1010071. doi: 10.1371/journal.pgen.1010071 (PMC8893660; doi:10.1371/journal.pgen.1010071)
Supplement: S3 Fig — “Os-wda1-A” represents annotated splicing form, while “Os-wda1-B” represents the rescue form. Green highlight indicates the domain of Fatty acid hydroxylase superfamily and the yellow highlight indicates WAX2 C-terminal domain. Mutant of wda1- I, II, III and IV could produce a functional protein with the two domains like WT through the transcript of Os-wda1-B. (PDF) [file pgen.1010071.s003.pdf]

S3 Fig

```
Wild-type_Os-wda1-A     : MATNPGLFTEWPWKKLGSFKYVLLAPWVAHGWYEVATKGRREVDLGYIAILPSLLLRMLHNQAWITISRLQNARGRRQIVRRGIEFDQVDRERNWDDQII : 100
Wild-type_Os-wda1-B     : MATNPGLFTEWPWKKLGSFKYVLLAPWVAHGWYEVATKGRREVDLGYIAILPSLLLRMLHNQAWITISRLQNARGRRQIVRRGIEFDQGRPDHPERYPAI : 100
Mutant(-1bp)_Os-wda1-A  : MATNPGLFTEWPWKKLGSFKYVLLAPWVAHGWYEVATKGRREVDLGYIAILPSLLLRMLHNQAWITISRLQNARGRRQIVRRGIEFDQVDRERNWDDQII : 100
Mutant(-1bp)_Os-wda1-B  : MATNPGLFTEWPWKKLGSFKYVLLAPWVAHGWYEVATKGRREVDLGYIAILPSLLLRMLHNQAWITISRLQNARGRRQIVRRGIEFDQGRPDHPERYPAI : 100
Mutant(-4bp)_Os-wda1-A  : MATNPGLFTEWPWKKLGSFKYVLLAPWVAHGWYEVATKGRREVDLGYIAILPSLLLRMLHNQAWITISRLQNARGRRQIVRRGIEFDQVDRERNWDDQII : 100
Mutant(-4bp)_Os-wda1-B  : MATNPGLFTEWPWKKLGSFKYVLLAPWVAHGWYEVATKGRREVDLGYIAILPSLLLRMLHNQAWITISRLQNARGRRQIVRRGIEFDQGRPDHPERYPAI : 100
Mutant(-22bp)_Os-wda1-A : MATNPGLFTEWPWKKLGSFKYVLLAPWVAHGWYEVATKGRREVDLGYIAILPSLLLRMLHNQAWITISRLQNARGRRQIVRRGIEFDQVDRERNWDDQII : 100
Mutant(-22bp)_Os-wda1-B : MATNPGLFTEWPWKKLGSFKYVLLAPWVAHGWYEVATKGRREVDLGYIAILPSLLLRMLHNQAWITISRLQNARGRRQIVRRGIEFDQGRPDHPERYPAI : 100
Mutant(+1bp)_Os-wda1-A  : MATNPGLFTEWPWKKLGSFKYVLLAPWVAHGWYEVATKGRREVDLGYIAILPSLLLRMLHNQAWITISRLQNARGRRQIVRRGIEFDQVDRERNWDDQII : 100
Mutant(+1bp)_Os-wda1-B  : MATNPGLFTEWPWKKLGSFKYVLLAPWVAHGWYEVATKGRREVDLGYIAILPSLLLRMLHNQAWITISRLQNARGRRQIVRRGIEFDQGRPDHPERYPAI : 100

Wild-type_Os-wda1-A     : LSGILLYLGALYVPGGQHLPLWRTDGAGLIALLHAGPVEFLYYWFHRALHHHFLYTRYHSHHHSSIVTEPITSVIHPFAELVAYELLFSIPLIACALTGT : 200
Wild-type_Os-wda1-B     : PRRTVRTGRATLTAVEDGWRGADCLAACRASGVPLLLVPSCVAPPLSLHPLPLAPPLLNRH*-------------------------------------- : 161
Mutant(-1bp)_Os-wda1-A  : LSGILLYLGALYVPGGNTYRCGGRMARG*----------------------------------------------------------------------- : 128
Mutant(-1bp)_Os-wda1-B  : PRRTVRTGR-------QHLPLWRTDGAGLIALLHAGPVEFLYYWFHRALHHHFLYTRYHSHHHSSIVTEPITSVIHPFAELVAYELLFSIPLIACALTGT : 193
Mutant(-4bp)_Os-wda1-A  : LSGILLYLGALYVP-GNTYRCGGRMARG*----------------------------------------------------------------------- : 127
Mutant(-4bp)_Os-wda1-B  : PRRTVRTG--------QHLPLWRTDGAGLIALLHAGPVEFLYYWFHRALHHHFLYTRYHSHHHSSIVTEPITSVIHPFAELVAYELLFSIPLIACALTGT : 192
Mutant(-22bp)_Os-wda1-A : LSGILLYLGALY-------RCGGRMARG*----------------------------------------------------------------------- : 121
Mutant(-22bp)_Os-wda1-B : PRRTV--------------PLWRTDGAGLIALLHAGPVEFLYYWFHRALHHHFLYTRYHSHHHSSIVTEPITSVIHPFAELVAYELLFSIPLIACALTGT : 186
Mutant(+1bp)_Os-wda1-A  : LSGILLYLGALYVPGRATLTAVEDGWRGADCLAACRASGVPLLLVPSCVAPPLSLHPLPLAPPLLNRH*------------------------------- : 168
Mutant(+1bp)_Os-wda1-B  : PRRTVRTGQGNTYRCGGRMARG*----------------------------------------------------------------------------- : 122

Wild-type_Os-wda1-A     : ASIIAFEMYLIYIDFMNNMGHCNFELVPSWLFTWFPPLKYLMYTPSFHSLHHTQFRTNYSLFMPFYDYIYNTMDKSSDTLYENSLKNNDEEEAVDVVHLT : 300
Wild-type_Os-wda1-B     : ---------------------------------------------------------------------------------------------------- :  -
Mutant(-1bp)_Os-wda1-A  : ---------------------------------------------------------------------------------------------------- :  -
Mutant(-1bp)_Os-wda1-B  : ASIIAFEMYLIYIDFMNNMGHCNFELVPSWLFTWFPPLKYLMYTPSFHSLHHTQFRTNYSLFMPFYDYIYNTMDKSSDTLYENSLKNNDEEEAVDVVHLT : 293
Mutant(-4bp)_Os-wda1-A  : ---------------------------------------------------------------------------------------------------- :  -
Mutant(-4bp)_Os-wda1-B  : ASIIAFEMYLIYIDFMNNMGHCNFELVPSWLFTWFPPLKYLMYTPSFHSLHHTQFRTNYSLFMPFYDYIYNTMDKSSDTLYENSLKNNDEEEAVDVVHLT : 292
Mutant(-22bp)_Os-wda1-A : ---------------------------------------------------------------------------------------------------- :  -
Mutant(-22bp)_Os-wda1-B : ASIIAFEMYLIYIDFMNNMGHCNFELVPSWLFTWFPPLKYLMYTPSFHSLHHTQFRTNYSLFMPFYDYIYNTMDKSSDTLYENSLKNNDEEEAVDVVHLT : 286
Mutant(+1bp)_Os-wda1-A  : ---------------------------------------------------------------------------------------------------- :  -
Mutant(+1bp)_Os-wda1-B  : ---------------------------------------------------------------------------------------------------- :  -

Wild-type_Os-wda1-A     : HLTTLHSIYHMRPGFAEFASRPYVSRWYMRMMWPLSWLSMVLTWTYGSSFTVERNVMKKIRMQSWAIPRYSFHYGLDWEKEAINDLIEKAVCEADKNGAK : 400
Wild-type_Os-wda1-B     : ---------------------------------------------------------------------------------------------------- :  -
Mutant(-1bp)_Os-wda1-A  : ---------------------------------------------------------------------------------------------------- :  -
Mutant(-1bp)_Os-wda1-B  : HLTTLHSIYHMRPGFAEFASRPYVSRWYMRMMWPLSWLSMVLTWTYGSSFTVERNVMKKIRMQSWAIPRYSFHYGLDWEKEAINDLIEKAVCEADKNGAK : 393
Mutant(-4bp)_Os-wda1-A  : ---------------------------------------------------------------------------------------------------- :  -
Mutant(-4bp)_Os-wda1-B  : HLTTLHSIYHMRPGFAEFASRPYVSRWYMRMMWPLSWLSMVLTWTYGSSFTVERNVMKKIRMQSWAIPRYSFHYGLDWEKEAINDLIEKAVCEADKNGAK : 392
Mutant(-22bp)_Os-wda1-A : ---------------------------------------------------------------------------------------------------- :  -
Mutant(-22bp)_Os-wda1-B : HLTTLHSIYHMRPGFAEFASRPYVSRWYMRMMWPLSWLSMVLTWTYGSSFTVERNVMKKIRMQSWAIPRYSFHYGLDWEKEAINDLIEKAVCEADKNGAK : 386
Mutant(+1bp)_Os-wda1-A  : ---------------------------------------------------------------------------------------------------- :  -
Mutant(+1bp)_Os-wda1-B  : ---------------------------------------------------------------------------------------------------- :  -
```

```
Wild-type_Os-wda1-A     : VVSLGLLNQAHTLNKSGEQYLLKYPKLGARIVDGTSLAAAVVVNSIPQGTDQVILAGNVSKVARAVAQALCKKNIKVTMTNKQDYHLLKPEIPETVADNL : 500
Wild-type_Os-wda1-B     : ---------------------------------------------------------------------------------------------------- :  -
Mutant(-1bp)_Os-wda1-A  : ---------------------------------------------------------------------------------------------------- :  -
Mutant(-1bp)_Os-wda1-B  : VVSLGLLNQAHTLNKSGEQYLLKYPKLGARIVDGTSLAAAVVVNSIPQGTDQVILAGNVSKVARAVAQALCKKNIKVTMTNKQDYHLLKPEIPETVADNL : 493
Mutant(-4bp)_Os-wda1-A  : ---------------------------------------------------------------------------------------------------- :  -
Mutant(-4bp)_Os-wda1-B  : VVSLGLLNQAHTLNKSGEQYLLKYPKLGARIVDGTSLAAAVVVNSIPQGTDQVILAGNVSKVARAVAQALCKKNIKVTMTNKQDYHLLKPEIPETVADNL : 492
Mutant(-22bp)_Os-wda1-A : ---------------------------------------------------------------------------------------------------- :  -
Mutant(-22bp)_Os-wda1-B : VVSLGLLNQAHTLNKSGEQYLLKYPKLGARIVDGTSLAAAVVVNSIPQGTDQVILAGNVSKVARAVAQALCKKNIKVTMTNKQDYHLLKPEIPETVADNL : 486
Mutant(+1bp)_Os-wda1-A  : ---------------------------------------------------------------------------------------------------- :  -
Mutant(+1bp)_Os-wda1-B  : ---------------------------------------------------------------------------------------------------- :  -

Wild-type_Os-wda1-A     : SFSKTGTAKVWLIGDGLDSAEQFRAQKGTLFIPYSQFPPKMVRKDSCSYSTTPAMAVPKTLQNVHSCENWLPRRVMSAWRIAGILHALEGWNEHECGDKV : 600
Wild-type_Os-wda1-B     : ---------------------------------------------------------------------------------------------------- :  -
Mutant(-1bp)_Os-wda1-A  : ---------------------------------------------------------------------------------------------------- :  -
Mutant(-1bp)_Os-wda1-B  : SFSKTGTAKVWLIGDGLDSAEQFRAQKGTLFIPYSQFPPKMVRKDSCSYSTTPAMAVPKTLQNVHSCENWLPRRVMSAWRIAGILHALEGWNEHECGDKV : 593
Mutant(-4bp)_Os-wda1-A  : ---------------------------------------------------------------------------------------------------- :  -
Mutant(-4bp)_Os-wda1-B  : SFSKTGTAKVWLIGDGLDSAEQFRAQKGTLFIPYSQFPPKMVRKDSCSYSTTPAMAVPKTLQNVHSCENWLPRRVMSAWRIAGILHALEGWNEHECGDKV : 592
Mutant(-22bp)_Os-wda1-A : ---------------------------------------------------------------------------------------------------- :  -
Mutant(-22bp)_Os-wda1-B : SFSKTGTAKVWLIGDGLDSAEQFRAQKGTLFIPYSQFPPKMVRKDSCSYSTTPAMAVPKTLQNVHSCENWLPRRVMSAWRIAGILHALEGWNEHECGDKV : 586
Mutant(+1bp)_Os-wda1-A  : ---------------------------------------------------------------------------------------------------- :  -
Mutant(+1bp)_Os-wda1-B  : ---------------------------------------------------------------------------------------------------- :  -

Wild-type_Os-wda1-A     : LDMDKVWSAAIMHGFCPVAQG* : 621
Wild-type_Os-wda1-B     : ---------------------- :  -
Mutant(-1bp)_Os-wda1-A  : ---------------------- :  -
Mutant(-1bp)_Os-wda1-B  : LDMDKVWSAAIMHGFCPVAQG* : 614
Mutant(-4bp)_Os-wda1-A  : ---------------------- :  -
Mutant(-4bp)_Os-wda1-B  : LDMDKVWSAAIMHGFCPVAQG* : 613
Mutant(-22bp)_Os-wda1-A : ---------------------- :  -
Mutant(-22bp)_Os-wda1-B : LDMDKVWSAAIMHGFCPVAQG* : 607
Mutant(+1bp)_Os-wda1-A  : ---------------------- :  -
Mutant(+1bp)_Os-wda1-B  : ---------------------- :  -
```
