# Supplementary material for: In rice splice variants that restore the reading frame after frameshifting indel introduction are common, often induced by the indels and sometimes lead to organism-level rescue
Source: PLoS Genet. 2022 Feb 18;18(2):e1010071. doi: 10.1371/journal.pgen.1010071 (PMC8893660; doi:10.1371/journal.pgen.1010071)
Supplement: S1 Table — (PDF) [file pgen.1010071.s015.pdf]

S1 Table. CRISPR/Cas9 spacers, receptor varieties, and introduced mutations.

Gene Locus	Spacer sequence (5'-3')	Receptor variety	Genotypes of mutants [a]
Os01g0129600	TCCTCGCAAACTAACCCGCG	Kasalath	Failed
Os01g0218032	TCAATTCCGACAATATTTAA	TP309	Failed
Os01g0264000	CTTCGGGCGGGTGATCCCGC	Kasalath	Failed
Os01g0277500	GTGGCAGCGCGGACGAGATC	TP309	Homozygous:-2bp
Os01g0302500	TACCTTGCGACACTCGATGT	TP309	Biallelic:+1bp/-5bp<br>Homozygous:+1bp<br>Homozygous:-5bp
Os01g0616900	CTGGAGCAACACCTGAAGGA	TP309	Heterozygous:+1bp/WT<br>Homozygous:+1bp
Os01g0678700	GCCAACCTGCTCATTCGCGA	Wuyunjing24	Homozygous:+1bp
Os01g0758200	CCACGTCGAGATGGCCCCGG	TP309	Homozygous:-1bp
Os01g0758200	CCACGTCGAGATGGCCCCGG	Kasalath	Homozygous:-1bp
Os01g0818400	GATCCTCTAATGGTTCCCGG	Wuyunjing24	Failed
Os01g0840300	GAGCAGATCGCGGTGCTGGA	Wuyunjing24	Failed
Os01g0884300	GAGAGAGATGAGCGGCGGTC	Wuyunjing24	Homozygous:+1bp
Os01g0884300	GAGAGAGATGAGCGGCGGTC	Kasalath	Homozygous:+1bp<br>Biallelic:+1bp/+3bp<br>Homozygous:+3bp
Os01g0884350	TCCGGTGATCGTCCGTTCTC	Wuyunjing24	Failed
Os01g0884350	TCCGGTGATCGTCCGTTCTC	Kasalath	Homozygous:+1bp
Os01g0884400	GATTGCTCCGCAGTTTGCGA	Kasalath	Biallelic:+1bp/-3bp<br>Homozygous:-3bp<br>Homozygous:+1bp
Os01g0884500	GGTTATCATCCACACTGTGT	Wuyunjing24	Homozygous:+1bp
Os01g0884500	GGTTATCATCCACACTGTGT	Kasalath	Failed
Os01g0885000	CATGCCGTCGACAAGGCCGC	Wuyunjing24	Heterozygous:+1bp/WT<br>Homozygous:+1bp
Os01g0898300	TGCAGTCGAGCCACGGGAAT	Kasalath	Failed
Os01g0909100	ACAGTGATGTGTGATCCACC	Wuyunjing24	Biallelic:-<br>1bp/RPL:CC→T
Os01g0909100	ACAGTGATGTGTGATCCACC	Kasalath	Biallelic:-4bp/-1bp<br>Homozygous:-16bp
Os01g0909200	GTCATTGGAATCATCTTCTC	Kasalath	Failed
Os01g0919900	ATTTCTCGACAGGCTTAAGG	TP309	Heterozygous:-7bp/WT
Os01g0919900	ATTTCTCGACAGGCTTAAGG	Kasalath	Failed
Os01g0922600	GTGGTAGTCGCGGACGCACT	Wuyunjing24	Heterozygous:+1bp/WT<br>Biallelic:+1bp/-2bp
Os01g0927600	AGAGGAAGGGGGTTAACAGG	Wuyunjing24	Failed
Os01g0927900	AGCAGCTCGTATCGGTGTCC	Wuyunjing24,<br>Kasalath	Failed

Os01g0928100	TTCTGCCGACTGCGGTCCAC	Kasalath	Failed
Os01g0928300	GCCGAGCTCGGCCGCCTCCA	Kasalath	Heterozygous:-2bp/WT
Os01g0930800	ATCCGAAGACGAAATCCTCC	Kasalath	Homozygous:-2bp<br>Homozygous:+1bp<br>Biallelic:+1bp/-2bp
Os01g0936100	CAGTCAAGAGGTTCCTTATC	Kasalath	Homozygous:-3bp
Os01g0936100	CAGTCAAGAGGTTCCTTATC	Wuyunjing24	Homozygous:+1bp
Os02g0125600	GAAGGTGTGTTACCCCGCGA	TP309	Homozygous:+1bp
Os02g0139400	TCAGAAGAGCTCGGTGTTGT	Wuyunjing24	Homozygous:-1bp
Os02g0174100	CCAGGTGGAAGGGTGCGGGG	TP309	Homozygous:-2bp
Os02g0174100	CCAGGTGGAAGGGTGCGGGG	Wuyunjing24	Biallelic:+1bp/-2bp
Os02g0252400	CGGCGCATTATCCCCGGTGG	Wuyunjing24	Biallelic:+1bp(A/C)<br>Biallelic:+1bp(T/C)<br>Homozygous:+1bp
Os02g0259600	TACTCAAGTATCGGGTCAGC	Kasalath	Homozygous:+1bp
Os02g0274600	GCCCGTTAGGTCCGCACCAG	Kasalath	Failed
Os02g0312600	CTGAAGTTGTCGAAAACCGT	TP309	Heterozygous:-9bp/WT<br>Biallelic:-1bp/-9bp
Os02g0496100	TTGGCAAATTCCCATCGCTC	Kasalath	Failed
Os02g0529400	TGGCCCTCCACAGTATGCCG	Kasalath	Heterozygous:+1bp/WT<br>Biallelic:+1bp(G/T)
Os02g0529400	TGGCCCTCCACAGTATGCCG	TP309	Heterozygous:+1bp/WT<br>Biallelic:-<br>3bp/RPL:ATG→GCGA
Os02g0553200	AATCAGCATAAGTTATACCC	TP309	Homozygous:/-1bp
Os02g0606200	GGTGCACGCGGCGAACAAGC	TP309	Heterozygous:-1bp/WT<br>Heterozygous:+1bp/WT<br>Homozygous:+1bp<br>Homozygous:-1bp
Os02g0678800	ACCGGCATTCGGGTCAGCGC	Wuyunjing24	Heterozygous:-1bp/WT
Os02g0707200	GGCATCGTCCCCGAGGAGCA	TP309	Biallelic:-2bp/+2bp<br>Homozygous:-3bp<br>Homozygous:+1bp
Os02g0726300	GCAGGAGTTCCAGTCCATCC	TP309	Biallelic:+1bp/-1bp
Os02g0777400	TTGTGTACGAAGATATAATG	Wuyunjing24	Heterozygous:+1bp/WT
Os03g0255400	TCCCAGAGTCATTACTGACA	Kasalath	Heterozygous:-4bp/WT<br>Heterozygous:-1bp/WT
Os03g0276300	GCGGCGCCGGGCGAGATCGG	TP309	chimeric[b]:-2bp/+1bp/-<br>1bp/WT
Os03g0297400	GTGGAGGCCATCGTAGACAC	TP309	Heterozygous:-28bp/WT
Os03g0297400	GTGGAGGCCATCGTAGACAC	Kasalath	Homozygous:+1bp
Os03g0408600	CAACGGCTTGGACTGGTTCG	TP309	Failed
Os03g0409100	TGGAACTTGCAGCGTCTTGA	Kasalath	Failed
Os03g0607200	GGAGTGCAACTGCGTGCCCA	Wuyunjing24	Homozygous:+2bp<br>Homozygous:+2bp<br>Biallelic:+2bp(AT/CG)

Os03g0609200	CCTGAACATCACCAACCTAA	Kasalath	Failed
Os03g0610900	CGACGGCGACCGGTACGAGC	TP309	Homozygous:-1bp
Os03g0729500	TCTTTCCAGCTGGGCTACTC	Wuyunjing24	Failed
Os03g0729500	TCTTTCCAGCTGGGCTACTC	Wuyunjing24	Failed
Os03g0795900	CCGTCGACGGGCGTGGCGAA	Kasalath	Heterozygous:-6bp/WT
Os03g0805600	CGAGAAGAACTTCTATTTCC	TP309	Biallelic:+1bp/-6bp<br>Homozygous:+1bp<br>Homozygous:-1bp
Os03g0810600	GCGACAGCGGCGAACCTGGC	TP309	Failed
Os03g0821800	GCTGGAGAGGACTGAAGTCT	Kasalath	Heterozygous:-1bp/WT
Os03g0822000	GCGGATCCCGGGGGCGTACG	Kasalath	Heterozygous:-1bp/WT<br>Homozygous:-3bp<br>Biallelic:-3bp/-10bp<br>Homozygous:-1bp<br>Biallelic:-31bp/+1bp
Os03g0828100	GCGACCGTGTCCGGGAGCCT	TP309	Heterozygous:+1bp/WT
Os03g0828500	TAGTTCAAAGACCTCCACCC	TP309	Failed
Os03g0833300	GCGCAGAGCCGCCACCTGTA	Wuyunjing24	Biallelic:-2bp/+1bp
Os03g0859900	GCAGAAGAACAGTGCCCGCG	Kasalath	Heterozygous:-1bp/-2bp
Os03g0861300	CTAGTGGACACCGGCGAGCT	TP309	Homozygous:-4bp
Os04g0391000	GAACTGCTCGAAGCACCGCC	TP309	Homozygous:+6bp
Os04g0432000	CGCCATGAAGTACATCCCTC	TP309	Biallelic:+1bp(C/A)<br>Homozygous:+1bp
Os04g0551500	CACCGGCCGTTTCGGAGCCC	Wuyunjing24	Homozygous:+1bp
Os04g0608100	GGTAGTGGAGATTAGTGGAA	TP309	Heterozygous:-4bp/WT<br>Biallelic:-4bp/+1bp<br>Biallelic:+1bp/-5bp<br>Homozygous:+1bp<br>Biallelic:-1bp/-6bp<br>Homozygous:-3bp<br>Biallelic:-3bp/-21bp<br>Homozygous:-1bp<br>Homozygous:-6bp<br>Homozygous:-5bp
Os04g0608500	TGGCAAATACTCAGGTAATC	Kasalath	Failed
Os05g0112200	CCTTCCTGGATTCCTCGAGC	TP309	Heterozygous:-4bp/WT<br>Heterozygous:+1bp/WT<br>Homozygous:-4bp<br>Homozygous:+1bp
Os05g0169800	TTGTTGTCACCGTACAGCTG	Wuyunjing24	Homozygous:-3bp<br>Homozygous:-1bp<br>Biallelic:-3bp/-1bp
Os05g0170000	TACCAGAGTTCTGGCGGGAC	Wuyunjing24	Homozygous:+1bp

Os05g0170000	TACCAGAGTTCTGGCGGGAC	Kasalath	Homozygous:-20bp<br>Homozygous:-1bp<br>Biallelic:-1bp/-5bp<br>Homozygous:-5bp<br>Homozygous:-22bp<br>Heterozygous:-20bp/WT
Os05g0375532	AGTGTCTCGTGCGATTTAGC	Kasalath	Biallelic:+1bp/-6bp<br>Homozygous:-6bp
Os05g0417100	GCGGCAGGATGCCTCGGGCT	TP309	Heterozygous:-1bp/WT<br>Homozygous:-1bp<br>Homozygous:-3bp<br>Homozygous:+1bp<br>Biallelic:+1bp/-1bp<br>Homozygous:-54bp<br>Biallelic:+1bp/-1bp<br>Heterozygous:-54bp/WT
Os05g0417200	CGCCGAACAGGTCGCGGGCA	TP309	Failed
Os05g0418100	GTTCACGCCGACGTGGATCG	TP309	Homozygous:+1bp<br>Homozygous:-3bp<br>Biallelic:+1bp/-3bp
Os05g0467000	CGGCCATGGGTAACTGCTGC	TP309	Biallelic:+1bp(T/C)<br>Heterozygous:-1bp/WT<br>Homozygous:+1bp
Os05g0489800	CTTCAAATATGTCCACTAAG	TP309	Heterozygous:-6bp/WT
Os05g0513100	AACAGCACGAATGAGGAGCT	Wuyunjing24	Biallelic:+1bp(A/C)<br>Heterozygous:-3bp/WT<br>Biallelic:+1bp/-3bp<br>Heterozygous:+1bp/WT<br>Heterozygous:-1bp/WT
Os05g0513100	AACAGCACGAATGAGGAGCT	Kasalath	Failed
Os05g0571200	CGGCGGCGGGAAGCAGGACG	Kasalath	Homozygous:-3bp<br>Homozygous:-27bp<br>Biallelic:-3bp/-5bp
Os05g0571700	TTGGGTCTGAACACTTCGCT	Wuyunjing24	Homozygous:+1bp
Os05g0573200	GAATACTCGAGTCATCTCAT	Wuyunjing24	Failed
Os05g0573500	GGCCGATGTTGGCGATCGGC	Kasalath	Failed
Os06g0173100	GCCTACTCGACAACGAGATC	Kasalath	Failed
Os06g0204800	GCCTGTTCACGGCGACGCAG	Wuyunjing24	Homozygous:-9bp
Os06g0275700	TCGCCAAGGACCTCGTCATG	TP309	Heterozygous:+1bp/WT
Os06g0284900	GGCAGCTGCCACCCCTGCAA	TP309	Homozygous:+1bp
Os06g0284900	GGCAGCTGCCACCCCTGCAA	Kasalath	Biallelic:+1bp/+1bp
Os06g0498800	GCAGCCGTTGGTGAGGTCCT	TP309	Homozygous:+2bp
Os06g0552900	CTCTAACTGTGATGTATGAT	TP309	Homozygous:-4bp
Os06g0571100	TCGTCCTTGGTCGCTTCCAA	Kasalath	Homozygous:RPL:-<br>4bp(TTCCA→G)<br>Heterozygous:+1bp/WT
Os06g0597000	GGCCGCCGGTGAGGGCGTAC	Wuyunjing24	Homozygous:+1bp<br>Biallelic:+1bp(T/G)
Os06g0597000	GGCCGCCGGTGAGGGCGTAC	Kasalath	Homozygous:-39bp<br>Biallelic:-3bp/-39bp

Os06g0597400	CTCGGTGAAGCCATTGCCTG	Wuyunjing24	Homozygous:+1bp<br>Homozygous:-6bp
Os06g0610300	TGACACAGCTAGACCAGGTG	TP309	Homozygous:-1bp
Os06g0663500	GATCAAACTCCGAAGGCGCG	Wuyunjing24	Biallelic:-<br>28bp/RPL:A→C
Os06g0703500	AGAGCCCTGTGTTTTGGGAC	Wuyunjing24	Failed
Os06g0703500	AGAGCCCTGTGTTTTGGGAC	TP309	Homozygous:+1bp
Os07g0176200	GGATACTCCTGACCGTGCTG	TP309	Homozygous:+1bp
Os07g0505300	GCGAGGCTGGGCGGTACAAC	Kasalath	Failed
Os07g0586700	TGTCGGGCCGTCGGACTGGG	TP309	Homozygous:-6bp<br>Homozygous:+1bp
Os07g0605200	GACGTCGGCGTCACAGAGCA	Kasalath	Homozygous:-4bp<br>Homozygous:-2bp<br>Biallelic:-4bp/-2bp
Os07g0609000	GCTCTCCCTGGCGAAGTTGA	Kasalath	Homozygous:-2bp<br>Biallelic:-2bp/-3bp<br>Heterozygous:-2bp/WT
Os07g0609000	GCTCTCCCTGGCGAAGTTGA	Wuyunjing24	Homozygous:+1bp
Os07g0619400	GGTAGAGGTTGACCCACGCC	Kasalath	Failed
Os08g0427500	ACCCCTAGAGCGTCTTGAAT	TP309	Heterozygous:+1bp/WT<br>Heterozygous:-3bp/WT<br>Heterozygous:-4bp/WT
Os08g0502700	CGGAACCACCTGTTCGTGCC	Kasalath	Failed
Os08g0531600	AGTGCCGCGACTACCATCGC	Wuyunjing24	Homozygous:-1bp
Os08g0531600	AGTGCCGCGACTACCATCGC	Kasalath	Heterozygous:-2bp/WT
Os08g0566400	ATGACGATGGTACGCATCAT	Wuyunjing24	Heterozygous:+1bp/WT
Os09g0401300	AGGGACTTCCTGGGCGCGAT	Wuyunjing24	Failed
Os09g0408600	GTCGAGCCGACCACCAGAGG	Kasalath	Failed
Os09g0439200	GGAGGTGGAGCCAAACACGG	TP309	Failed
Os09g0439200	GGAGGTGGAGCCAAACACGG	Kasalath	Heterozygous:+1bp/WT
Os09g0475800	CGTGGACCTCCGCCGGCCGA	TP309	Failed
Os09g0491532	GACGACGACGATGGGCTCCT	Wuyunjing24	Homozygous:-1bp
Os09g0507100	GCCGCCACCGCGGCAATGCC	Kasalath	Homozygous:-1bp
Os09g0526600	CGACGTGATCTCGTGGAACG	Wuyunjing24	Failed
Os10g0341700	TGCAGGAGAGGGTGCCCATC	Wuyunjing24	Heterozygous:-1bp/WT<br>Homozygous:-1bp<br>Homozygous:-5bp<br>Biallelic:-5bp/-3bp<br>Homozygous:-3bp
Os10g0341700	TGCAGGAGAGGGTGCCCATC	Kasalath	Homozygous:-1bp<br>Homozygous:-5bp<br>Biallelic:-5bp/-3bp<br>Homozygous:-3bp<br>Biallelic:-1bp/-3bp
Os10g0342300	TCCGTAGTGGCATAGCTCGT	Kasalath	Homozygous:-3bp

Os10g0342300	TCCGTAGTGGCATAGCTCGT	Wuyunjing24	Homozygous:-1bp<br>Homozygous:+1bp<br>Biallelic:+1bp/-1bp
Os10g0392400	GCCTCCTCAGCCAGTACATG	Kasalath	Failed
Os10g0406300	GCTGTTCGGGAAGACGATCC	TP309	Homozygous:+1bp
Os10g0463400	CACCGAGAGCTGTGGCCTTA	Kasalath	Homozygous:-7bp<br>Homozygous:-8bp<br>Biallelic:-7bp/-8bp
Os10g0471100	AGCGGTAAGTGTTGCCCGCC	Kasalath	Biallelic:-1bp/-4bp<br>Biallelic:+1bp(A/C)<br>Homozygous:-1bp<br>Biallelic:-1bp/-22bp<br>Homozygous:-22bp
Os10g0471700	CAGGGGTGCTAATCGTATCG	Wuyunjing24, Kasalath	Failed
Os10g0487300	TTGGAATTCAGTAAGATACG	TP309	Heterozygous:-1bp/WT
Os10g0497700	ATCTGGGTGATATCGAGGAT	TP309	Homozygous:+1bp<br>Homozygous: -19bp<br>Homozygous:-9bp
Os10g0555100	CGACGCGCTCATAGTCAACC	Kasalath	Biallelic:+1bp/-4bp<br>Biallelic:-1bp/-4bp<br>Homozygous:-4bp<br>Homozygous:+1bp
Os10g0555700	TCCGTGATGATCACCGTCCT	Kasalath	Homozygous:+1bp<br>Biallelic:+2bp-1bp<br>Homozygous:-15bp
Os10g0556000	AGAACGGTAGACGGCCATGG	Kasalath	Biallelic:+1bp/-2bp<br>Homozygous:-2bp<br>Homozygous:+1bp
Os10g0558900	ACTGTTCTACTATCCGTGCC	Kasalath	Homozygous:+1bp
Os10g0559300	CCTCGTCACACGGCCAGTTG	Kasalath	Homozygous:+1bp
Os10g0559700	CCAGTAGTCAAGACTTTCGG	Kasalath	Homozygous:-3bp<br>Homozygous:-1bp<br>Biallelic:-1bp/-3bp<br>Heterozygous:-3bp/WT<br>Heterozygous:-1bp/WT
Os10g0559800	AAAGAACTACTTCCTGAATA	Kasalath	Homozygous:-10bp
Os10g0560400	CTCGGCGTAGGCCTTGCGCG	Kasalath	Homozygous:+1bp<br>Homozygous:+1bp<br>Biallelic:+1bp(T/C)
Os10g0567400	CTGGAGCAACACCTGAAGGA	TP309	Biallelic:+1bp/-19bp
Os10g0578500	CGGTGGGTGGGACCTGGGCC	Wuyunjing24	Biallelic:-1bp/+1bp<br>Homozygous:-1bp<br>Biallelic:-4bp/-5bp<br>Biallelic:-5bp/-6bp

Os11g0247300	CAAGATCGACGAAGACAGCA	Kasalath	Homozygous:-1bp
Os12g0168800	GTCGAGGTGGGCGATGAGGA	Kasalath	Failed
Os12g0168800	GTCGAGGTGGGCGATGAGGA	Wuyunjing24	Heterozygous:-1bp/WT<br>Heterozygous:+1bp/WT
Os12g0611000	AACATTCTGCTCTACCCGAG	Kasalath	Heterozygous:-2bp/WT<br>Biallelic:+1bp/-1bp

[a] Genotypes of mutants are given as "Mutation type": changed bases. Heterozygous (only one allele mutated) and bi-allelic (both alleles mutated but with differed mutations) mutants are indicated with "/". Types of mutation: RPL - replacement, including base substitutions. [b] A chimeric mutant with five possible mutation alleles.
