# Supplementary material for: In rice splice variants that restore the reading frame after frameshifting indel introduction are common, often induced by the indels and sometimes lead to organism-level rescue
Source: PLoS Genet. 2022 Feb 18;18(2):e1010071. doi: 10.1371/journal.pgen.1010071 (PMC8893660; doi:10.1371/journal.pgen.1010071)
Supplement: S2 Table — (PDF) [file pgen.1010071.s016.pdf]

S2 Table. Primers for cDNA amplification in mutants.

Gene locus	Primer-Forward	Primer-Reverse
Os01g0277500	CTTAGGCAGGGCGACGA	GCGAAAATGCTGCTGGAT
Os01g0302500	GTTCTCTCCAGCTTACAGC	TCCTCGGCCTATCTAACAG
Os01g0616900	CGTCGTCCTCAAGTCGTG	CTGCCTAACATCAATATCCTC
Os01g0678700	GTGGTGCCAGTTTTGAGTTT	GCCAACCTGCTCATTCGC
Os01g0758200	CTGTTCGGCAAGGTCATC	TACATTACACCCGCAACC
Os01g0884300	ACGAGGAGCTGGTGATGC	CCTGAGGCTGTTCTTCTTG
Os01g0884300	TCTAATCCAATCCAATCCACT	GGATAAAGCCACAGAGAGAG
Os01g0884350	TCGGGTACTTGCGGTCTC	GGAGAACGGACGATCACC
Os01g0884400	TCCTGCGACGAAGCGAAG	GACATCAATCATTTGGAATCAC
Os01g0885000	GGGAGGCAATCTGGTACA	ATGCTGTGGCGTTCTTCA
Os01g0909100	ATGGGATTCTGGGGAGTTG	CATCCGAACTATCACCATCA
Os01g0919900	GCCACCCGCATCGCCATC	AACACACGAGCAGATTGACACG
Os01g0922600	AGATGCCTTCCTGGGACCT	ACCACGATGAGAAAGGAAGAG
Os01g0922600	ATGGATTGGGACGCCAAG	AATCAGTAATCCTCCAGCTTTT
Os01g0930800	ACCACAACCACACGTACTC	ATCTGTATTTCGTGTGCAGG
Os01g0930800	CACGTACTCACTCGCATGG	TGTATTTCGTGTGCAGGAGC
Os02g0125600	ACTTTATGCTGATCCTCCTC	TCACGGGAAGGAGTTGCTG
Os02g0174100	GATGCCTCCTCCGAAGCC	CAGTTCGGATAAAAAGGACA
Os02g0252400	GGTAAACGAGCGACGATG	TCGTCTCGGTTCACTCCT
Os02g0259600	ATAGCCCACACTCTCATCCAC	TTCCAGACAAGAAAAGGTAGCA
Os02g0312600	CACCAAACCCAACCAAGAAC	CTGCAACACACAAACATAACAC
Os02g0529400	CATTTGAGTTGGGAGCACG	GTCATGTCAAATGGAGTATATGGTA
Os02g0553200	CGCATCGCCGCCTCCCT	GCCAGCCCTCCGATCAAG
Os02g0606200	GCTGCGACGTGTGCCAGGA	TTCAACCAAGATCAGGGACGA
Os02g0606200	CGGCGGCGGTGGTGTGCT	TTCAACCAAGATCAGGGACGA
Os02g0606200	AACGTCGATCACAAATTCAC	ATTCCATAGGGTTCGTGATG
Os02g0678800	ATGGATGAGGAGAAGGAAGC	GAGGACGCTGGTGGGGGA
Os02g0707200	GACTCCGACTGGCTCAAG	ATGCCGCTCCACAGTCC
Os02g0726300	ACCAAGATAGCGGGATGA	GCTTTCTTGGACAGTTACAC
Os02g0777400	GGCACAAGTTCCCAAAGATG	ATCACCTCGCCGAACTTCA
Os03g0255400	ATGGAAGGTGTGGTAACCAG	TTATAGGCATGTACAGCGTGA
Os03g0276300	ATGGTGTTCCCTTCAGTG	CCTGATGACGAATGGAAT
Os03g0297400	GCCATCCTCCACGCCTACAA	GCCAGTTTGCCACAACAGCC
Os03g0607200	CCACGACTTTAGCCATCTT	TACGGCATATCCCCTGTT
Os03g0610900	CGCCGTCAAGTACATCG	TATCTCCCCACTGCTTTCC
Os03g0805600	GATGCCCTCTCCCTCCT	CACTTGTAAGCATGGACGTA
Os03g0821800	GGCTGGCCGCTCTCCTCCT	TTGTCAGCGTACCGTGCATATG
Os03g0821800	GGCTGGCCGCTCTCCTCCT	ATCCAGGTATTTCAGTCCAGTT
Os03g0828100	TAGAGAGCAGCGTAGTCG	GACAAACTCAATAACTGG
Os03g0833300	CTGTACGGCGGAGGGCTA	TCACATTGGTCCACGTTCTA
Os03g0859900	TACAGAGCAGAAGAACAGTG	TGAAAGTCGTAGTCGTATGG
Os04g0432000	GCTGAGACGACGAGAGGAA	GCTGCCACATTATGACAATATC
Os04g0608100	CGAGCAAAGGTAGTGGAG	TCCCGCATTCATAGTTCA

Os05g0112200	TGCCTTCCTGGATTCCT	CTTGGGTAGCCTTCTCTC
Os05g0170000	CGCGAAATCGGAGATTCC	ATTGCCTTCGATGGAATGAC
Os05g0375532	CACCGTCCAGGACAGAGTTCAA	ACCGCCGCCTCCTACAAG
Os05g0417100	CGCCCTTCCAAACCCTA	CACCATTAGTCGCCTCCTC
Os05g0418100	CGGGAAGGGAGGGAAGATG	TCAGTCACCACAGCTTAGGCAA
Os05g0467000	CGAGGGAGGATGTGAAGAC	GCTCTGCCTATCTAAATTACC
Os05g0467000	CAGAGCCCAATGCGGAACA	GGTCTCTGCTTAGTGGGTCC
Os05g0513100	ATGACAAGCAATAACAGCACG	TTGGAGTAGGGATTATTGTCT
Os05g0571700	CGACTGATTGCGTTTCCT	CCACACGCACATATTTGAC
Os06g0275700	GCAGAGAGTGCAGAATTCGA	ACCAAATACACCAAGCCCA
Os06g0552900	CATTGACAAGGAGCCATATAG	TTGCCGAGGTTGAAACG
Os06g0571100	GCAGCGATTCGTCTCCCTC	TCAGGCTAAGCACTCGTTCAG
Os06g0597000	CCACACCCCTCGTCTCC	TATCTGGATGATCGTCTTGG
Os06g0597000	CTCGGGCCTCGACTACG	TATCTGGATGATCGTCTTGG
Os06g0663500	ATGGAGTGCAACCCCGTC	AAACATTGAAGACTCGTAGGA
Os06g0663500	ATGGAGTGCAACCCCGTC	TCAATGTATCTGGTTCAGACCA
Os06g0703500	AGAAGAGCCCTGTGTTTTG	GCCGGAGCTGCTGCCATT
Os07g0176200	TGGTGGTGAGAGCGAGAG	TGCTTCAGATGTGAGGCAC
Os07g0176200	TGGGAAGATGTTGTCTGCTG	GGATGGACCTAAAGCAAGAG
Os07g0605200	AGATAAACAGGCAGGTGAC	CTTGGTTGGCTTTGACTG
Os07g0609000	CCATGATTTGCCTCCGAGC	TCCGACGACGAATTCCAGC
Os08g0427500	CGACGATCAAGCCACGAGG	AGAGTTCACCTGGGACCAAA
Os09g0439200	GGCGGGGAAGGACAGGTC	TATCTCCTGCTTTATTGTCATC
Os09g0491532	GTCCACGGGCTCAAGTTC	GTTCATGGCATTGGTGGAGT
Os09g0507100	ATGCCTGTTTCTTGGGACCT	AATGGGAGCGACGGCGAC
Os10g0341700	AGAGAGCGATAAAACTGTGG	TTAATTTCTTTTGCAGAAGAGC
Os10g0342300	ATGTCTGTCCTTCTTGGCTT	CAGTGGTGTTGAATGTGAGC
Os10g0406300	TGTTCAGCAGCATAGTGG	GATGTTTCGCCGTCTTG
Os10g0463400	ATGGATCACCGAGAGCTG	TGGTCCATATTCCTGTTTGT
Os10g0471100	ATGGCCACAAACCCAGGAC	ACCTTGAGCAACTGGGCAG
Os10g0471100	ATGGCCACAAACCCAGGAC	TTACGTACATGTGATGGGCT
Os10g0487300	GCGATGTCATTGTTCAAACG	TATGCGATTGATTTCTCTGGTC
Os10g0497700	ATGGCGATTGGTGTTGGT	TTCGGCAGCCACAAGAG
Os10g0497700	GTAGCGGCGAAGGATATAGC	GCCCTTCCCTTGTTATTGTTA
Os10g0555100	GTGGGGGGTGGTGGTCGTC	CACCAAGCCGAGGGAAGAAC
Os10g0555700	ATGCTGCTCTCCGTGCTC	TGACCTGGTGGGCGATGA
Os10g0556000	GCTTTTTCCATCTCCAAGAA	TTAGCTGCTGTACTGGACGA
Os10g0559800	ATGGAAGGGACGGTGCA	TCAGAAGAGTGGCTTGTCGA
Os10g0567400	ACTGTGGCATCGCTGTCT	ACTGAATGGCAACCTGTCT
Os10g0567400	TACCCAGTTGCTTTCTCC	TACTGAATGGCAACCTGTC
Os10g0567400	TGACCTTGCTGATTGAAAC	CTGAATGGCAACCTGTCT
Os10g0578500	CTGCCTCCTGCTTCTATGC	TGCAAGCAGATCATTAGTTGAG
Os12g0168800	GGTGGAGGGCGGAATAGG	GAACATCCATCCCCCTTG
Os12g0611000	TGGAACATAAAACAAGGTGC	ATTCCGCTAGATCGTTGGC
