# Supplementary material for: In rice splice variants that restore the reading frame after frameshifting indel introduction are common, often induced by the indels and sometimes lead to organism-level rescue
Source: PLoS Genet. 2022 Feb 18;18(2):e1010071. doi: 10.1371/journal.pgen.1010071 (PMC8893660; doi:10.1371/journal.pgen.1010071)
Supplement: S3 Table — (PDF) [file pgen.1010071.s017.pdf]

S3 Table. Statistics of successfully amplified CRISPR/Cas9 mutant gene loci and corresponding junctions.

Gene Locus	Gene symbol	Genotypes of mutants [a]	Mutation type	No. of novel junctions	No. of novel 3n junctions	No. of novel non-3n junctions	No. of novel junctions with potential rescue function
Os01g0302500	*OsH6*	DEL:11167424-11167428/IN:11167425-11167426:T<br>DEL:11167424-11167428	Biallelic<br>Homozygous	5	2	3	2
Os01g0277500	*Dof4*	DEL:9734008-9734007	Homozygous	60	15	45	0
Os01g0616900	-	IN:24504763-24504764:T/WT<br>IN:24504763-24504764:T	Heterozygous<br>Homozygous	174	58	116	0
Os01g0678700	*OsDP1*	IN:27928349-27928350:G	Homozygous	15	6	9	0
Os01g0758200	*Dof6*	DEL:31851484	Homozygous	38	22	16	3
Os01g0884300	*NAC6*	IN:38401362-38401363:T	Homozygous	19	9	10	0
Os01g0884350	-	IN:38401403-38401404:G	Homozygous	6	1	5	0
Os01g0884400	*PUB16*	IN:38412021-38412022:A/DEL:38412022-38412024<br>DEL:38412022-38412024	Biallelic<br>Homozygous	0	0	0	0
Os01g0885000	-	IN:38443793-38443794:G<br>IN:38443793-38443794:G/WT	Homozygous<br>Heterozygous	2	1	1	0
Os01g0909100	*HDT2*	DEL:39589406/RPL:39589409-39589410:CC→T<br>DEL:395893403-39589406/DEL:39589406<br>DEL:395893403-39589418	Biallelic<br>Biallelic<br>Homozygous	69	25	44	0
Os01g0919900	*OsSSI2*	DEL:40148409-40148415	Homozygous	85	28	57	9
Os01g0922600	*SPL2*	IN:40332272-40332273:T/DEL:40332273-40332274	Biallelic	42	21	21	2
Os01g0930800	*BGLU5*	IN:40842390-40842391:G<br>IN:40842390-40842391:G/DEL:40842391-40842392<br>DEL:40842391-40842392	Homozygous<br>Biallelic<br>Homozygous	38	12	26	1
Os02g0125600	*OsNIN5*	IN:1343497-1343498:A	Homozygous	15	7	8	4
Os02g0174100	*SPL4*	DEL:4074223-4074224	Homozygous	2		2	1

Os02g0252400	*RPBF*	IN:8591730-8591731:A/IN:8591730-8591731:C<br>IN:8591730-8591731:A	Biallelic<br>Homozygous	40	14	26	15
Os02g0259600	*RPL21*	IN:9005601-9005602:T	Homozygous	19	9	10	0
Os02g0312600	*RAC7*	DEL:12326450/DEL:12326441-12326449	Biallelic	41	13	28	4
Os02g0529400	*NIN3*	IN:19438139-19438140:T<br>IN:19438139-19438140:T/WT<br>IN:19438140-19438141:A/WT<br>RPL:19438138-19438140:ATG→GCGA/DEL:19438140-19438142	Homozygous<br>Heterozygous<br>Heterozygous<br>Biallelic	320	121	199	3
Os02g0553200	*APX8*	DEL:20871237	Homozygous	64	27	37	11
Os02g0606200	*OsBBX4*	DEL:23759482<br>IN:23759485-23759486:A<br>DEL:23759482/WT<br>IN:23759485-23759486:A/WT	Homozygous<br>Homozygous<br>Heterozygous<br>Heterozygous	57	42	15	20
Os02g0678800	*GRF10*	DEL:27728013/WT	Heterozygous	6	4	2	0
Os02g0707200	*Dof7*	IN:29239255-29239256:A	Homozygous	35	14	21	2
Os02g0726300	*Dof4*	IN:30208083-30208084:T/DEL:30208084	Biallelic	20	9	11	0
Os02g0777400	*RLCK85*	IN:32887558-32887559:A/WT	Heterozygous	19	3	16	0
Os03g0255400	-	DEL:8214189-8214192/WT<br>DEL:8214190/WT	Heterozygous<br>Heterozygous	16	6	10	0
Os03g0276300	*Dof14*	DEL:9360529-9360530/DEL:9360530-9360531/<br>DEL:9360529/IN:9360528-9360529:A/WT [b]	Chimeric	32	10	22	10
Os03g0297400	-	DEL:10404085-10404112/WT<br>IN:10404107-10404108:A	Heterozygous<br>Homozygous	97	36	61	1
Os03g0607200	-	IN:22817384-22817385:AT<br>IN:22817384-22817385:CG	Homozygous<br>Homozygous	13	4	9	3
Os03g0610900	*SAPK10*	DEL:23071007	Homozygous	17	7	10	0
Os03g0805600	-	DEL:33640879<br>IN:33640877-33640878:T	Homozygous<br>Homozygous	32	20	12	0
Os03g0821800	-	DEL:34500810/WT	Heterozygous	32	8	24	1
Os03g0828100	-	IN:34785590-34785591:A/WT	Heterozygous	14	6	8	2
Os03g0833300	*SPL6*	DEL:35015181-35015182/IN:35015180-35015181:T	Biallelic	32	11	21	0
Os03g0859900	-	DEL:36292529/DEL:36292529-36292530	Biallelic	13	7	6	0

Os04g0432000	*SAPK7*	IN:21414844-21414845:A<br>IN:21414844-21414845:T	Homozygous<br>Homozygous	69	26	43	9
Os04g0608100	*GalK2*	DEL:30769370-30769374<br>IN:30769369-30769370:T/DEL:30769370-30769373<br>DEL:30769370-30769374/IN:30769369-30769370:T	Homozygous<br>Biallelic<br>Biallelic	9	2	7	0
Os05g0112200	*Dof19*	IN:660001-660002:A	Homozygous	27	12	15	0
Os05g0170000	*BC10*	DEL:4197198<br>DEL:4197199-4197203<br>DEL:4197189-4197208/ DEL:4197198	Homozygous<br>Homozygous<br>Heterozygous	48	14	34	3
Os05g0375532	-	IN:18127156-18127157:A/DEL:18127157-18127162	Biallelic	13	7	6	0
Os05g0417100	*TCM5*	DEL:20433587/IN:20433586-20433587:A<br>DEL:20433569-20433622<br>DEL:20433587/WT<br>IN:20433586-20433587:A	Biallelic<br>Homozygous<br>Heterozygous<br>Homozygous	8	2	6	0
Os05g0418100	*MLO9*	IN:20490025-20490026:G	Homozygous	22	7	15	10
Os05g0467000	*CDPK16*	IN:22924235-22924236:T/IN:22924235-22924236:C<br>IN:22924235-22924236:T	Biallelic<br>Homozygous	40	9	31	6
Os05g0513100	*TCP18*	IN:25437388-25437389:A/DEL:25437388-25437390<br>IN:25437389-25437390:A/WT	Biallelic<br>Heterozygous	4	4	0	0
Os05g0571700	*OsFbox282*	IN:28492567-28492568:A	Homozygous	159	55	104	14
Os06g0275700	*OsPUB22*	IN:9371855-9371856:G/WT	Heterozygous	5	2	3	0
Os06g0552900	*FTL12*	DEL:20974074-20974077	Homozygous	13	7	6	1
Os06g0571100	*HDAC6*	RPL:22126726-22126730:TTCCA→G<br>IN:22126728-22126729:G/WT	Homozygous<br>Heterozygous	92	34	58	9
Os06g0597000	*IAA23*	IN:23502386-23502387:G<br>IN:23502386-23502387:T/IN:23502386-23502387:G	Homozygous<br>Biallelic	63	31	32	27
Os06g0663500	*SPL11*	DEL:27390017-27390044/RPL:27390030:A→C	Biallelic	17	5	12	0
Os06g0703500	*SPL12*	IN:29707026-29707027:G	Homozygous	35	10	25	0

Os07g0176200	-	IN:4020484-4020485:A<br>IN:4020484-4020485:T	Homozygous<br>Homozygous	26	7	19	0
Os07g0605200	*MADS18*	DEL:24788676-24788677<br>DEL:24788676-24788677/DEL:24788678-24788681	Homozygous<br>Biallelic	31	9	22	1
Os07g0609000	-	DEL:25050050-25050051<br>DEL:25050050-25050052/DEL:25050050-25050051	Homozygous<br>Biallelic	8	5	3	2
Os08g0427500	*XPC*	IN:20563973-20563974:C/WT<br>DEL:20563970-20563973/WT	Heterozygous<br>Heterozygous	308	110	198	14
Os09g0439200	*TIFY10C*	IN:16274854-16274855:A/WT	Heterozygous	2	1	1	0
Os09g0491532	*SPL17*	DEL:18921348	Homozygous	21	6	15	3
Os09g0507100	*SPL18*	DEL:19643558	Homozygous	44	17	27	4
Os10g0341700	*CSLH1*	DEL:10089601<br>DEL:10089601/WT	Homozygous<br>Heterozygous	69	20	49	5
Os10g0342300	-	IN:10122849-10122850:A	Homozygous	1	0	1	0
Os10g0406300	*RDD3*	IN:13884990-13884991:T	Homozygous	62	23	39	0
Os10g0463400	*EF1*	DEL:17081319-17081326<br>DEL:17081323-17081329/DEL:17081319-17081326	Homozygous<br>Biallelic	1	1	0	0
Os10g0471100	*WDA1*	DEL:17453731<br>DEL:17453722-17453743/DEL:17453731<br>DEL:17453731-17453734/DEL:17453731<br>DEL:17453722-17453743	Homozygous<br>Biallelic<br>Biallelic<br>Homozygous	274	67	207	13
Os10g0487300	*NBS1*	DEL:18436775/WT	Heterozygous	19	4	15	2
Os10g0497700	*BC1L9*	IN:18978374-18978375:A<br>DEL:18978375-18978383	Homozygous<br>Homozygous	29	11	18	1
Os10g0555100	*GGT*	DEL:21800369-21800372<br>IN:21800369-21800370:T	Homozygous<br>Homozygous	10	4	6	2
Os10g0555700	*EXPB2*	IN:21836821-21836822:T	Homozygous	3	1	2	1
Os10g0556000	-	DEL:21848261-21848262<br>DEL:21848261-21848262/IN:21848262-21848263:G	Homozygous<br>Biallelic	4	2	2	0
Os10g0559800	-	DEL:22041473-22041482	Homozygous	8	4	4	0
Os10g0567400	*YGL10*	IN:22486317-22486318:T/DEL:22486317-22486335	Biallelic	46	16	30	4

Os10g0578500	-	DEL:23071574<br>DEL:23071571-23071575/DEL:23071572-23071575	Homozygous<br>Biallelic	11	4	7	6
Os12g0168800	*GATA26*	IN:3496386-3496387:T	Homozygous	4	2	2	0
Os12g0611000	*OsLSD1*	DEL:25818248/IN:25818246-25818247:T	Biallelic	29	10	19	0
**Total Junction**				**3123**	**1129**	**1994**	**231**

[a] DEL – deletion, IN - insertion, WT - wild type allele, "genomic start – genomic end"- the mutation site. [b] A chimeric mutant with five possible mutation alleles.
