# Supplementary material for: In rice splice variants that restore the reading frame after frameshifting indel introduction are common, often induced by the indels and sometimes lead to organism-level rescue
Source: PLoS Genet. 2022 Feb 18;18(2):e1010071. doi: 10.1371/journal.pgen.1010071 (PMC8893660; doi:10.1371/journal.pgen.1010071)
Supplement: S4 Table — (PDF) [file pgen.1010071.s018.pdf]

S4 Table. The relative level of rescue form of the two alleles (*WT* vs *mutant*) in the wt/indel heterozygotes[a]

Gene Locus	Mutation	Rescue junction	R_w	Heterozygote		R_{mw} (R_m-*mutant allele*/ R_w)
				R_w-*wt allele*[b]	R_m-*mutant allele*[c]	
Os03g0821800	DEL:34500810	chr03:34500398-34500769	0.07%	0.04%(4/10281)[d]	0.11%(4/3551)	1.67
Os03g0828100	IN:34785590-34785591:A	chr03:34785732-34786258	NA[e]	0.38%(1581/415193)	13.81%(62/449)	NA
Os06g0571100(*HDAC6*)	IN:22126728-22126729:G	chr06:22126287-22126486	0.01%	0.00%(4/111386)	0.03%(3/9809)	0.4
Os06g0571100(*HDAC6*)	IN:22126728-22126729:G	chr06:22126600-22126702	0.09%	0.32%(603/187775)	0.84%(140/16631)	3.6
Os08g0427500(*XPC*)	IN:20563973-20563974:C	chr08:20563978-20564176	NA	0.00%(2/97317)	0.01%(10/70481)	NA

[a] Only cases where the linkage of rescue junction and the wt/indel is distinguishable were investigated here. [b] the relative level of the rescue form of the WT allele in WT/indel heterozygotes; [c] the relative level of rescue form of the mutant allele in heterozygotes; [d] depth of supporting read-pairs for wt or indel in wt/indel heterozygotes; [e] no RT-PCR-data of WT available.
