# Supplementary material for: In rice splice variants that restore the reading frame after frameshifting indel introduction are common, often induced by the indels and sometimes lead to organism-level rescue
Source: PLoS Genet. 2022 Feb 18;18(2):e1010071. doi: 10.1371/journal.pgen.1010071 (PMC8893660; doi:10.1371/journal.pgen.1010071)
Supplement: S5 Table — (PDF) [file pgen.1010071.s019.pdf]

S5 Table. The genes potentially rescued by exon skipping.

Gene Locus	Rescue junction	Indel size	Distance of indel to nearest splice site	R_m	R_w	R_{mw}
Os02g0553200(*APX8*)	chr02:20868768-20871877	DEL:1 bp	48 bp	0.002%	0	-[b]
	chr02:20870729-20871614	DEL:1 bp	48 bp	0.001%	0	-
	chr02:20870729-20871877	DEL:1 bp	48 bp	14.894%	0.840%	17.7
Os06g0597000(*IAA23*)	chr06:23502267-23502763	INS:1 bp	33 bp	0.002%	0.0002%	7.5
Os08g0427500(*XPC*)	chr08:20561180-20564433	INS:1 bp	77 bp	0.030%	0[a]	-
Os05g0170000(*BC10*)	chr05:4196904-4198079	DEL:20 bp	8 bp	52.53%	0.002%	23769.4

[a] RNA-seq data are used when RT-PCR-seq is not available; [b] denominator is 0
