# Supplementary material for: In rice splice variants that restore the reading frame after frameshifting indel introduction are common, often induced by the indels and sometimes lead to organism-level rescue
Source: PLoS Genet. 2022 Feb 18;18(2):e1010071. doi: 10.1371/journal.pgen.1010071 (PMC8893660; doi:10.1371/journal.pgen.1010071)
Supplement: S6 Table — (PDF) [file pgen.1010071.s020.pdf]

S6 Table. Mutants with high (junction reads/ junction region depth＞10%) relative levels of expression of rescue junctions.

**Gene** Locus	**Mutant type**	**Genotype**	**Potential rescue junction**	R_m	R_w
Os02g0553200(*APX8*)	homozygous	DEL:20871237	chr02:20870729-20871877	14.89%	0.84%
Os02g0553200(*APX8*)	homozygous	DEL:20871237	chr02:20871269-20871399	12.62%	1.98%
Os05g0170000(*BC10*)	mixture [a]	DEL:4197198<br>DEL:4197199-4197203<br>DEL:4197189-4197208/ DEL:4197198	chr05:4196904-4198079	52.54%	0.00%
Os05g0418100(*MLO9*)	homozygous	IN:20490025-20490026:G	chr05:20489828-20489944	30.79%	11.62%
Os10g0471100(*WDA1*)	homozygous	DEL:17453731	chr10:17453792-17453945	56.50%	1.77%
Os10g0471100(*WDA1*)	biallelic	DEL:17453731/DEL:17453722-17453743	chr10:17453792-17453945	47.25%	1.77%
Os10g0471100(*WDA1*)	homozygous	DEL:17453722-17453743	chr10:17453792-17453945	37.72%	1.77%
Os10g0471100(*WDA1*)	biallelic	DEL:17453731/DEL:17453731-17453734	chr10:17453792-17453945	45.20%	1.77%
Os10g0555700(*EXPB2*)	homozygous	IN:21836821-21836822:T	chr10:21836633-21836737	11.58%	0.02%

[a] RT-PCR-seq products were mixed prior to sequencing
