# Supplementary material for: In rice splice variants that restore the reading frame after frameshifting indel introduction are common, often induced by the indels and sometimes lead to organism-level rescue
Source: PLoS Genet. 2022 Feb 18;18(2):e1010071. doi: 10.1371/journal.pgen.1010071 (PMC8893660; doi:10.1371/journal.pgen.1010071)
Supplement: S7 Table — (PDF) [file pgen.1010071.s021.pdf]

S7 Table. Downloaded RNA-seq data used in this analysis.

Sample ID	Variety	Stage	Tissue	Sequencing Platform	Data size (G)	Read length	Datafile (SRA accession) ID
SM001	9311	Seedling	Shoots	Illumina Genome Analyzer II	6.07	40~76	ERR008647 ERR008648 ERR008653 ERR008654 ERR008659 ERR008660
SM002	9311	N.A.	Callus	Illumina Genome Analyzer	14.03	35~75	SRR037711 SRR037712 SRR037713 SRR037714 SRR037715 SRR037716 SRR037717 SRR037718 SRR037719 SRR037720 SRR037721 SRR037722 SRR037723 SRR037724
SM003	9311	Booting	Panicle	Illumina Genome Analyzer	13.58	35~75	SRR037725 SRR037726 SRR037727 SRR037728 SRR037729 SRR037730 SRR037731 SRR037732 SRR037733 SRR037734 SRR037735 SRR037736 SRR037737 SRR037738 SRR037739
SM004	9311	Seedling	Shoots	Illumina Genome Analyzer	0.14	35	SRR037740
SM005	9311	Seedling	Root	Illumina Genome Analyzer	0.18	35	SRR037741
SM006	9311	Tillering	Leaf	Illumina Genome Analyzer	0.20	35	SRR037742
SM007	9311	Flowerin	Panicle	Illumina Genome Analyzer	0.19	35	SRR037743
SM008	9311	Flowerin	Leaf	Illumina Genome Analyzer	0.19	35	SRR037744
SM009	9311	Filling	Panicle	Illumina Genome Analyzer	0.20	35	SRR037745
SM010	LYP9	Filling-E	Leaf	Illumina Genome Analyzer IIx	0.62	36	SRR1609321
SM011	LYP9	Filling-	Leaf	Illumina Genome Analyzer IIx	0.37	36	SRR1609322
SM012	LYP9	Filling-L	Leaf	Illumina Genome Analyzer IIx	0.39	36	SRR1609323

SM013	N2Y6	Filling-E	Leaf	Illumina Genome Analyzer IIx	0.60	36	SRR1609318
SM014	N2Y6	Filling-	Leaf	Illumina Genome Analyzer IIx	0.26	36	SRR1609319
SM015	N2Y6	Filling-L	Leaf	Illumina Genome Analyzer IIx	0.42	36	SRR1609320
SM016	Pa64	N.A.	N.A.	Illumina HiSeq 2000	14.16	49	SRR923813 SRR923816 SRR923817
SM017	Guangluai4	Seedling	Shoots	Illumina Genome Analyzer II	6.14	40~76	ERR008649 ERR008650 ERR008655 ERR008656 ERR008661 ERR008662
SM018	Kasalath	Seedling	Shoots	Illumina HiSeq 2000	10.41	101	DRR001372
SM019	Nipponbare	Seedling	Shoots	Illumina Genome Analyzer II	6.09	40~76	ERR008651 ERR008652 ERR008657 ERR008658 ERR008663 ERR008664
SM020	Nipponbare	Flowerin	Leaf	Illumina HiSeq 2000	55.80	150	ERR855945
SM021	Nipponbare	Flowerin	Panicle	Illumina HiSeq 2000	58.60	150	ERR855947
SM022	Nipponbare	Flowerin	Buds	Illumina HiSeq 2000	53.40	101	SRR1213581
SM023	Nipponbare	Flowerin	Flower	Illumina HiSeq 2000	56.11	101	SRR1213582
SM024	Nipponbare	Flowerin	Leaf	Illumina HiSeq 2000	58.53	101	SRR1213583
SM025	Nipponbare	Flowerin	Leaf	Illumina HiSeq 2000	56.58	101	SRR1213584
SM026	Nipponbare	Flowerin	Root	Illumina HiSeq 2000	52.57	101	SRR1213585
SM027	Nipponbare	Flowerin	Root	Illumina HiSeq 2000	58.17	101	SRR1213586
SM028	Nipponbare	Filling	Grains	Illumina HiSeq 2000	52.26	101	SRR1213587
SM029	Nipponbare	Ripening	Seed	Illumina HiSeq 2000	58.42	101	SRR1213588
SM030	Nipponbare	N.A.	Leaf	Illumina HiSeq 2000	22.21	150	SRR4241116
SM031	Nipponbare	N.A.	Callus	Illumina HiSeq 2000	21.51	150	SRR4241117
SM032	Nipponbare	N.A.	Callus	Illumina HiSeq 2000	20.69	150	SRR4241118
SM033	Nipponbare	Seedling	Root	Illumina HiSeq 2000	5.43	100	SRR1616169
SM034	Nipponbare	Seedling	Root	Illumina HiSeq 2000	5.16	100	SRR1616570
SM035	Nipponbare	Seedling	Root	Illumina HiSeq 2000	3.56	100	SRR1616571
SM036	Nipponbare	Seedling	Shoots	Illumina HiSeq 2000	3.76	100	SRR1616572
SM037	Nipponbare	Seedling	Shoots	Illumina HiSeq 2000	4.01	100	SRR1616573
SM038	Nipponbare	Seedling	Shoots	Illumina HiSeq 2000	3.85	100	SRR1616574
SM039	TP309	Seedling	Leaf	Illumina HiSeq 2000	19.10	100	ERR194168

SM040	Nipponbare x Kasalath	Seedling	Shoots	Illumina HiSeq 2000	10.10	101	DRR001373
SM041	Kasalath x Nipponbare	Seedling	Shoots	Illumina HiSeq 2000	13.20	101	DRR001375
SM042	Oryza barthii	N.A.	Leaf	Illumina Genome Analyzer IIx	9.63	120	SRR1170742
SM043	Oryza barthii	N.A.	Root	Illumina Genome Analyzer IIx	9.12	120	SRR1170744
SM044	Oryza barthii	N.A.	Panicle	Illumina Genome Analyzer IIx	48.64	100	SRR1170762
SM045	Oryza glaberrima	N.A.	Leaf	Illumina HiSeq 2000	43.30	100	SRR1174376
SM046	Oryza glaberrima	N.A.	Panicle	Illumina HiSeq 2000	25.45	100	SRR1174378
SM047	Oryza glaberrima	N.A.	Root	Illumina HiSeq 2000	37.55	100	SRR1174379
SM048	Oryza meridionalis	N.A.	Panicle	Illumina HiSeq 2000	50.40	100	SRR1174791
SM049	Oryza meridionalis	N.A.	Root	Illumina HiSeq 2000	49.53	100	SRR1174792
SM050	Oryza meridionalis	N.A.	Leaf	Illumina HiSeq 2000	51.61	100	SRR1174790
SM051	Oryza rufipogon	N.A.	Leaf	Illumina HiSeq 2000	41.80	100	SRR1220645
SM052	Oryza rufipogon	N.A.	Panicle	Illumina HiSeq 2000	38.40	100	SRR1220646
SM053	Oryza rufipogon	N.A.	Root	Illumina HiSeq 2000	19.60	100	SRR1220647
SM054	W0106	Seedling	Shoots	Illumina HiSeq 2000	14.70	101	DRR001374
SM055	W1921	Seedling	Shoots	Illumina HiSeq 2000	11.10	101	DRR001371

N.A. means “not available”
