# Supplementary material for: In rice splice variants that restore the reading frame after frameshifting indel introduction are common, often induced by the indels and sometimes lead to organism-level rescue
Source: PLoS Genet. 2022 Feb 18;18(2):e1010071. doi: 10.1371/journal.pgen.1010071 (PMC8893660; doi:10.1371/journal.pgen.1010071)
Supplement: S8 Table — (PDF) [file pgen.1010071.s022.pdf]

S8 Table. The numbers of different types of AS events detected by downloaded RNA-seq data.

Types of AS events	Detected number
Exon skipping	23,338
Alternative 5' splice	90,462
Alternative 3' splice	130,761
Alternative 5' and 3' splice	44,272
Undefinable type(both splice sites on the annotated exon)	113,397
Total	402,230
