# Supplementary material for: In rice splice variants that restore the reading frame after frameshifting indel introduction are common, often induced by the indels and sometimes lead to organism-level rescue
Source: PLoS Genet. 2022 Feb 18;18(2):e1010071. doi: 10.1371/journal.pgen.1010071 (PMC8893660; doi:10.1371/journal.pgen.1010071)
Supplement: S9 Table — (PDF) [file pgen.1010071.s023.pdf]

S9 Table. Junctions detected in 55 downloaded RNA-seq datasets.

Appearance of a junction in multiple independent datasets	No. of annotated junction	$PSI_{annotated}$ (mean ± SEM)	No. of novel junctions (3n, non-3n)	PSI_{novel} (mean ± SEM)	Total
No. Datasets ≥ 1	115844	0.87782 ± 0.00028	402230（146259, 255971）	0.16302 ± 0.00039	518074
No. Datasets ≥ 2	113732	0.87721 ± 0.00028	209407（72390, 137017）	0.16599 ± 0.00041	323139
No. Datasets ≥ 10	106634	0.86905 ± 0.00035	57541（18420, 39121）	0.19139 ± 0.00055	164175
