# Supplementary material for: In rice splice variants that restore the reading frame after frameshifting indel introduction are common, often induced by the indels and sometimes lead to organism-level rescue
Source: PLoS Genet. 2022 Feb 18;18(2):e1010071. doi: 10.1371/journal.pgen.1010071 (PMC8893660; doi:10.1371/journal.pgen.1010071)
Supplement: S10 Table — (PDF) [file pgen.1010071.s024.pdf]

S10 Table. Primers for cDNA amplification in Nipponbare reference genome.

Gene locus	Primer-Forward	Primer-Reverse
Os01g0848400	CCGCACAGCCCTACTCTA	ATCACCCGACAAAATCATG
Os01g0883800	TGTGCGACCTGAGGATG	TGTGCAGGCAGCTCTTATA
Os01g0922800	GTCGGCAAGCTCTACGAGTA	CCTAGAGCCACTGGAGTTTG
Os01g0930400	CGAGCCTCTGCACACAAG	CTACCCGCAACAGTCTATTAT
Os02g0301100	TCTCGCCGGACACCAT	CCACTTTGCTAGTAAGTCATGA
Os03g0122600	CGCCTCCACCACCACTCCT	GCATCGCTTGGCTATCTTC
Os03g0387100	GCGGCGGAAGCAATCGTAGT	TCGTGTCACTGGTGCCAATC
Os03g0650000	ATGCCTGAAGTCCACTGCTC	CTTTGAACCTTTTGGGGACTA
Os03g0718600	GCCAGGCTCCACCAACGC	TGGGGTCTGTCTCAAACTCAG
Os03g0762000	ACCCACCCAGCAGCAGCGT	GTGGGGCTTGACATCTCGAT
Os04g0656500	GTGTGTGTGCGACTGCGTGA	CATCGCCTGTCCAAGCTGAA
Os05g0158500	TCCTCCTCCATTTCCATCCA	TGTTGAGGGGAACCAGGTGAC
Os06g0157700	AAGTGGCAGGGACAGGGACC	TCGGGATCATCGTTAGCTAG
Os06g0183100	ATGCTTCTGGGTGCTTTGAG	TTCATATGCAGGCACCAAGT
Os06g0650300	GGTGAGGGTGAGGGAGTTCG	GTCCGGGTTGAGGTTCTTGA
Os07g0695100	TCGTCGTCATTCTGCCACCA	AGCTTGCCGCACGAACTGTC
Os08g0485500	GCCATTTTGACCAGACCACC	CAGGGTCAGGGGTCGAACAG
Os09g0369400	TCTTGGGGTGTTCTTGACTG	CGATCAGCGTCACGGGTTAC
Os09g0439800	TCTCCCTCCCCAATCTCTCA	CTGGCATGAACGAACTAACA
Os09g0441900	GAGGGGTGGTTCTGAGTTG	AGACAGCATGGGTTACGACA
	GAGGGGTGGTTCTGAGTTG	TGACAAGTAATGTAAGAGGAC
	ATGGGACCGTTCGTACTGG	AGACAGCATGGGTTACGACA
	CATTGTATATTTGTGGTGGA	ACTTTCAGACTTTCAATTCC
