# Supplementary material for: In rice splice variants that restore the reading frame after frameshifting indel introduction are common, often induced by the indels and sometimes lead to organism-level rescue
Source: PLoS Genet. 2022 Feb 18;18(2):e1010071. doi: 10.1371/journal.pgen.1010071 (PMC8893660; doi:10.1371/journal.pgen.1010071)
Supplement: S11 Table — (PDF) [file pgen.1010071.s025.pdf]

S11 Table. Comparison of junctions detected from downloaded RNA-seq datasets and local RT-PCR-seq data. Only RNA-seq datasets for Nipponbare were used here to ensure comparability.

Gene locus	No. of annotated junctions	No. of total junctions detected			No. of non-3n junctions detected		
		RNA-seq	RT-PCR-seq	Shared between RNA-seq and RT-PCR-seq	RNA-seq	RT-PCR-seq	Shared between RNA-seq and RT-PCR-seq
Os01g0848400	3	16	12	8	9	5	3
Os01g0883800	3[a]	9	10	5	5	6	2
Os01g0922800	4	15	15	9	8	9	5
Os01g0930400	8	10	12	8	2	1	0
Os02g0301100	4	47	12	5	25	5	1
Os03g0122600	7[b]	21	21	10	10	9	4
Os03g0387100	10	16	23	13	5	7	2
Os03g0650000	5[b]	17	18	13	9	10	7
Os03g0718600	5	11	20	11	3	7	3
Os03g0762000	3	7	12	6	2	4	1
Os04g0656500	2	8	7	3	1	2	0
Os05g0158500	9	30	16	13	12	5	2
Os06g0157700	3	10	6	4	4	3	1
Os06g0183100	5	10	14	9	3	5	2
Os06g0650300	2	3	2	2	1	0	0
Os07g0695100	9	39	47	30	16	24	11
Os08g0485500	1	2	5	2	1	3	1
Os09g0369400	6	31	24	10	17	9	3
Os09g0439800	4	9	5	5	2	0	0
Os09g0441900	3	7	5	4	4	2	1
Total	96	318	286	170	139	116	49
Novel junctions	--	225	192	77	139	116	49

[a] One annotated junction was not found in the downloaded datasets; [b] One annotated junction was not found in both downloaded datasets and local RT-PCR-seq data.
