# Supplementary material for: In rice splice variants that restore the reading frame after frameshifting indel introduction are common, often induced by the indels and sometimes lead to organism-level rescue
Source: PLoS Genet. 2022 Feb 18;18(2):e1010071. doi: 10.1371/journal.pgen.1010071 (PMC8893660; doi:10.1371/journal.pgen.1010071)
Supplement: S12 Table — (PDF) [file pgen.1010071.s026.pdf]

S12 Table. Comparison of "non-canonical depth/ canonical depth" between WT and CRISPR experiments and "rescue junction depth/ canonical depth" of rescued genes.

Metrics	Data source	NO. of genes investigated	% of genes＞0.1	%of genes＞0.5
non-canonical depth/ canonical depth	RNA-seq	37,858	40.6%	24.4%
	CRISPR mutants	73	39.7%	27.4%
rescue junction depth/ canonical depth	CRISPR mutants	39	15.4%	5.1%
