# Supplementary material for: In rice splice variants that restore the reading frame after frameshifting indel introduction are common, often induced by the indels and sometimes lead to organism-level rescue
Source: PLoS Genet. 2022 Feb 18;18(2):e1010071. doi: 10.1371/journal.pgen.1010071 (PMC8893660; doi:10.1371/journal.pgen.1010071)
Supplement: S13 Table — (PDF) [file pgen.1010071.s027.pdf]

S13 Table. Predicted effects of AS on Nipponbare and Tetep homologous genes.

Predicted effects	Nipponbare gene ID	Intron number	Tetep gene ID	Intron number	Novel splice junction which could avoid premature termination
AS circumvent PTC caused by non-3n indel	LOC_Os01g57270	0	chr01.fgenesh2994	2	Tetep-chr01: 27024079-27025751
	LOC_Os07g12680	1	chr07.fgenesh729	1	Nipponbare-chr07:7244811-7244894
	LOC_Os08g07950	11	chr08.fgenesh381	3	Tetep-chr07:3293512-3293635
	LOC_Os08g13800	4	chr08.fgenesh557	3	Nipponbare-chr08:8235088-8235122
	LOC_Os11g12050	3	chr11.fgenesh843	2	Tetep-chr11:6691925-6692246
	LOC_Os11g35210	4	chr11.fgenesh1737	3	Nipponbare-chr11:20644706-20644786
	LOC_Os12g10410	1	chr12.fgenesh59	7	Tetep-chr12:565730-565814
	LOC_Os12g17410	3	chr12.fgenesh322	1	Nipponbare-chr12:9984198-9984315
	LOC_Os08g14830	2	tig00011639.fgenesh73	3	Tetep- tig00011639:728971-729027
	LOC_Os11g46210	2	chr11.fgenesh2457	1	Tetep-chr11:27066566-27066730
AS circumvent PTC caused by nonsense mutation	LOC_Os06g22460	1	chr06.fgenesh1561	0	Nipponbare -chr06:13055756-13055892
	LOC_Os06g33360	1	chr06.fgenesh1936	2	Tetep-chr06:19406322-19407525
	LOC_Os11g29520	6	chr11.fgenesh1360	2	Nipponbare-chr11:17131686-17131916
	LOC_Os11g34880	2	chr11.fgenesh1707	3	Tetep-chr11:19982301-19982461
AS circumvent PTC caused by nonsense mutation and non-3n indel	LOC_Os01g40030	4	chr01.fgenesh1832	0	Nipponbare-chr01:22580139-22580227 (nonsense mutation relative)<br>Nipponbare-chr01:22579707-22579875 (non-3n indel relative)
	LOC_Os05g23990	0	chr05.fgenesh1166	3	Tetep-chr05:13888183-13888489 (non-3n indel relative)<br>Tetep-chr05:13889273-13889343 (nonsense mutation relative)
	LOC_Os08g16070	2	tig00011639.fgenesh2	1	Tetep- tig00011639:17092-17421 (non-3n indel relative)<br>Nipponbare-chr08:9796536-9796726 (nonsense mutation relative)
no AS or AS has no effect on gene transcript	LOC_Os01g39990	0	chr01.fgenesh1829	0	
	LOC_Os02g18080	0	chr02.fgenesh1256	0	
	LOC_Os05g30220	0	chr05.fgenesh1360	1	

LOC_Os05g31610	0	tig00012122.fgenesh108	0
LOC_Os06g03500	1	chr06.fgenesh172	1
LOC_Os06g41480	1	chr06.fgenesh2404	0
LOC_Os07g33720	0	chr07.fgenesh1750	0
LOC_Os08g42670	3	chr08.fgenesh2129	2
LOC_Os10g04510	0	tig00001023.fgenesh139	0
LOC_Os11g28470	0	tig00011558.fgenesh14	0
LOC_Os11g28950	3	chr11.fgenesh1320	0
LOC_Os11g29110	2	chr11.fgenesh1328	2
LOC_Os11g35580	1	tig00011805.fgenesh34	1
LOC_Os11g43250	0	chr11.fgenesh2190	1
LOC_Os11g43390	0	chr11.fgenesh2200	2
LOC_Os11g45050	0	chr11.fgenesh2340	0
LOC_Os11g39310	2	chr11.fgenesh1896	2
LOC_Os12g31160	1	chr12.fgenesh923	3
