# Supplementary material for: In rice splice variants that restore the reading frame after frameshifting indel introduction are common, often induced by the indels and sometimes lead to organism-level rescue
Source: PLoS Genet. 2022 Feb 18;18(2):e1010071. doi: 10.1371/journal.pgen.1010071 (PMC8893660; doi:10.1371/journal.pgen.1010071)
Supplement: S14 Table — (PDF) [file pgen.1010071.s028.pdf]

S14 Table. Comparisons between Tophat and StringTie in identifying splice junctions.

Sample ID	No. of junctions detected by Tophat	No. of junctions detected by StringTie	Tophat-only junctions			
			Number	% in all Tophat detected junctions	No. of junctions appeared in ≥2 datasets	Junctions appeared in ≥2 datasets / Tophat-only junctions (%)
SM002	140349	111441	28908	20.6	26503	91.68
SM003	145940	110792	35148	24.08	32275	91.83
SM018	128808	105100	23708	18.41	22554	95.13
SM040	148999	115670	33329	22.37	31776	95.34
SM041	140650	116355	24295	17.27	23428	96.43
SM045	143164	101016	42148	29.44	37133	88.1
SM023	246109	139013	107096	43.52	91062	85.03
SM024	238084	126997	111087	46.66	95373	85.85
SM027	244249	127684	116565	47.72	98524	84.52
SM029	218753	133764	84989	38.85	71328	83.93
SM048	151474	108710	42764	28.23	36826	86.11
SM050	148934	98989	49945	33.53	42570	85.23
SM042	109619	82630	26989	24.62	25888	95.92
SM044	174596	118374	56222	32.2	49054	87.25
SM034	153414	115577	37837	24.66	36151	95.54
