# Supplementary material for: In rice splice variants that restore the reading frame after frameshifting indel introduction are common, often induced by the indels and sometimes lead to organism-level rescue
Source: PLoS Genet. 2022 Feb 18;18(2):e1010071. doi: 10.1371/journal.pgen.1010071 (PMC8893660; doi:10.1371/journal.pgen.1010071)
Supplement: S15 Table — (PDF) [file pgen.1010071.s029.pdf]

S15 Table. Primers used in the construction of amiRNA vectors.

Primer	Sequence
UBI-maize-U	GTACCCGGGGATCCGggcgcgccgcttgcatgcctgcagtgca
UBI-maize-L	CTGGGATCCGAGGGGGATCCcctagggctgcagaagtaaca
Pnw55-U	tgttacttctgcagccctaggGGATCCCCCTCGGATCCCAG
Pnw55-L	CCAAATGCTGCAGGTCGACGggcgcgccGGTACCGCTGCTGATGCTGA
1-UPF1-I	agTTCGTACCACTATAAGCTCAAcaggagattcagtttga
1-UPF1-II	tgTTGAGCTTATAGTGGTACGAActgctgctgctacagcc
1-UPF1-III	ctTTGAGGTTAAAGTGGTACGAAttcctgctgctaggctg
1-UPF1-IV	aaTTCGTACCACTTTAACCTCAAagagaggcaaaagtgaa
2-UPF1-I	agTAATGAGACATTCAGGCTCTAcaggagattcagtttga
2-UPF1-II	tgTAGAGCCTGAATGTCTCATTActgctgctgctacagcc
2-UPF1-III	ctTAGAGGCTGTATGTCTCATTAttcctgctgctaggctg
2-UPF1-IV	aaTAATGAGACATACAGCCTCTAagagaggcaaaagtgaa
1-UPF2-I	agTATTTACGTGACTCCAGGCTCcaggagattcagtttga
1-UPF2-II	tgGAGCCTGGAGTCACGTAAATActgctgctgctacagcc
1-UPF2-III	ctGAGCCAGGACTCACGTAAATAttcctgctgctaggctg
1-UPF2-IV	aaTATTTACGTGAGTCCTGGCTCagagaggcaaaagtgaa
2-UPF2-I	agTGAACATTGAATAACGTGCGCcaggagattcagtttga
2-UPF2-II	tgGCGCACGTTATTCAATGTTCActgctgctgctacagcc
2-UPF2-III	ctGCGCAGGTTTTTCAATGTTCAttcctgctgctaggctg
2-UPF2-IV	aaTGAACATTGAAAAACCTGCGCagagaggcaaaagtgaa
1-UPF3-I	agTAAACCAAAATGAGATGCCGCcaggagattcagtttga
1-UPF3-II	tgGCGGCATCTCATTTTGGTTTActgctgctgctacagcc
1-UPF3-III	ctGCGGCTTCTGATTTTGGTTTAttcctgctgctaggctg
1-UPF3-IV	aaTAAACCAAAATCAGAAGCCGCagagaggcaaaagtgaa
2-UPF3-I	agTTAATAATTCTGCCACTCCCCcaggagattcagtttga
2-UPF3-II	tgGGGGAGTGGCAGAATTATTAActgctgctgctacagcc
2-UPF3-III	ctGGGGACTGGGAGAATTATTAAttcctgctgctaggctg
2-UPF3-IV	aaTTAATAATTCTCCCAGTCCCCagagaggcaaaagtgaa
1-SMG7-I	agTTACCAAAGGCGAAAAGCCTCcaggagattcagtttga
1-SMG7-II	tgGAGGCTTTTCGCCTTTGGTAActgctgctgctacagcc
1-SMG7-III	ctGAGGCATTTGGCCTTTGGTAAttcctgctgctaggctg
1-SMG7-IV	aaTTACCAAAGGCCAAATGCCTCagagaggcaaaagtgaa
2-SMG7-I	agTAACTAACGCTAGCGGACCGAcaggagattcagtttga
2-SMG7-II	tgTCGGTCCGCTAGCGTTAGTTActgctgctgctacagcc
2-SMG7-III	ctTCGGTGCGCAAGCGTTAGTTAttcctgctgctaggctg
2-SMG7-IV	aaTAACTAACGCTTGCGCACCGAagagaggcaaaagtgaa
1-UPF1-like-I	agTTCAAGCGATAAACTACACGTcaggagattcagtttga

1-UPF1-like-II	tgACGTGTAGTTTATCGCTTGAActgctgctgctacagcc
1-UPF1-like-III	ctACGTGAAGTATATCGCTTGAAttcctgctgctaggctg
1-UPF1-like-IV	aaTTCAAGCGATATACTTCACGTagagaggcaaaagtgaa
2-UPF1-like-I	agTTATGATCACTTAAGAATCCCcaggagattcagtttga
2-UPF1-like-II	tgGGGATTCTTAAGTGATCATAActgctgctgctacagcc
2-UPF1-like-III	ctGGGATACTTTAGTGATCATAAttcctgctgctaggctg
2-UPF1-like-IV	aaTTATGATCACTAAAGTATCCCagagaggcaaaagtgaa
