# Supplementary material for: In rice splice variants that restore the reading frame after frameshifting indel introduction are common, often induced by the indels and sometimes lead to organism-level rescue
Source: PLoS Genet. 2022 Feb 18;18(2):e1010071. doi: 10.1371/journal.pgen.1010071 (PMC8893660; doi:10.1371/journal.pgen.1010071)
Supplement: S16 Table — (PDF) [file pgen.1010071.s030.pdf]

S16 Table. Primers for cDNA amplification of NBS genes in Nipponbare and Tetep

Variety	Gene locus	Primer-Forward	Primer-Reverse
Nipponbare	LOC_Os01g57270	AGGTTTGGTTGTTCCTATTTCC	ATAGGAGAGTTTACAGAACACC
Nipponbare	LOC_Os07g12680	ATGGAGATTGTAACAGGAGC	ATAAGTTGGAAGTCTGCTAGTC
Nipponbare	LOC_Os11g12050	CTGTCATAGCCAAACTCTCC	ACTAGAGCGGTTTTTCCA
Nipponbare	LOC_Os11g35210	GCTGGCTGGCTTACATTTGG	AAGCCTTCATTTAGGGAAACCG
Nipponbare	LOC_Os12g17410	CATACCCTGCTGGTGAAGCT	CCTGCTTAATGTTGCGAACC
Nipponbare	LOC_Os08g14830	TTTCCGCGTCGTTGAGTC	GTTGCCTCCTCACATTGG
Nipponbare	LOC_Os11g46210	AGTTGGCGGTAGGTGCTT	TGAAGCGAACCAAGACCA
Nipponbare	LOC_Os06g33360	ACTCAGATTCCATTAGCTGGC	TTACAATTCTACCAGTTCGTGG
Nipponbare	LOC_Os11g29520	GGTTAGCTGGAGCTACGGT	CTGGCTTTCGTCTGTACATGAC
Nipponbare	LOC_Os11g34880	TCGATCTCTGCTTTCACTG	CAGTTCCTACATGCTCAAGTC
Nipponbare	LOC_Os01g39990	CTCATCACGAAGTACAGCA	CAAAGGTGAGGGAAAACAA
Nipponbare	LOC_Os05g30220	AGTGACTCCTATCCCAATTCC	GCAGGTTTGCCTTATCTTGC
Nipponbare	LOC_Os05g31610	TCATCAGGGAGGAGATGG	AAGCTCCTTCAATTCCGG
Nipponbare	LOC_Os06g03500	GCGAAGAGATGAGCTTGC	CATCGTCATGGACGGATTCC
Nipponbare	LOC_Os06g41480	TTGGATTTCTTGCTATCAGCAG	TAGTTGAGATGTTGCGTACC
Nipponbare	LOC_Os07g33720	CACCGAGCGTACCATACAC	AACTATGCATGCAATACAGGG
Nipponbare	LOC_Os08g42670	GGCGGCAACAGGACAAAG	GGCAAATCTCAAACAAGGG
Nipponbare	LOC_Os10g04510	ACAAAGCATCATACCCTGAG	AGCACTGCGAATTCTTCC
Nipponbare	LOC_Os11g28470	ATTCTTCGCGGATCTTGTG	ACTTCTGCTTCATTCGTTTGG
Nipponbare	LOC_Os11g29110	AGGATATTAGGGCTGGCTTTGG	AGGCACATGGGATATGTTGTC
Nipponbare	LOC_Os11g43250	GATGCTAAGCTTTCAATTACCG	CATGAACGGCTTCCTGGT
Nipponbare	LOC_Os11g43390	AGGAATTCTTGAGCTCCCT	TCCACCTCACCAAGTCCA
Nipponbare	LOC_Os11g45050	GATCGTTACAGACAAGGCA	CAAGTTGTGGTGGGAGAG
Nipponbare	LOC_Os11g39310	AGGGAGTACTCGTCTTATCG	AATGAACATGCAGCCTGC
Tetep	chr01.fgenesh2994	CAAATTCATCAACCGCGC	CCCTCAGGACTTGTTTGTCTC
Tetep	chr07.fgenesh729	GCATGCGACTAGATAACATAGC	GTAACAGGAGCAATAAACACTC
Tetep	chr08.fgenesh381	GGGTGATGAACACTCTCC	ATTTGCTTCACCCTCGAC
Tetep	chr08.fgenesh557	ATCGCCGTGAGTTTGGTG	CCATTGTCAAGATGAGCATCC

Tetep	chr11.fgenesh843	TGGGTAGCATATTGTTCATCC	AAATCAGCTCTTGAACATGC
Tetep	chr11.fgenesh1737	GCTGGCTGGCTTACATTTGG	AAGCCTTCATTTAGGGAAACCG
Tetep	chr12.fgenesh59	CATGATATTTGGCTTGCACTC	TCATCGTTTCGCTGATCG
Tetep	chr12.fgenesh322	AGTCTGATTCATGTGCACG	CCTTTCTGAGGGATGAGCTG
Tetep	tig00011639.fgenesh73	TTTCCGCGTCGTTGAGTC	GTTGCCTCCTCACATTGG
Tetep	chr11.fgenesh2457	ATGGAGTTGGTGGTAGGT	AGGAACTTGGTTATTGCTTCTG
Tetep	chr06.fgenesh1561	AAGCTGTAGCTTTACCAACTG	CTTGCTTGGGCTAGAGGGA
Tetep	chr06.fgenesh1936	CAAACTGACCGAGCTGCT	ATCTTTCTAGTTGAAGGGCG
Tetep	chr11.fgenesh1360	GGTTAGCTGGAGCTACGGT	GGACATCTAGATTCTCCCTTGC
Tetep	chr11.fgenesh1707	GCTCAAGTCAAGTGTGGG	CAACCTGCTCAAGGAGGA
Tetep	chr01.fgenesh1832	GGGTATTGGAGACCTCCA	GCATTAGCCAGTGATCTTGC
Tetep	chr05.fgenesh1166	CTGCTGTAGCATCTGGGCT	ATATGAGAGACAAGCTGCCAG
Tetep	tig00011639.fgenesh2	TTTGCTGTGAGGAAGATCG	GATATCAACACTGGTCTCAGAG
Tetep	chr01.fgenesh1829	CAGAGATTTATCTTTAGGCTGC	ACACACTCTTATCTGTAGTTGC
Tetep	chr02.fgenesh1256	TTTCAGTGGGCTAGGTCTACC	AGACCACCTCTGCTCATTGG
Tetep	chr05.fgenesh1360	TCAGTCAATACGCTTCCAACG	TAGCTTACGATTGGTAGCATCC
Tetep	tig00012122.fgenesh108	TTGCATCCGGAGCTGTGAG	ATGTGCACTGATGTCTCTTCTC
Tetep	chr06.fgenesh172	TCACTTGCCAGAGCTCTCTC	GCGAAGAGATGAGCTTGC
Tetep	chr06.fgenesh2404	TGTTACAGGATGGTTCTTGTC	TGTAAAGTCTTGTGAGACATGG
Tetep	chr07.fgenesh1750	TGGTTGGAGGCGGATTGAC	GACATCTGTTGGCATGCTACC
Tetep	chr08.fgenesh2129	ATCCTCCAGTTGGGTCCAC	GCATGATGAGCGTGACGAG
Tetep	tig00001023.fgenesh139	AGGTGATCTCACCAGTCG	CGGACAACGGTGATTCCA
Tetep	tig00011558.fgenesh14	ATTCTTCGCGGATCTTGTG	CGTTTGGTTGACTGCAATGAG
Tetep	chr11.fgenesh1320	CCCTGGCAATTCTCTCATCTC	GGCCGTTGTCCTAGATGCT
Tetep	chr11.fgenesh1328	ATGTACTTGAACAAGGCAGAG	AGGCACATGGGATATGTTGTC
Tetep	tig00011805.fgenesh34	GGGAACGAGGTAATCAACCA	CCCTCGATCTGTCAATGCAG
Tetep	chr11.fgenesh2190	GATGCTAAGCTTTCAATTACCG	CATGAACGGCTTCCTGGT
Tetep	chr11.fgenesh2200	AGGAATTCTTGAGCTCCCT	TCCACCTCACCAAGTCCA
Tetep	chr11.fgenesh2340	GATCGTTACAGACAAGGCA	CAAGTTGTGGTGGGAGAG
Tetep	chr11.fgenesh1896	CGGCGCTAATAATTTCCA	TCAGGAGAGGAGGACATCA
Tetep	chr12.fgenesh923	TGGGTGTGATGTTCAACC	TTGTCGAACTCCATGGTGAC
