# Supplementary material for: In rice splice variants that restore the reading frame after frameshifting indel introduction are common, often induced by the indels and sometimes lead to organism-level rescue
Source: PLoS Genet. 2022 Feb 18;18(2):e1010071. doi: 10.1371/journal.pgen.1010071 (PMC8893660; doi:10.1371/journal.pgen.1010071)
Supplement: S17 Table — (PDF) [file pgen.1010071.s031.pdf]

S17 Table. Information of the 40 rice cultivars downloaded from "The 3000 Rice Genomes Project"

Sample ID	Variety Group	Area of origin	Depth	Coverage
IRIS_313-10177	*Indica*	China	33.67	0.88
IRIS_313-11643	*Indica*	India	34.37	0.88
IRIS_313-11645	*Indica*	India	34.39	0.88
IRIS_313-11646	*Indica*	India	35.29	0.88
IRIS_313-11656	*Indica*	Indonesia	34.74	0.88
IRIS_313-11664	*Indica*	China	38.39	0.89
IRIS_313-11665	*Indica*	China	36.32	0.89
IRIS_313-11668	*Indica*	China	36.08	0.89
IRIS_313-11669	*Indica*	China	51.09	0.91
IRIS_313-11681	*Indica*	Thailand	34.8	0.88
IRIS_313-11686	*Indica*	Thailand	34.35	0.89
IRIS_313-11710	*Indica*	Thailand	34.09	0.89
IRIS_313-11717	*Indica*	Indonesia	34.52	0.88
IRIS_313-11722	*Indica*	Bangladesh	36.38	0.89
IRIS_313-11723	*Indica*	Guinea	40.18	0.89
IRIS_313-11731	*Indica*	China	33.69	0.89
IRIS_313-11801	*Indica*	China	43.1	0.89
IRIS_313-11805	*Indica*	China	36.96	0.89
IRIS_313-11812	*Indica*	Kenya	41.86	0.89
IRIS_313-11819	*Indica*	Myanmar	33.9	0.88
CX109	*Japonica*	Philippines	26.55	0.91
IRIS_313-10918	*Japonica*	Philippines	26.15	0.92
IRIS_313-11789	*Japonica*	Madagascar	31.67	0.88
B001	*Temperate_Japonica*	China	27.59	0.94
B204	*Temperate_Japonica*	China	27.22	0.91
B269	*Temperate_Japonica*	China	29.58	0.94
IRIS_313-11651	*Temperate_Japonica*	China	33.15	0.96
IRIS_313-11652	*Temperate_Japonica*	China	26.9	0.93
IRIS_313-11800	*Temperate_Japonica*	China	38.45	0.96
IRIS_313-11671	*Temperate_Japonica*	Nepal	42.84	0.89
IRIS_313-11747	*Temperate_Japonica*	China	26.56	0.88
IRIS_313-11102	*Tropical_Japonica*	Liberia	29.31	0.92
IRIS_313-11755	*Tropical_Japonica*	Liberia	32.33	0.93
IRIS_313-11657	*Tropical_Japonica*	Nigeria	33.93	0.88
IRIS_313-11674	*Tropical_Japonica*	Thailand	27.67	0.88
IRIS_313-11738	*Tropical_Japonica*	India	28.5	0.88
IRIS_313-11754	*Tropical_Japonica*	Madagascar	32.66	0.91
IRIS_313-11758	*Tropical_Japonica*	Ivory Coast	26.23	0.88
IRIS_313-11794	*Tropical_Japonica*	Madagascar	35.43	0.88
IRIS_313-11796	*Tropical_Japonica*	China	40.97	0.89
