# Supplementary material for: In rice splice variants that restore the reading frame after frameshifting indel introduction are common, often induced by the indels and sometimes lead to organism-level rescue
Source: PLoS Genet. 2022 Feb 18;18(2):e1010071. doi: 10.1371/journal.pgen.1010071 (PMC8893660; doi:10.1371/journal.pgen.1010071)
Supplement: S1 Text — (DOCX) [file pgen.1010071.s032.docx]

## S1 Text. No robust evidence for tissue specific splice junctions

The above evidence suggests that most genes have putative mode 2-4 isoforms. A putative rescue isoform might however be common in one sample but be highly tissue specific. The rescue isoform is likely to be seen in some of the same tissues as the wild type gene but need not be found in all such tissues. If the rescue form is not seen in the tissue or tissues in which it is needed, then it is unlikely to rescue. The issue then is what proportion of potential rescue isoforms are tissue specific?

One difficulty in appraising this is that different read depths across tissues can create spurious signals of absence. To address this, we take advantage of there being three biological replicates for root and three for shoot, all other variables the same. We randomly select one of the three from each and ask how many splice forms are replicable within the two tissue replicates and not between the two unselected replicates for the other tissue. For 55,246 novel junctions identified in root sample SM033, 38,636 of them could be found in either of its biological replicates SM034 and SM035, while 11.02% fewer (34,380) were found in either of the two shoot biological replicates SM037 and SM038. Similarly, of 48,250 junctions identified in shoot SM036, 34,707 were present in both of its replicates SM037 and SM038, while 6.33% fewer (32,511) were present in both of the two root replicates SM034 and SM035.

The above results suggest a low degree of tissue specificity when comparing two tissues. The degree of tissue specificity is largely not affected by removal of low read-depth forms. If we require at least 10 reads to detect a junction in each database, for 16,204 novel junctions identified in root sample SM033, 14,672 of them could be found in both of its biological replicates SM034 and SM035, while 15.75% fewer (12,361) were found in both of the two shoot biological replicates SM037 and SM038. Similarly, of 14,288 junctions identified in shoot SM036, 13,335 were present in both of its replicates SM037 and SM038, while 10.00% fewer (12,001) were present in both of the two root replicates SM034 and SM035 (S12 Fig).

While the results appear to be robust to exclusion of poorly expressed forms, it remains possible that large differences in expression levels between tissues lead to apparent tissue-specific forms. To address this, we use sample covariance ($C_{V}=\frac{\sigma}{\mu}*100\%$, where$\sigma$ is the standard deviation of all six replicates from both tissues, and $\mu$ is the mean) to quantify the fluctuation of expression levels among different samples. We found 5,778 genes have expression +/- 15% between the six samples. From the 5,778 genes of relatively uniform expression 14,797 novel junctions were identified in root sample SM033, 10,545 of them could be found in both of its biological replicates SM034 and SM035, while just 3.74% fewer (10,151) were found in both of the two shoot biological replicates SM037 and SM038. We note that 3.74% is a third of the prior estimate. Of 13,100 junctions identified in shoot SM036, 9,574 were present in both of its replicates SM037 and SM038, while very slightly more (9,613, 0.41%), not less, were present in both of the two root replicates SM034 and SM035. These results suggest that controlling for expression level removes all or nearly all apparent tissue specificity.

As an alternatively means to check this conclusion, we consider non-differentially expressed genes (non-DEG, defined as expression-level fold change ＜2, and t-test *P* value ＞0.05). There are ~7640 non-DEGs detected in the two tissues. From these 7640 non-DEGs, 17,237 novel junctions were identified in root sample SM033, 12,519 of them could be found in both of its biological replicates SM034 and SM035, while 3.31% fewer (12,104) were found in both of the two shoot biological replicates SM037 and SM038. Of 15,318 junctions identified in shoot SM036, 11480 were present in both of its replicates SM037 and SM038, while slightly higher (11,447, 0.29%) were present in both of the two root replicates SM034 and SM035. This too supports the result that any tissue-specific differences are largely accountable in terms of between-tissue expression level differences.

We conclude that alternative isoforms tend not to be tissue specific and hence it can be reasonable to presume that the putative rescue isoform will commonly be found (at some level) in the same tissues as the wild type version.
