# Supplementary material for: In rice splice variants that restore the reading frame after frameshifting indel introduction are common, often induced by the indels and sometimes lead to organism-level rescue
Source: PLoS Genet. 2022 Feb 18;18(2):e1010071. doi: 10.1371/journal.pgen.1010071 (PMC8893660; doi:10.1371/journal.pgen.1010071)
Supplement: S2 Text — (DOCX) [file pgen.1010071.s033.docx]

## S2 Text. Molecular evolutionary analysis supports the hypothesis that most exon extensions are noise

Above we have shown commonality of splice junctions, some of which at least have the potential to rescue otherwise gene disabling mutations. Some, as in the case of 3n +1 deletions in *wda1* and *bc10*, enable some degree of fitness or phenotypic recovery. Are these alternative splicing events (extensions, shortenings or skipping) likely to be so much noise or might they be functional and deterministic? It seems rather improbable that selection could favour the retention of rarely used splice forms on the off-chance of there being a non-3n indel requiring rescue. A priori, we expect most alternative junctions to be so much splicing noise, although no doubt some are true functional alternative exons. If just noise, then this would fit better a model of accidental recovery of fitness.

From evidence from regularity of 3n and non-3n, where we see approximately 2/3 are non-3n, we surmise most are likely to be the product of noisy splicing. We note however that we see a very slight, but significant excess of 3n junctions suggestive of a small degree of functionality. In addition, we find the alternative exons to be rare. A further way to ask whether exon extensions might be noise is to consider the rate of evolution, measured by the degree of difference between *indica* and *japonica*, or the extent polymorphism within each.

We consider canonical exons (CE), the unaffected exonic parts of modified exons (UE), skipped exons (SE), alternative 5’-exon extension (A5E), alternative 5’-exon shortening (A5S), alternative 3’-exon extension (A3E), alternative 3’-exon shortening (A3S) and unaffected intronic parts adjacent to non-canonical exon regions (UI) (S10 Fig, S13 Fig). The most informative instance to test the hypothesis that alternative splicing is owing to noise are the exon extensions. Assuming the canonical annotation specifies the main mode of functional splicing, unlike exon shortening, if the extensions (A5E, A3E) are functional, we expect them to have a polymorphism/evolutionary rate below that of introns. As the extent to which an exon is employed we expect to be a predictor of the degree of selective constraint [1], assuming it to be functional, we would also expect A5E and A3E polymorphism/evolutionary rate to be higher than that of CE and UE even if they are functional. Conversely if A5E and A3E are largely splice noise they should have rates comparable to introns.

As different nucleotides have different mutabilities, it is important to control for differences in nucleotide content between the compartments. For all classes of mutation, excepting C:G->A:T per C:G in A5E, the mutational profile of A5E and A3E is the same as that in introns, while the rates are lower in CE and UE. C:G->G:C and A:T-> T:A for example have no difference between extension and introns but lower rates in CE and UE (Figures S13E-13F). This is broadly consistent with expectations were exon extensions largely under no more selection than intronic sequence. Consistent with this we also see no difference between extensions that are multiples of three and extensions that are not multiples of three long (Diversity of 3n A5E is 0.001969, non-3n is 0.002020, t-test, *P* = 0.76; Diversity of 3n A3E is 0.002088, non-3n is 0.001984, t-test, *P* = 0.45), while a priori a selectionist model would predict greater usage and tolerance of exons that are multiples of three as these maintain the reading frame. The cause of the slightly lower rate specifically of C:G->T:A and C:G->A:T in A5E compared with introns is suggestive of more complex mutational biases, possibly associated with differences in methylation (see S14 Fig).

Unlike A5E and A3E, the mutational profile of other regions as skipped exon (SE) and shortening (A5S and A3S) is the same as the CE and UE, and significantly lower than UI. That the exon regions associated with shortening have normal rates of evolution is as expected were the longer canonical form the functional form and the shortening a deleterious noisy event. That skipped exons have normal rates of evolution similarly suggests that canonical annotation specifies the normal functional mode of the protein.

# References

1. Pál C, Papp B, Hurst LD. Highly expressed genes in yeast evolve slowly. Genetics. 2001;158: 927–931.
