# Supplementary material for: In rice splice variants that restore the reading frame after frameshifting indel introduction are common, often induced by the indels and sometimes lead to organism-level rescue
Source: PLoS Genet. 2022 Feb 18;18(2):e1010071. doi: 10.1371/journal.pgen.1010071 (PMC8893660; doi:10.1371/journal.pgen.1010071)
Supplement: S3 Text — (DOCX) [file pgen.1010071.s034.docx]

## S3 Text. Comparison between two software, Tophat and StringTie, in identifying splice junctions

A cohort of 15 RNA-seq datasets were randomly selected from the 55 downloaded databases we used, and the junctions were detected independently via Tophat and StringTie. The results showed that generally Tophat reported about 17%~47% more junctions than StringTie. However, around 85%~96% of the Tophat-only junctions could be repeatedly detected in two or more datasets from all of those 55 downloaded databases by Tophat, suggesting they are more likely reliable junctions but get filtered by StringTie due to possible too stringent criteria by default (S15 Table). In conclusion, the results of Tophat covered nearly all of that could be detected by StringTie, and also included those over-filtered by StringTie. So the Tophat results could give more comprehensive charactering of those commonly assumed “noisy” junctions.
